# Discovery of a new potent oxindole multi-kinase inhibitor among a series of designed 3-alkenyl-oxindoles with ancillary carbonic anhydrase inhibitory activity as antiproliferative agents

**DOI:** 10.1186/s13065-023-00994-3

**Published:** 2023-07-18

**Authors:** Rania S. M. Ismail, Ahmed M. El Kerdawy, Dalia H. Soliman, Hanan H. Georgey, Nagwa M. Abdel Gawad, Andrea Angeli, Claudiu T. Supuran

**Affiliations:** 1 Department of Pharmaceutical Chemistry, Faculty of Pharmacy, Egyptian Russian University, P.O. Box 11829, Badr City, Cairo Egypt; 2 Department of Pharmaceutical Chemistry, Faculty of Pharmacy, Cairo University, Kasr El-Aini Street, P.O. Box 11562, Cairo, Egypt; 3 Department of Pharmaceutical Chemistry, School of Pharmacy, Newgiza University (NGU), Newgiza, km 22 Cairo–Alexandria Desert Road, Cairo, Egypt; 4 Department of Pharmaceutical Chemistry, Faculty of Pharmacy, Al-Azhar University, P.O. Box 11471, Cairo, Egypt; 5 Department of Pharmaceutical Chemistry, Faculty of Pharmacy and Drug Technology, Egyptian Chinese University, Cairo, 11786 Egypt; 6 Department of NEUROFARBA, Section of Pharmaceutical and Nutraceutical Sciences, University of Florence, Florence, Italy

**Keywords:** Design, Indolin-2-one, Carbonic anhydrase, Protein kinases, Synthesis, Docking

## Abstract

An optimization strategy was adopted for designing and synthesizing new series of 2-oxindole conjugates. Selected compounds were evaluated for their antiproliferative effect in vitro against NCI-60 cell lines panel, inhibitory effect on carbonic anhydrase (CA) isoforms (hCAI, II, IX and XII), and protein kinases. Compounds **5** and **7** showed promising inhibitory effects on hCA XII, whereas compound **4d** was the most potent inhibitor with low nanomolar CA inhibition against all tested isoforms. These results were rationalized by using molecular docking. Despite its lack of CA inhibitory activity, compound **15c** was the most active antiproliferative candidate against most of the 60 cell lines with mean growth inhibition 61.83% and with IC_50_ values of 4.39, 1.06, and 0.34 nM against MCT-7, DU 145, and HCT-116 cell lines, respectively. To uncover the mechanism of action behind its antiproliferative activity, compound **15c** was assessed against a panel of protein kinases (RET, KIT, cMet, VEGFR1,2, FGFR1, PDFGR and BRAF) showing % inhibition of 74%, 31%, 62%, 40%, 73%, 74%, 59%, and 69%, respectively, and IC_50_ of 1.287, 0.117 and 1.185 μM against FGFR1, VEGFR, and RET kinases, respectively. These results were also explained through molecular docking.

**Supplementary Information:**

The online version contains supplementary material available at 10.1186/s13065-023-00994-3.

## Introduction

Designing new compounds through optimization and hybridization strategies continuously produces effective agents that can overcome many side effects and pharmacokinetics problems associated with the use of classical ones [1–7]. This approach depends on the identification of the essential pharmacophoric entities in two or more biologically active molecules with successive merging of them into one molecular construction utilizing pre-selected properties in the parent candidates [1]. According to the mechanism of action and the biological targets of the selected parent molecules, the newly designed hybrids could exert their biological activity through one or several mechanisms of action [8].

Carbonic anhydrases (CAs) (EC 4.2.1.1) are zinc metalloenzymes that catalyze the reversible interconversion between carbon dioxide and bicarbonate ion using zinc as a metal cofactor. In humans, only α-CA isoforms can be found with sixteen distinct isozymes have been reported to date. CAs I–III, VA, VB, VII, XIII, and XV are intracellular isoforms, whereas the CAs IV, VI, IX, XII, and XIV isoforms are extracellular. The rest CAs (VIII, X, and XI) are catalytically inactive due to the absence of one or more histidine residues that are essential for the CA catalytic activity, these isoforms are known as carbonic anhydrase related proteins [9, 10]. The transmembrane isoforms CA IX and CA XII play a key role in balancing the extracellular pH [11]. To survive the hypoxia-induced acidosis characterizing tumor microenvironment, cancer cells overexpress CA IX in response to hypoxia-inducible factor-1a (HIF-1α), which does not exist in normal tissues, promoting cancer cell survival and progression under these conditions [12]. Whereas, CA XII is overexpressed in a broad spectrum of solid tumors such as breast, cervical, and lung cancer [13]. Carbonic anhydrase inhibition is a promising strategy for cancer treatment, especially through the transmembrane isoforms hCA IX and hCA XII inhibition [5, 7].

There are five known main categories of CA inhibitors (CAIs); (1) Zinc binders which coordinate the catalytically essential Zn^2+^ ion in CA active site (e.g., sulfonamides and their isosteres, carboxylates and hydroxamates) [14], (2) Inhibitors that anchor to the zinc-coordinated water molecule/hydroxide ion (e.g., phenols, carboxylate and polyamines) [15], (3) CA active site entry blockers (e.g., coumarins and their isosteres) [16], (4) Molecules that bind out of the active site cavity (e.g., a carboxylic acid derivatives) [17, 18], and finally, (5) Molecules possessing unknown inhibition mechanism (e.g., Imatinib and nilotinib) [15].

**Fig. 1:**
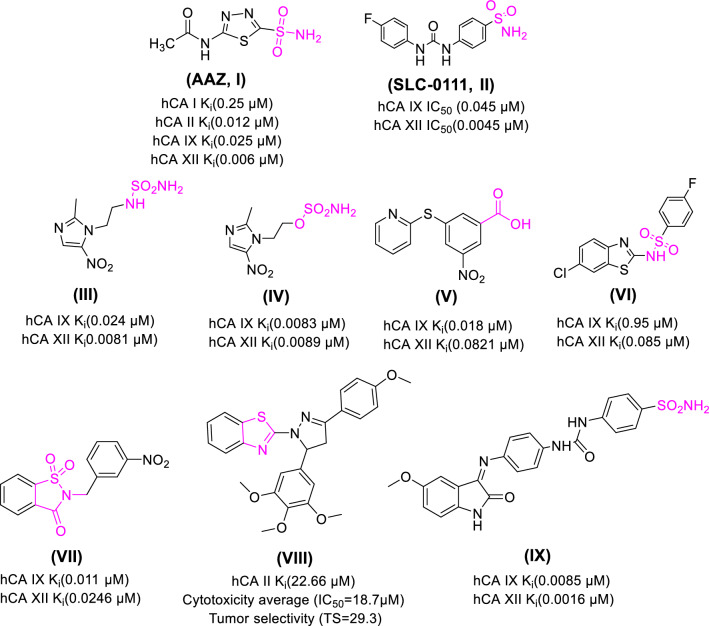
Structures of some reported anticancer CAIs, zinc binding groups are represented by pink color

Sulfonamide moiety (–SO_2_NH-R) and its isosteres (sulfamide, sulfamate and carboxylic group) such as compounds acetazolamide (**AAZ**, **I)**, SCL-0111** (II)**, and **III**-**V** (Fig. 1) are reported metal ion binders that coordinate the catalytically crucial Zn^2+^ ion in the CA active site known as zinc binding group (ZBG) [14, 19]. Primary sulfonamide is the most commonly used ZBG in designing CAIs, as they possess the essential features required for Zn^2+^ ion chelation and its adjacent amino acids binding. The deprotonated SO_2_NH^−^ moiety chelates the positively charged metal ion through its negative charge alternating the physiological zinc-bound nucleophile, moreover, the proton of the SO_2_NH^−^ moiety forms a H-bond with Thr199 [18].

Furthermore, the tail approach has been identified as a successful strategy for the design and development of promising and selective CAIs [7, 20]. Where further stabilization of the enzyme-inhibitor complex is achieved through several Van der Waals interactions taking place between the aromatic/heteroaromatic scaffold carrying the sulfonamide group and its nearby residues. Emerging new chemical scaffolds as potential drug candidates targeting CA isozymes created a plausible rational for cancer treatment [21].

One of the main challenges in the development of new antitumor CAIs has been the lack of isoform-selectivity found in most of classical CAIs [22]. Although primary sulfonamide is the most effective ZBG for carbonic anhydrase inhibition, it results in a non-selective inhibition and so, many accompanying side effects. Secondary sulfonamides (e.g., compound **VI**) maintain the ligands capability to chelate the Zn^2+^ ion in their deprotonated form, as proved by X-ray crystallography [23]. A similar inhibition pattern has been reported for the cyclic secondary sulfonamide saccharin and its derivatives (e.g., compound **VII**), with effective and selective inhibition [24–27].

Moreover, heterocyclic compounds bearing both nitrogen and sulfur atoms have coordination potential towards various transition metal ions, thus, they are noticeably represented in many bioactive coordination compounds, among these compounds, several electron rich polyfunctional thiazole derivatives [28–32]. Furthermore, incorporating a thiazole moiety into the molecular structure of many lead compounds resulted in an improvement in the biological activities of the newly synthesized molecules (e.g., compound **VIII**) [33, 34].

Oxindole is a privileged scaffold that represents the core of various biologically and therapeutically important compounds and one of the most interesting heterocyclic classes that possesses a promising activity profile, in particular, multi-targeted antiproliferative activity with good tolerability in humans [35]. Specifically, 3-alkenyl-oxindole derivatives showed potent antiproliferative activity as CAIs [36, 37] and multi-kinase inhibitors [38, 39]. Several aromatic sulfonamide derivatives incorporating oxindole moieties showed interesting selectivity against specific hCA isoforms, especially tumor associated hCA IX with Ki values in the single digit nanomolar range [40]. Additionally, the presence of a spacer between the benzenesulfonamide and 2-oxindole scaffolds resulted in CA inhibition with diverse activity and selectivity profiles [41, 42]. Benzenesulfonamide-indole derivatives with ureido linkage, e.g., compound **IX** (Fig. 1), showed remarkable inhibitory results against a panel of hCA isoforms [43].

**Fig. 2:**
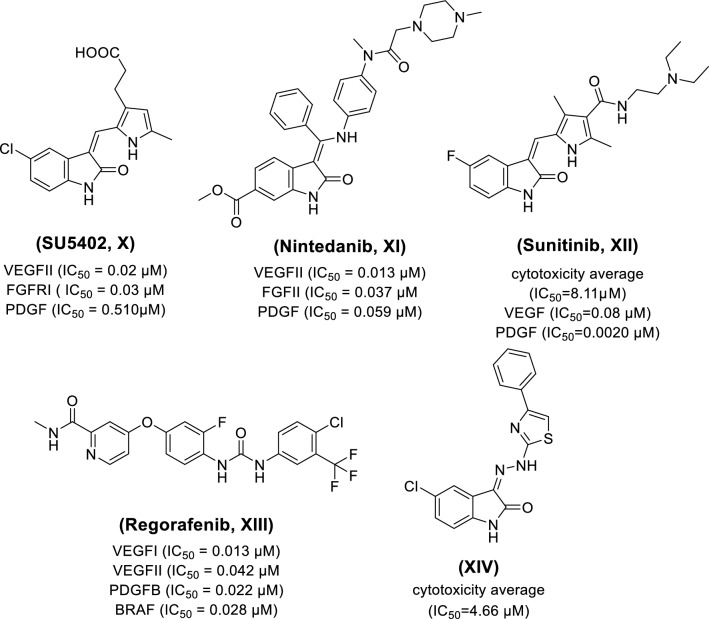
Structures of representative 2-oxindol and diphenyl urea multi-kinase inhibitors acting as anticancer drugs

On the other hand, many oxindole derivatives showed antiproliferative activity through protein kinase inhibition (e.g., compounds **X–XIV**). Nintedanib (**XI**, Ofev^®^) is a potent 3-alkenyl-oxindole multi-kinase inhibitor for VEGFRI/II/III, FGFRI/II/III and PDGFR α/β with IC_50_ of 34, 13, 13, 69, 37, 108, 59, and 65 nM, respectively [44]. It is currently in phase III clinical trials for advanced ovarian cancer treatment [45]. In the same context, Sunitinib, (**XII**, Sutent^®^), is an oxindole-based multi-tyrosine kinase inhibitor that acts on VEGFRI/II, PDGFR β and c-Kit. It was approved by the FDA in 2006 for metastatic renal cell carcinoma (RCC) and gastrointestinal stromal tumors (GIST) [46–49]. Moreover, Regorafenib **(XIII,** Stivarga ®**)** is a diphenyl urea based multi-tyrosine kinase inhibitor**.** It was FDA approved in 2012 for patients with metastatic colorectal cancer (CRC) [50], advanced GIST [51], and hepatocellular carcinoma (HCC) [52] (Fig. 2).

**Fig. 3:**
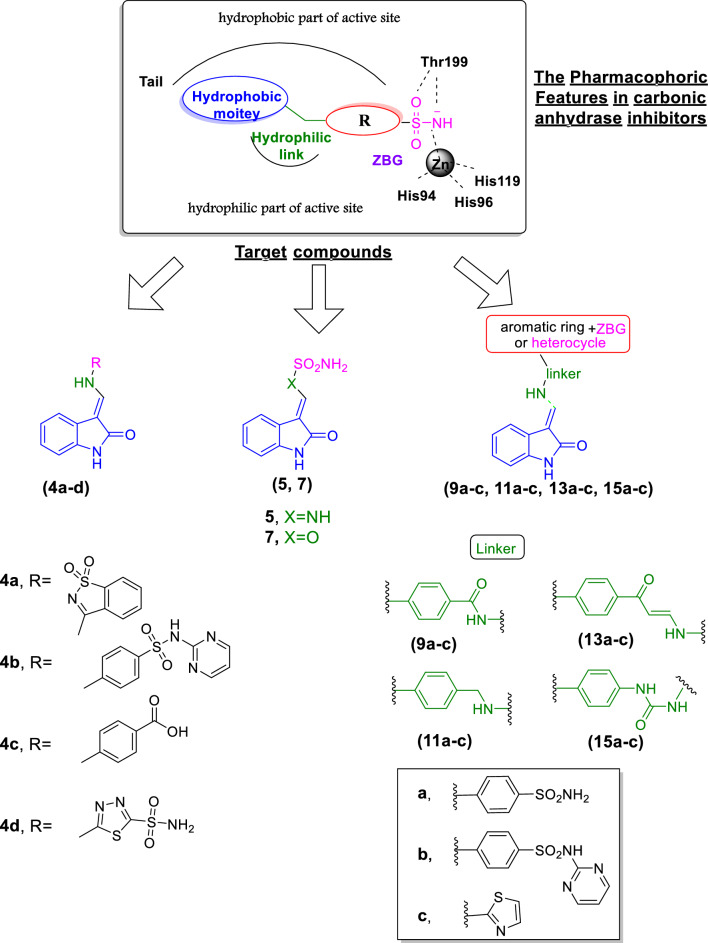
Design strategy for proposed CAIs compounds (**4a–d, 5, 7, 9a–c, 11a–c, 13a–c** and **15a–c**)

In the current research, we aimed to design and synthesize novel 3-alkenyl-oxindole derivatives as potent and selective CAIs for tumor-associated hCA isoforms knowing that indolin-2-one derivatives have a potential antiproliferative activity through different mechanisms including CA inhibition [53]. Our strategy is based on merging the essential key binding features in known CA inhibitor groups (sulfonamide, sulfonamide isosteres, or biologically active heterocycles) with diverse chemical properties and 3-alkenyl oxindole nucleus using different linkers producing three new series (**4a–d**), (**5**,** 7**), and (**9a–c**,** 11a–c**, **13a–c** and **15a–c**) (Fig. 3).

In the first series **(4a–d)**, the oxindole nucleus was linked through 3-alkenyl (methylene) to the aromatic ring or heterocycles containing different groups that are reported to have antiproliferative activity along with CA binding affinity. In the second series **(5, 7)** a simple zinc binding group (sulfamide and sulfamate) was directly attached to the 3-alkenyl oxindole scaffold. As for the third series **(9a–c, 11a–c, 13a–c, 15a–c)** different linkers with hydrogen bonding ability were used to link the 3-alkenyl oxindole scaffold to the ZBGs (primary and secondary sulfonamides or aminothiazole heterocycle), as an optimizing strategy for studying their effect on selectivity and antiproliferative activity (Fig. 3).

The newly synthesized 3-alkenyl oxindole compounds were evaluated for their ability to inhibit hCA isoforms (CA I, II, IX, and XII) and their antiproliferative activity against NCI-60 cancer cell lines. This is followed by studying the binding mode of the most potent compounds through molecular modeling.

## Results and discussion

### Chemistry

**Fig. 4:**
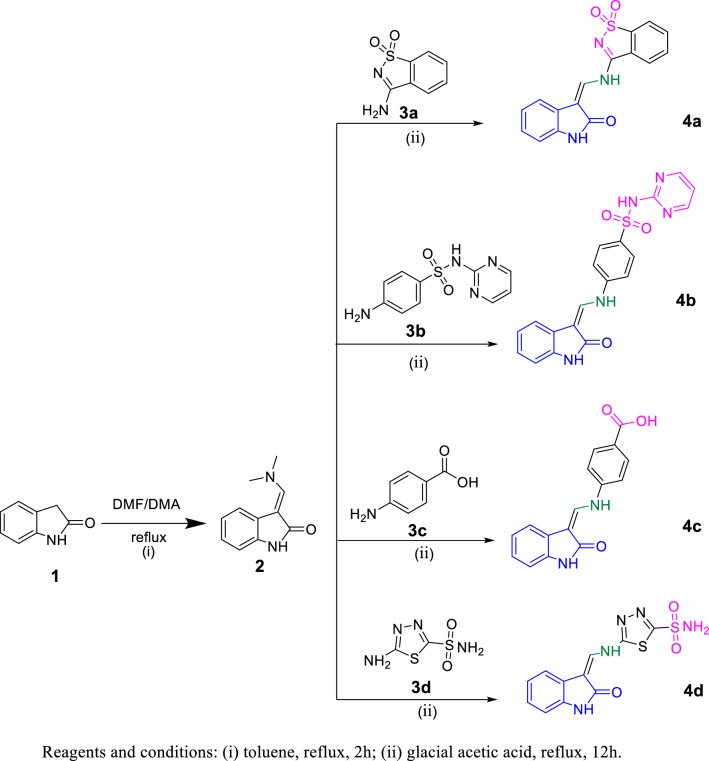
Synthesis of compounds **2** and **4a–d**

**Fig. 5:**
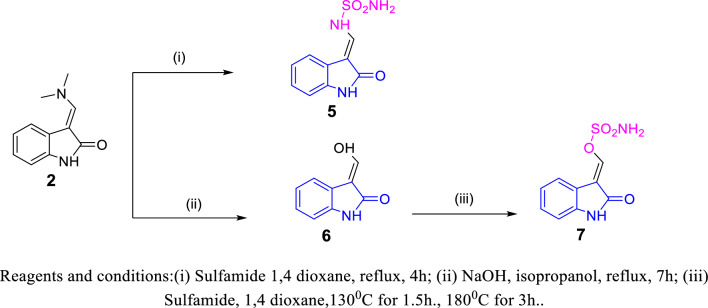
Synthesis of compounds **5**–**7**

**Fig. 6:**
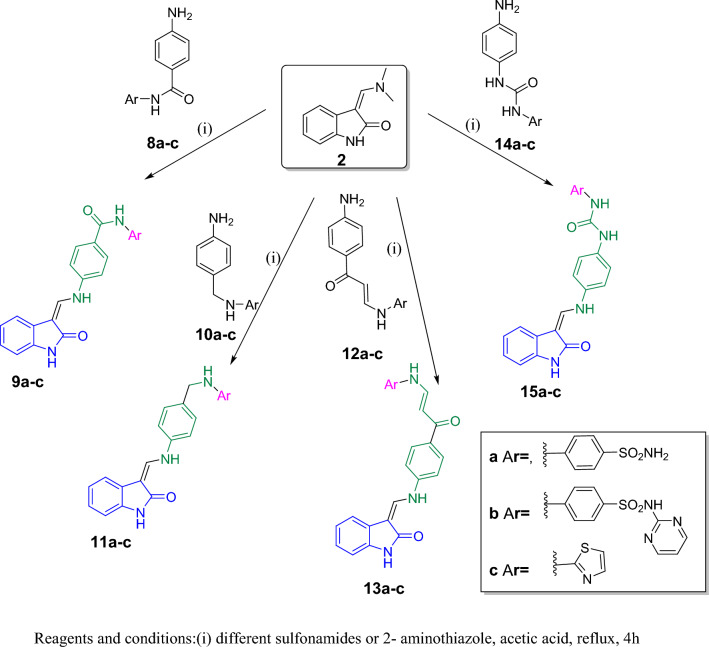
Synthesis of compounds **9a–c**, **11a–c**, **13a–c**, and **15a–c**

The synthetic pathways adopted for the preparation of the target 3-(methylene)-indol-2-ones are depicted in Figs. 4, 5, and 6. In Fig. 4, the synthesis was initiated by the condensation of the active methylene group of 2-oxindole **1** with DMF/DMA to afford the *N*-methylene intermediate **2** [54], which was reacted with different aromatic amines (**3a–d**) in glacial acetic acid to give the target compounds **4a–d**.

In Fig. 5, the 3-alkenyl oxindole derivative **5** was prepared by refluxing of the intermediate compound **2** with sulfamide in 1,4 dioxane [55]. The intermediate compound **6** could be prepared by hydrolyzing the intermediate compound **2** using NaOH. When intermediate compound **6** reacted with sulfamide in refluxing 1,4 dioxane, it afforded the sulfamate isosteric derivative **7**.

In Fig. 6, four different linker intermediates were prepared **8a–c, 10a–c, 12a–c, 14a–c** according to the reported methods [56–64] using a variety of reaction conditions then refluxed with the intermediate compound **2** in acetic acid using the general synthetic pathway adopted for the synthesis of compounds **4a–d** to afford the target compounds **9a–c, 11a–c, 13a–c, 15a–c** (Fig. 6).

The spectral data confirmed the structures of the target 3-methylene oxindoles **(4a–d**,** 5**,** 7**, **9a–c**, **11a–c**, **13a–c**, and **15a–c)**. IR spectra revealed the presence of bands of the carbonyl group at 1656–1691 cm^−1^, the amino NH and the amidic NH stretching peaks were detected between 3100 and 3404 cm^−1^. Compound **4c** showed broad band at 2500–3250 cm^−1^ corresponding to the carboxylic acid group. For compounds **4a–b**, **4d**,** 5**,** 7**, **9a–b**,** 11a–b**,** 13a–b**,** 15a–b**, the observed bands between 1331–1396 cm^−1^ and 1157–1194 cm^−1^ are assigned to the asymmetric and symmetric stretching modes of the sulfoxide group. The ^1^H NMR spectra showed protons of the 3-methylene linkage as doublet signal, around *δ* range 7.5–7.8 ppm and the oxindole protons appeared as four signals around 6.90, 7.00, 7.09*,* and 8.50 ppm*.* Compound **4a** showed two signals of saccharin protons at *δ* = 7.90–7.97 and 8.15 ppm, whereas compound **4c** showed a broad exchangeable signal at *δ* = 12.50 ppm for the carboxylic acid group. In addition, ^1^H NMR spectra of **11a–c** revealed a broad singlet signal at* δ* range 3.90–4.10 ppm due to benzylic CH_2_ protons. ^1^H NMR spectra of compounds **4d**,** 5**, **6** and compounds **15a–c** showed E/Z isomers mixtures. The presence of the E isomer was proved by the coupling constant of the olefinic hydrogens *J* = 12.0 Hz which calculated from the down-field doublet signal of E-vicinal protons at δ 7.76–7.79, and 8.12–8.30 ppm. Similarly, the presence of the Z isomer was confirmed by the coupling constant of the olefinic hydrogens with about *J* = 8 Hz which was elucidated from the up-field doublets of Z-vicinal protons at δ 5.68–6.30, 6.30–6.58 ppm with signals for vicinal protons in the range, Compounds **15a–c** showed signals that appeared in the range of 8.80–9.33 ppm assignable to the urea exchangeable protons, for compounds **4b**, **9b**,** 11b**,** 13b**, and **15b**, the pyrimidine protons appeared as two signals; the first one appeared as triplet for the proton at 4 position around 7.00 ppm and the second one appeared as a doublet for the protons at 3 and 5 positions around 8.40 ppm. On the other hand, the ^1^H NMR spectra of compounds **9c**,** 11c**,** 13c**, and** 15c** showed two signals of the thiazole protons at range 7.26–7.40 and 7.35–7.50 ppm.

The ^13^C NMR spectra also confirmed the presence of signal attributable for carbonyl group of oxindole moiety at a range of 170.10–170.38 ppm, whereas signals for the other carbonyl groups in compounds **4c** and **9a–c** appeared at a range of 165.30–167.30 ppm. Elucidation of the urea moiety in compounds **15a–c** showed signals at a range of 152.76–163.90 ppm*.* The carbonyl groups adjacent to the vinylic carbon in compounds **131a–c** were detected at the expected chemical shift at a range of 189.19–189.22 ppm. Moreover, characteristic signals appeared at a range of 95.22–96.65 ppm in this series confirming the presence of the ethylene group (–C=C–C=O).

### In vitro biological evaluation

#### In vitro antiproliferative activity assay against NCI 60-cell line panel

From the newly synthesized compounds, eleven compounds (**4c**, **4d**,** 5**, **9a**, **9b**, **11a**, **11b**, **13b**, **13c**, **15b**, and **15c**) were selected by the National Cancer Institute (NCI) Developmental Therapeutics Program (DTP), Bethesda, Maryland, USA for the in vitro anti-proliferative activity evaluation [65]. The selected compounds were examined at 10 μM dose against the NCI 60-cell line panel. This panel consists of nine cell line groups of leukemia, non-small cell lung carcinoma (NSCLC), melanoma, colon, CNS, ovarian, renal, prostate, and breast cancers. The results are reported as mean-graph of the percent growth relative to control and presented as percentage growth inhibition (GI%), furthermore, mean GI% of each compound over all panel cell lines was calculated (Table 1; Additional file 1; S3: In vitro biological activity).

**Table 1:** % Cell growth inhibition of NCI 60 cancer cell lines exhibited by investigated final compounds **(4c, 4d, 5, 9a, 9b, 11a, 11b, 13b, 13c, 15b, 15c)**

Panel/cell line	Cell Growth inhibition Percent for the tested compounds												
	**4c**	**4d**	**5**	**9a**			**9b**	**11a**	**11b**	**13b**	**13c**	**15b**	**15c**
	Leukemia												
CCRF-CEM	–	–	–		–		–	–	25.35	–	12.13	–	–
HL-60(TB)	–	–	–		–		–	–	–	0.37	–	–	57.96
K-562	–	–	–		–		3.50	0.06	32.81	2.89	24.78	2.05	82.73
MOLT-4	–	–	–		–		–	1.28	–	–	–	–	67.84
RPMI-8226	–	–	–		–		–	–	28.90	13.64	34.97	–	16.44
SR	4.7	1.71	–		–		9.02	18.74	42.22	17.85	6.00	3.55	89.33
	Non-small cell lung cancer												
A549/ATCC	9.11	–	–	–			–	4.86	–	–	21.56	–	43.44
EKVX	24.27	15.79	18.84	–			38.49	47.43	16.02	–	16.78	1.92	65.42
HOP-62	32.04	13.42	9.49	2.73			27.70	30.47	11.28	–	12.01	1.01	62.67
HOP-92	16.44	11.86	11.03	6.83			4.65	–	15.37	6.38	11.45	5.79	50.24
NCI-H226	11.4	4.95	8.23	–			12.22	19.14	16.81	–	1.09	3.30	70.05
NCI-H23	7.13	3.70	13.28	–			9.28	12.04	17.37	3.53	25.93	72.85	62.20
NCI-H322M	–	–	3.18	–			–	–	–	–	–	–	47.17
NCI-H460	–	–	–	–			1.64	–	4.26	–	7.04	–	83.16
NCI-H522	–	0.13	5.36	5.55			–	–	6.92	1.35	2.31	4.97	–
	Colon cancer												
COLO205	–	–	–	–			–	–	–	–	1.80	–	74.25
HCC-2998	–	–	–	–			–	–	–	–	–	–	26.06
HCT-116	–	–	–	–			–	–	13.39	–	21.94	7.30	73.04
HCT-15	–	–	–	–			–	–	9.43	–	12.04	2.59	70.99
HT29	–	–	–	–			–	–	–	–	8.13	–	82.12
KM12	49.21	4.63	1.51	–			43.89	13.94	77.28	62.68	59.02	49.11	40.78
SW-620	4.95	–	–	–			–	–	4.60	–	–	–	78.60
	CNS cancer												
SF-268	16.39	–	2.04	–			3.48	11.32	1.02	–	–	–	22.77
SF-295	–	0.84	–	–			–	–	0.84	–	–	–	29.41
SF-539	2.79	3.69	–	–			5.04	–	–	–	1.96	–	66.38
SNB-19	–	–	0.31	–			–	–	–	–	–	–	69.03
SNB-75	44.3	18.84	16.52	4.88			31.29	32.80	16.61	–	–	1.02	66.62
U251	–	0.06	2.06	–			–	–	14.38	–	–	–	58.05
	Melanoma												
LOX IMVI	6.93	–	–	–		7.22		3.30	8.30	–	9.06	–	64.90
MALME-3 M	0.23	–	–	–		2.09		3.16	–	–	–	–	53.15
M14	2.15	6.04	2.01	–		1.44		–	0.40	–	–	–	81.48
MDA-MB-435	–	–	–	–		–		2.04	31.96	–	1.15	–	152.70
SK-MEL-2	–	–	2.36	–		–		–	–	–	–	–	37.92
SK-MEL-28	–	–	–	–		–		–	–	–	–	–	38.68
SK-MEL-5	1.74	1.52	1.04	–		6.95		5.94	15.15	1.85	5.29	0.02	73.25
UACC-257	–	–	–	–		–		–	–	–	–	–	28.37
UACC-62	–	3.05	4.35	–		7.84		–	3.27	–	–	–	75.08
	Ovarian Cancer												
IGROV1	24.07	23.49	18.58	–			21.31	32.70	23.48	–	5.94	–	70.47
OVCAR-3	–	–	–	–			–	–	–	–	3.91	–	84.64
OVCAR-4	13.33	1.55	–	–			7.67	12.63	11.49	–	3.49	–	59.42
OVCAR-5	0.03	–	–	–			–	–	–	–	–	–	39.57
OVCAR-8	–	0.12	–	0.63			–	–	1.39	–	–	–	44.31
NCI/ADR-RES	–	–	–	–			–	–	–	–	–	–	59.19
SK-OV-3	–	0.47	5.43	–			–	–	–	–	3.75	–	44.97
	Renal cancer												
786-0	4.08	1.80	–		–		4.99	0.56	–	3.85	–	–	47.06
A498	20.07	–	–		–		9.11	–	–	1.46	–	–	107.94
ACHN	1.58	6.28	10.89		1.90		10.46	7.22	16.74	–	–	–	59.26
CAKI-1	29.12	13.50	11.2		8.65		27.60	34.72	48.43	5.62	30.80	5.21	67.07
RXF 393	29.59	–	–		–		–	26.16	–	–	–	–	120.47
SN12C	24.77	–	–		–		20.49	22.86	–	1.75	11.59	1.23	32.00
TK-10	19.11	–	–		–		–	–	–	–	–	–	24.34
UO-31	49.71	27.49	34.58		18.04		43.70	44.00	39.47	7.60	16.22	18.32	51.58
	Prostate cancer												
PC-3	7.71	10.72	5.36	–			7.10	7.36	14.22	–	15.30	5.91	31.40
DU-145	–	–	–	–			–	2.90	–	–	–	–	59.52
	Breast cancer												
MCF7	1.59	12.41	6.19	6.01			4.97	23.02	37.23	12.64	28.08	15.02	77.30
MDA-MB-231/ATCC	4.54	7.66	5.14	–			3.83	16.48	8.34	0.12	10.67	–	52.05
HS 578T	0.5	–	–	–			–	–	–	–	–	–	68.54
BT-549	–	1.21	0.96	–			27.15		6.46	–	13.62	–	
T-47D	–	14.77	7.19	–			–	6.76	27.51	6.91	–	7.81	58.82
MDA-MB-468	–	–	–	–			–	–	7.66	–	23.54	–	102.28
MEAN % Growth inhibition	3.71	− 1.47	− 1.25	− 6.41			2.55	2.58	7.38	− 3.98	3.28	− 2.47	61.83

Investigation of the primary GI% data revealed that some of the newly synthesized compounds have a promising antiproliferative activity. The most potent compound showing remarkable growth inhibition percent was compound **15c** with mean GI% of 61.83% overall the tested cell lines. Compound **15c** was a broad spectrum inhibitor for most NCI cell lines such as, Leukemic cell lines (HL-60(TB), K-562, MOLT-4, and SR) with GI% range of 57.96–89.33%, nearly all Non-small cell lung cancer with GI% ranging from 43.44 to 83.16%, colon cancer cell lines (COLO205, HCT-116, HCT-15, HT29, KM12 and SW-620) with GI% range of 40.78–82.12%, CNS cancer cell lines (SF-539, SNB-19, SNB-75, and U251) with GI% of 66.38, 69.03, 66.62, and 58.05%, respectively, melanoma cell with GI% range of 37.92–152.70%, ovarian cancer cell lines with GI% ranging from of 39.57–84.64%, renal cancer cell lines with GI% range of 32.00–120.47, %, prostate cancer cell lines (PC-3 and DU-145) with GI% of 31.40 and 59.52%, respectively, and finally, breast cancer cell lines with GI% ranging from 52.05 to 102.25%.

Moreover, compound **11b** showed potent inhibitory activity against the different NCI cell lines, namely, the leukemic (K-562 and SR) cancer cell lines with GI% of 32.81 and 42.22%, respectively. the colon cancer KM12 cell line with GI% of 77.28%, melanoma cancer MDA-MB-435 cell line with GI% of 31.96%, renal cancer cell lines (CAKI-1 and UO-31) with GI% 48.43 and 39.31%, respectively, and breast cancer cell line (MCF-7) with GI% of 37.23%.

Significant activity was also observed for compounds (**4c**, **9a**, **9b**, **11a**, and **13c**) against various cell lines such as leukemic, non-small cell lung cancer, colon, CNS, and renal cancer with growth inhibition % ranging between 30.80–59.02%. It was observed that the presence of the methylamino linkage in compounds **11a** and **11b** generally increased the antiproliferative activity over their analogues containing the amido linkage (**9a** and **9b)**. Among series **(4a–d)**, compound **4c** possessing free carboxylic acid showed inhibitory activity against NSCL (HOP-62), colon (KM12), CNS (SNB-75), renal (UO-31), and breast (BT-549) cancer cell lines ranging 32.04–49.71%. The combination between enaminone linkage and amino thiazole in compound **13c** improved the antiproliferative activity against leukemic (RPMI-8226), colon (KM12) and renal (CAKI-1) cancer cell lines with inhibition ranging 30.8–59.02%.

#### In vitro five-dose assay on selected cell lines (MCT-7, HCT-116, and DU-145)

The preliminary screening results revealed that compound **15c** showed prominent antiproliferative activity against several cell lines from the different NCI subpanels. Thus, compound **15c** was further evaluated at five-dose assay (0.02–200 µM) on the most sensitive cell lines that are available to our laboratory. Compound **15c** showed a potent growth inhibition 50% at a single digit nanomolar concentration against breast cancer (MCT-7), and prostate cancer (DU 145) cell lines, as well as a sub-nanomolar concentration against colon cancer (HCT-116) cell line with IC_50_ values of 4.39, 1.059, and 0.34 nM, respectively (Table 2; Additional file 1; S3: In vitro biological activity).

**Table 2:** In vitro cytotoxicity towards human MCT-7 (Breast), DU-145 (Prostate), and HCT-116 (Colon) cancer cell lines, expressed as mean growth inhibitory concentration 50% (IC_50_) values

Cell line target	IC_50_ (nM)
MCF-7	4.39
DU-145	1.06
HCT-116	0.34

#### Carbonic anhydrase inhibition

All the newly synthesized compounds were evaluated for their ability to inhibit the physiologically relevant cytosolic hCA isoforms, hCA I and II as well as the transmembrane tumor-associated isoforms hCA IX and XII using acetazolamide (AAZ) as a standard reference employing the stopped flow CO_2_ hydrase assay method and the CA inhibition data were presented in Table 3.

**Table 3:** Inhibition data of human CA isoforms hCA I, II, IX and XII with 3-alkenyl-oxindole derivatives **4a-d, 5, 7, 91a–c, 111a–c, 131a–c, 151a–c** and the standard inhibitor Acetazolamide (AAZ) by stopped flow CO_2_ hydrase assay

K_i_ (nM)*					Selectivity ratio			
Cmp	**hCA I**	**hCAII**	**hCA IX**	**hCA XII**	**I/IX**	**I/XII**	**II/IX**	**II/XII**
**4a**	> 10,000	8953	> 10,000	> 10,000				
**4b**	> 10,000	> 10,000	> 10,000	> 10,000				
**4c**	6323	8669	> 10,000	> 10,000				
**4d**	8.4	3.9	84.2	8.7	0.100	0.966	0.046	0.448
**5**	6345	913.5	208.2	42.4	30.48	149.65	4.39	21.54
**7**	6236	5582	940.7	133.0	6.63	46.89	5.93	41.97
**9a**	332.3	313.0	68.8	59.4	4.83	5.59	4.55	5.27
**9b**	> 10,000	> 10,000	> 10,000	> 10,000				
**9c**	> 10,000	> 10,000	> 10,000	> 10,000				
**11a**	320.8	746.4	217.4	78.6	1.48	4.08	3.43	9.50
**11b**	9108	2658	7856	8574	1.16	1.06	0.34	0.31
**11c**	> 10,000	> 10,000	> 10,000	> 10,000				
**13a**	483.0	306.6	91.3	182.7	5.29	2.64	3.36	1.68
**13b**	> 10,000	> 10,000	> 10,000	> 10,000				
**13c**	> 10,000	> 10,000	> 10,000	> 10,000				
**15a**	844.5	762.5	863.3	175.0	0.98	4.83	0.88	4.36
**15b**	> 10,000	> 10,000	> 10,000	> 10,000				
**15c**	> 10,000	> 10,000	> 10,000	> 10,000				
**AAZ**	250.0	12.1	25.8	5.7	9.69	43.86	0.47	2.12

* Mean from 3 different assays, by a stopped flow technique (errors were in the range of ± 5–10% of the reported values)

Regarding the inhibition of the cytosolic isoform hCA I, it was observed that the tested compounds revealed Ki values ranged between 8.4 and > 10,000 nM. Compound **4d** was the most potent inhibitor in the series with Ki of 8.4 nM, whereas compounds **9a**, **11a**, **13a**, and **15a** displayed moderate activities with Ki values of 332.3, 320.8, 483.0, and 844.5 nM, respectively, compared to the used reference standard AAZ (Ki = 250 nM). Compounds **4c**, **5**, **7**, and **11b** showed weak inhibitory activity with Ki values of 6323, 6345, 6236, and 9108 nM, respectively. Whereas the rest of the compounds showed no hCA I inhibitory activity with Ki values higher than 10,000 nM. The results showed that the compounds containing *N*^1^ unsubstituted benzenesulfonamide moiety have higher inhibitory activity than that of the substituted or cyclic analogues. Furthermore, the carboxylic acid bioisostere e.g., compound **4c** showed lower activity than the benzenesulfonamide derivatives. Additionally, direct attachment of the sulfamate and sulfamide moieties to the 3-alkenyl-indolin-2-one scaffold led to a significant decrease in the inhibitory effect, as can be noticed in compounds **5** and **7**. The spacer diversity (amido, methyl amino, enaminone, and ureido) have no obvious effect on the inhibitory activity.

As can be seen in Table 2, the tested compounds showed nearly the same inhibitory activities, activity pattern, and SAR towards hCA II isoform as that are shown towards hCA I with Ki values ranging between 3.9 and > 10,000 nM.

Concerning the tumor-associated hCA IX inhibition results, it was found that the tested compounds exhibit Ki values ranging between 68.8 and > 10,000 nM. Compound **9a** with primary sulfonamide and amido spacer showed the most potent hCA IX inhibition with Ki value of 68.8 nM and selectivity ratio of 4.83 and 4.55 over hCA I and hCA II, respectively, with the used standard (AAZ) showing Ki of 25.80 nM and selectivity ratio of 9.69 and 0.47 over hCA I and hCA II, respectively. Moreover, compound **13a** showed a potent hCA IX inhibition with Ki value of 91.3 nM and selectivity ratio of 5.29 and 3.36 over hCA I and hCA II, respectively. On the other hand, despite its potent hCA IX inhibition with Ki of 84.2 nM, compound **4d** showed higher selectivity towards hCA I and hCA II over hCA IX with selectivity ratios of 0.10 and 0.046, respectively. Compounds** 5** and **7,** with ZBGs directly attached to the 3-methylene oxindole scaffold, showed moderate inhibitory activity towards hCA IX with Ki values of 208.2 and 940.7 nM, respectively, which is lower than that of hCA I and hCA II giving selectivity ratios of 30.48 and 4.39, respectively, for compound **5**, and 6.63 and 5.93, respectively, for compound **7**. As for compounds **11a**, **11b**, and **15a** (hCA IX Ki values of 217.4, 7856, and 863.3 nM, respectively), they showed the same level of activity towards physiological hCA I and/or hCA II and tumor-associated hCA IX with no sign of selectivity towards neither of them. Whereas the rest of the compounds showed no hCA IX inhibitory activity with Ki values higher than 10,000 nM.

Against the tumor-associated hCA XII, the tested compounds showed inhibition constant Ki range between 8.7 and > 10,000 nM. Compounds **5**, **9a**, and **11a** exerted a potent inhibition towards hCA XII with Ki value of 42.4, 59.4, and 78.6 nM, respectively, showing reasonable selectivity ratios of 149.65, 5.59, and 4.08, respectively, over hCA I and of 21.54, 5.27 and 9.50, respectively, over hCA II compared to the reference compound acetazolamide with Ki of 5.7 nM and selectivity ratios of 43.86 and 2.12 over hCA I and II, respectively. As noted, the presence of the amido and (CH_2_–NH) linkers along with the primary sulfonamide group in compounds **9a** and **11a** resulted in a promising tumor-associated hCA XII inhibition profile. Compounds **7**, **13a** and **15a** showed moderate activities with Ki values of 133, 182.7 and 175 nM, respectively, showing reasonable selectivity ratios of 46.89, 2.64, and 4.83, respectively, over hCA I and of 41.97, 1.68 and 4.36, respectively, over hCA II. Despite its potent inhibitory activity (Ki = 8.7 nM), compound **4d** was less selective towards hCA XII in comparison to hCA I and II (selectivity ratios = 0.966 and 0.448, respectively). On the other hand, compound **11b** showed both low potency (Ki = 8574 nM) and selectivity ratios towards hCA XII over hCA I and II (1.06 and 0.31, respectively). Whereas the rest of the compounds showed no hCA XII inhibitory activity with Ki values higher than 10,000 nM.

It could be concluded from these findings that CA inhibition activity showed less selectivity towards hCA IX which could mean that the antiproliferative action of the synthesized compounds is independent from the CA activity.

#### In vitro tyrosine kinase activity

Despite its weak CA inhibitory activity, compound **15c** showed a remarkable antiproliferative activity against a wide range of cell lines. These results indicate that compound **15c** exerts its cytotoxic activity through another mechanism of action.

The activation of multiple signaling pathways in the tumor microenvironment associated with the dysfunction of protein kinase activity specially receptor tyrosine kinases (RTKs) such as RET, Kit, VEGFR-1, VEGFR-2, FGFR1, PDGFR, BRAF, and c-Met [3, 66, 67]. These TKs play an important role in the development and progression of multiple cancers. Moreover, some of these TKs such as vascular endothelial growth factor receptor (VEGFR), fibroblast growth factor receptor (FGFR) and platelet-derived growth factor receptor (PDGFR) play crucial roles in the biology of normal and tumor vasculature as well as neo-angiogenesis (angiokinases) which is vital for the survival and proliferation of tumor cells [68]. Furthermore, VEGFR is involved in many downstream pathways, such as PI3K, p38 MAPK, FAK, Src, and Akt that are sometimes overexpressed in several tumors including ovarian, renal, melanoma playing a vital role in neoplasm metastasis [69]. Therefore, inhibition of cancer angiogenesis and the blockage of multiple growth factors could be of great interest to increase the efficacy of cancer therapy.

**Fig. 7:**
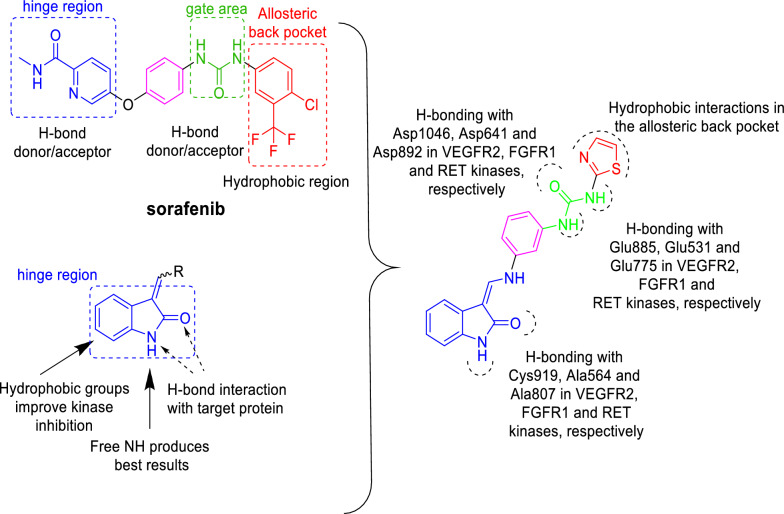
Structural insights of compound **15c**, showing the main pharmacophoric features reported for the multi-kinase inhibition activity of sorafenib and oxindole-based inhibitors

The promising activity of compound **15c** could be attributed to its kinase inhibition activity. Interestingly, this compound comprises the main pharmacophoric features reported for the multi-kinase inhibition profile as in the clinically approved multi-kinase inhibitor, sorafenib [70] (Fig. 7). Thus, the kinase inhibitory activity for compound **15c** was evaluated against different protein kinases **(**RET, Kit, c-Met, VEGFR-1, VEGFR-2, FGFR1, PDGFR and BRAF) to validate this hypothesis.

##### Initial screening at a single dose of 10 μM concentration.

To test the potential kinase inhibitory activity of compound **15c**, initial single dose testing was performed at 10 μM on a panel of kinases and their inhibition % were determined. The assays were performed at Thermo Fischer Scientific, USA (www.thermofischer.com/selectscreen) against RET, Kit, c-Met, VEGFR-1, VEGFR-2, FGFR1, PDGFR and BRAF using staurosporine as a reference compound (Additional file 1; S3: In vitro biological activity).

Interestingly, compound **15c** showed promising activity against most of the tested kinases with a percent inhibition range of 31–74% and with a higher potency towards the tumor-associated VEGFR-2 over VEGFR-1 [71] (Table 4).

**Table 4:**
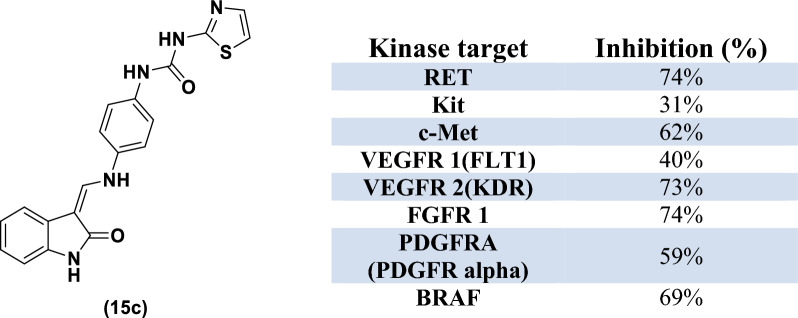
Percent inhibition at 10 uM against RET, Kit, c-Met, VEGFR-1, VEGFR-2, FGFR1, PDGFR and BRAF achieved by compound **15c**.

##### Measurement of potential enzyme inhibitory activity (IC_50_)

The promising candidate **15c** demonstrated an inhibition percentage above 70% against FGFR1, RET and VEGFR-2 kinases at 10 μM concentration that prompted us to further examine its dose-related enzymatic inhibition at five different concentrations (10 nM–100 nM–1 μM–10 μM–100 μM) to determine its IC_50_ values against these kinases. The 3-methylene oxindole derivative **15c** displayed a potent multi-kinase inhibitory activity against FGFR1, VEGFR-2 and RET kinases with IC_50_ values of 1.287, 0.117 and 1.185 μM, respectively. These significant activities could be related to the combination of certain privilege scaffolds such as ureido and thiazole moieties (Table 5; Additional file 1; S3: In vitro biological activity).

**Table 5:** IC_50_ values against FGFR1, VEGFR-2 and RET achieved by the most active candidate **15c**

Kinase target	IC_50_ (μM)
FGFR 1	1.287
VEGFR 2(KDR)	0.117
RET	1.185

### Molecular modeling study

#### Molecular docking in carbonic anhydrase isozymes (CA II, CA IX and CA XII)

The most potent compounds (**4d**, **5**, and **7**) were docked into the active site of hCA II, hCA IX and hCA XII isoforms to investigate their binding pattern. To this end, the protein structure of hCA II (PDB ID: 3HS4) [72], hCA IX (PDB ID: 5FL4) [73], and hCA XII (PDB ID: 1JD0) [74] isozymes were retrieved from the protein data bank. The Molecular docking protocol was initially validated by self-docking of the complexed inhibitor in proximity of the active site of each isozyme. The validation step showed the aptness of the used docking protocol for the intended molecular docking study as evidenced by the small RMSD values (1.460 Å, 1.095 Å, and 1.558 Å in CA II, CA IX and CA XII, respectively) and by the capability of the complexed inhibitors docking poses to regenerate all the important interactions achieved by the complexed inhibitors with the key residues in CA II, CA IX, and CA XII active sites (Zn^2+^, Thr199 and/or Thr200) (For further details, see Additional file 1; S2: Molecular docking study). Then, the binding pattern of compounds (**4d**, **5**, and **7**) in the active sites of the target carbonic anhydrase isozymes was investigated using the validated molecular docking protocol.

**Fig. 8:**
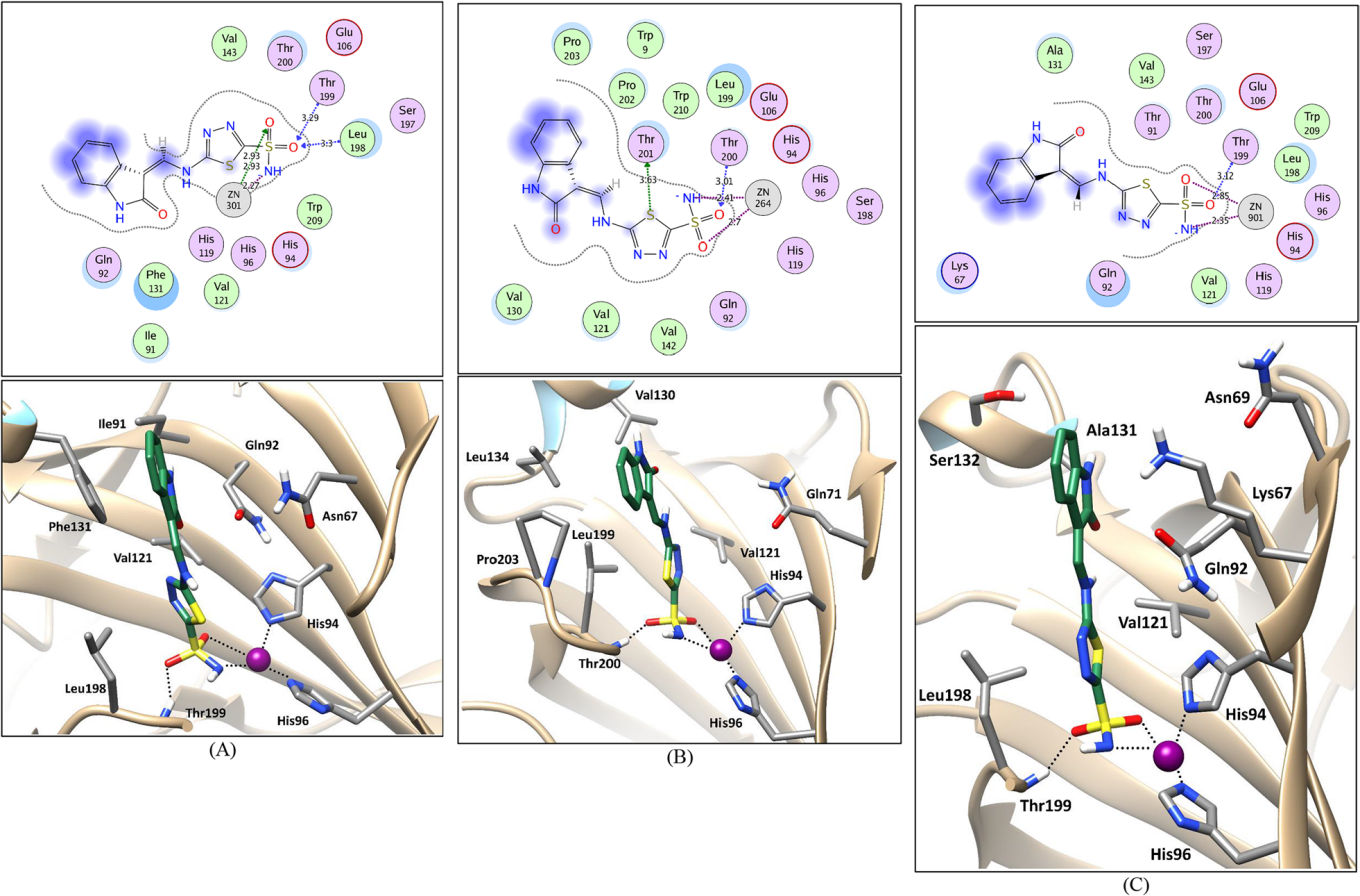
2D interaction diagrams and 3D representations showing compound **4d** docking pose interactions with the key amino acids in the CA II (**A**), CA IX (**B**), and CA XII (**C**) active sites. (Distances in Å)

The molecular docking study indicated that the binding pattern of the tested compounds with the three CA isoforms involves the sulfamoyl moiety fitting in the active site via the coordination with the Zn^2+^ cation and H-bond with the main residues Thr199 and/or Thr200. Moreover, the thiadizole ring in compound **4d** and the indolinone ring in compounds **5** and **7** are engaged in hydrophobic interactions with the hydrophobic side chains of the residues Val121 in the three isozymes, Leu198 in CA II and CA XII, and Leu199, and Pro203 in CA IX (Fig. 8 and for further details see Additional file 1; S2: Molecular docking study). In compound **4d**, the distal indolinone ring interacting further through hydrophobic interactions with the side chains of the residues Ile91 and Phe131, Val130 and Val134, and Ala131in CA II, CA IX, and CA XII, respectively, (Fig. 8) what rationalizing its superior inhibitory activity as indicating by its experimental results (Table 2) and docking scores in comparison to the used reference standard **AAZ** (Table 6). As can be noticed in Fig. 8 and Additional file 1, the NH^−^–Zn^2+^ distances are in the range of 2.23–2.41 Å which is slightly higher than that of the crystal structures (1.94–2.07 Å) which could be attributed to the nature of the performed rigid-protein docking which tries to fit the whole molecule in the rigid active site on the expense of the pivotal Zn^2+^ coordinate bond geometry which is the most relevant to the CA enzyme inhibition.

**Table 6:** Docking energy scores (*S*) in kcal/mol for compounds **4d**, **5**, and **7** and the reference AAZ in CA II, CA IX and CA XII active sites

Compound		(*S*) in kcal/mol	
	CA II	CAIX	CA XII
**4d**	− 9.95	− 8.92	− 9.09
**5**	− 9.24	− 8.02	− 8.93
**7**	− 9.00	− 8.66	− 9.16
**AAZ**	− 9.59	− 9.17	− 7.74

#### Molecular docking in the protein kinases (VEGFR-2, FGFR1 and RET)

Molecular docking simulations were also carried out to study the binding mode of compound **15c** in the active site of the kinases VEGFR-2, FGFR1, and RET and to rationalize its inhibitory activity.

The diaryl urea structure of compound **15c** proposes its probable type II-like PTK inhibitory binding mode which involves the occupation of the hinge region, the gate area, and the extension further than the gatekeeper into the allosteric back pocket at the kinase domain. So, in the present simulations, the used VEGFR-2 (PDB ID: 4ASD [75]) and FGFR-1 (PDB ID:4V01 [76]) protein structures are in complex with a type II PTK inhibitor, sorafenib and ponatinib, respectively, adapting a DFG-out conformation with the three key binding regions open and set for binding. As for RET kinase, a DFG-out conformation bound to a type II kinase inhibitor (sorafenib) was constructed using PDB ID: 6NEC [77] (vide infra in the experimental).

Initially, docking protocol was validated by performing self-docking of the complexed kinase inhibitors in the proximity of the VEGFR-2, RET, and FGFR-1 kinase domain. The self-docking validation step regenerated the interaction pattern of the complexed inhibitors accurately indicating that the adopted docking setup is appropriate for the proposed docking study. This is shown by the low RMSD between the complexed inhibitors and their docking poses (0.470 Å, 0.398 Å, and 0.331 Å in PDB IDs 4ASD, 4V01, and 6NEC, respectively). Moreover, by the capability of the resulted poses to regenerate all the main interactions achieved by the complexed inhibitors with the key residues at the kinase domain; Glu885, Cys919 and Asp1046 (in VEGFR-2), Glu531, Ala564 and Asp641 (in FGFR1), and Glu775, Ala807 and Asp892 (in RET) kinases (Table 7) and (Additional file 1; S2: Molecular docking study; Figures S1–S3).

**Table 7:** Docking energy scores (*S*) in kcal/mol for compound **15c** and the co-crystalized ligands in VEGFR-2, FGFR1 and RET active sites

Compound		(*S*) in kcal/mol	
	VEGFR-2	FGFR1	RET
**15c**	− 12.94	− 13.06	− 13.04
**Co-crystalized** **ligand**	− 15.19	− 17.00	− 14.23

**Fig. 9:**
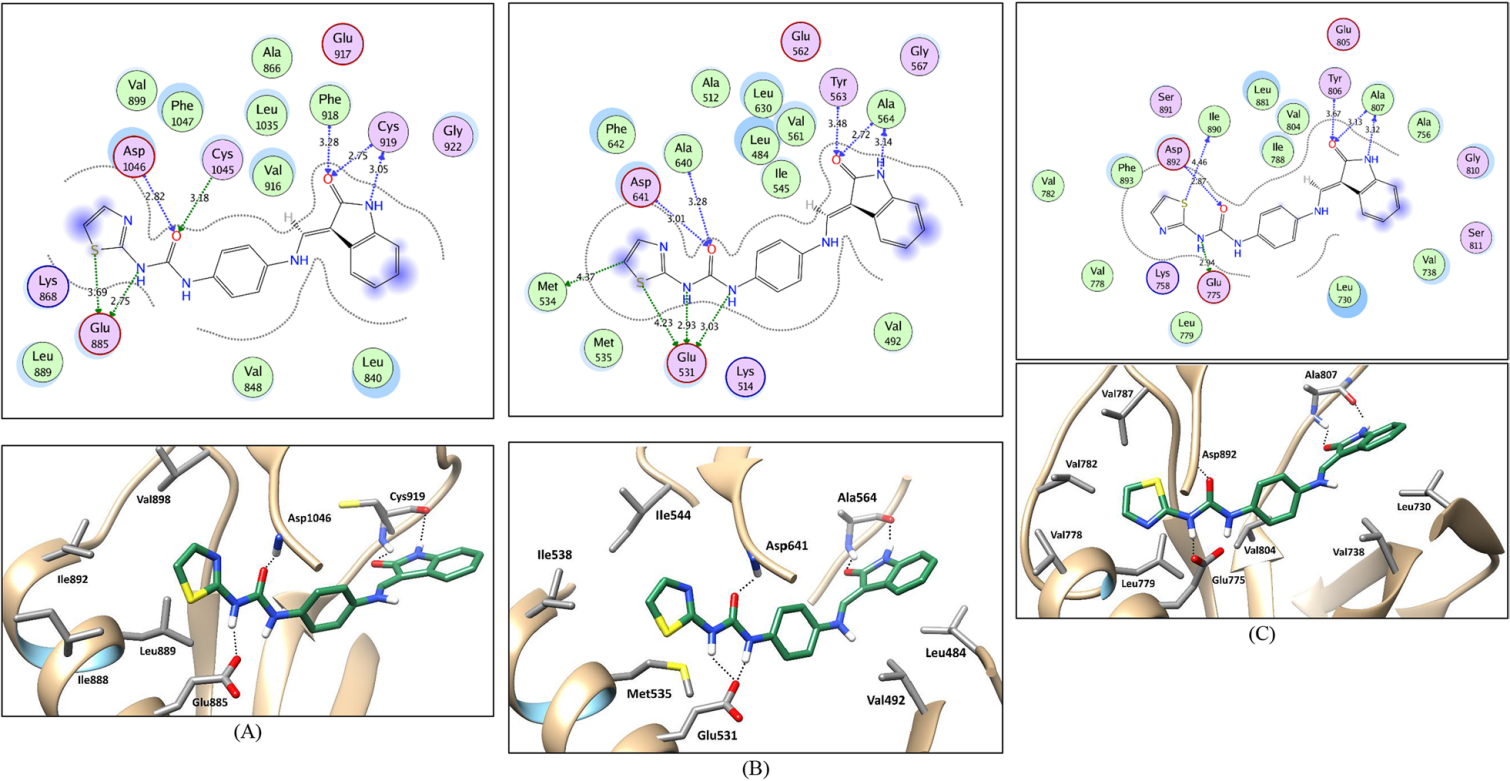
2D interaction diagrams and 3D representations showing compound **15c** docking pose interactions with the key amino acids in the VEGFR-2 (**A**), FGFR1 (**B**), and RET (**C**) active sites. (Distances in Å)

Compound **15c** showed a comparable binding mode in the three target kinases that agrees with that of type II kinase inhibitors (Fig. 9). In the central gate area, the phenyl uriedo moiety interacts through cation-π and hydrophobic interactions by their phenyl moiety with the side chains of the gate area amino acids Val848, Val916, Cys1045 and Lys868 in VEGFR-2, Ile545, Val561, Ala640 and Lys514 in FGFR-1, and Ile788, Val804, Leu881, and Lys758 in RET and through hydrogen bonding by their urea moiety with Asp1046, Asp641, and Asp892 of the conserved DFG motif in VEGFR-2, FGFR-1, and RET, respectively, and with the side chain carboxylate of Glu885, Glu531, and Glu775 of the αC helix in VEGFR-2, FGFR-1, and RET, respectively. This binding mode directs from one side the indolinone moiety towards the front pocket (hinge region) interacting through hydrogen bonding with Cys919, Ala564, and Ala807 in VEGFR-2, FGFR-1, and RET, respectively, and through hydrophobic interaction with the hydrophobic side chains of the amino acids Leu840, Phe918, Cys919, Leu1035 and Phe1047 in VEGFR-2, Leu484, Val492, Ala564, Leu630 and Phe642 in FGFR-1, and Leu730, Val738, Ala756, and Ala807 in RET. On the other side, it directs the thiazole moiety towards the allosteric back pocket interacting through hydrophobic interaction with the hydrophobic side chains of its lining residues Ile888, Leu889, Ile892, Val898, Val899, Leu1019 and Ile1044 in VEGFR-2, Met534, Met535, Ile538, Ile544, Leu614, Cys619 and Ile639 in FGFR-1, and Val778, Leu779, Val782, Ile890, and Phe893 in RET kinases (Fig. 9).

## Conclusion

To target carbonic anhydrase as antiproliferative therapy, diverse molecular structure modifications on 3-alkenyl indolin-2-one scaffold were designed with consideration of keeping the essential pharmacophoric features for carbonic anhydrase inhibition. Optimization strategies such as bio-isosteric replacement (**4b-d, 5** and** 7**), ring fusion (**4a**) and extension (**91a–c**, **111a–c**, **131a–c** and **151a–c**) were adopted.

The newly designed series 3-alkenyl indolin-2-one were synthesized and evaluated for their in vitro carbonic anhydrase inhibitory activity on hCA isoforms I, II, IX and XII as well as anti-proliferative activity against NCI sixty cell lines panel.

Compound **15c** showed no carbonic anhydrase inhibitory activity, however, it showed distinct potent and broad antiproliferative activity with mean growth inhibition 61.83% and with IC_50_ values of 4.39, 1.06, and 0.34 nM, respectively against MCT-7, DU 145, and HCT-116 cell lines. The activity was then explained through other suggested mechanisms from literature and from getting more insight into the pharmacophoric features affecting its antiproliferative activity.

Compound **15c** was evaluated against eight tyrosine kinases (RET, Kit, c-Met, VEGFR-1, VEGFR-2, FGFR, PDGFR and BRAF) to explore its potential multi-kinase targeting efficacy towards the whole cascade of tumorigenesis. The newly synthesized 3-alkenyl indolin-2-one derivative bearing aryl and thiazole urea tail via an NH linker showed the most active inhibition activity against FGFR, VEGFR-2 and RET kinases showing IC_50_ values of 1.28, 0.117 and 1.18 μM, respectively. These results were studied further using molecular docking studies, which demonstrated the capability of compound **15c** to achieve the essential interactions, known to be crucial for the inhibition of FGFR, VEGFR-2 and RET kinases with additional H bond and hydrophobic interactions.

In conclusion, compound **15c** could be a promising multi-kinase inhibitory agent and this clearly justifies its potent efficacy towards different cancer cell lines.

## Experimental

### Chemistry

#### General remarks

Starting materials, reagents and solvents were obtained from commercial suppliers and used without further purification. All the reactions were monitored by thin layer chromatography silica gel F 254, Aluminum sheets 20 × 20 cm (Sigma-Aldrich) were used. Dichloromethane: methanol (1: 0.1) was the adopted elution system. Compounds **2** [54], **3a** [78, 79], **3d** [80], **5, 6** [81]**, 8(1a–c), 10(1a–c), 12(1a–c)** and **14(1a–c)** [56–64] were synthesized according to reported procedures. Further general remarks related to the chemistry experimental are reported in the Additional file 1; S1: spectral data.

#### General procedure for preparation of target compounds (4a–d)

The appropriate amine **(3a-–)** (2.67 mmol) was added to a solution of **(2)** (2.67 mmol, 0.5 g) in acetic acid (5 mL) and the reaction mixture was heated under reflux for 12 h, the acetic acid was evaporated under vacuum and the residue was washed with diethyl ether, recrystallized from EtOAc and hexane.

#### 3-{[(1,1-Dioxidobenzo[d]isothiazol-3-yl)amino]methylene}indolin-2-one (4a)

Yield 90%, mp 195–199 °C. IR **(KBr, *ύ* cm**^**−1**^**)**: 3150–3383 (2NH), 3083 (CH aromatic), 1691 (CO), 1606 (NH bending), 1550 (C=C aromatic), 1159, 1361 (SO_2_)**; **^**1**^**H NMR (DMSO-d6, 400 MHz)** δ ppm: 6.92 (d, 1H, oxindole-H7, *J* = 7.6 Hz*)*, 6.99 (t, 1H, oxindole-H5, *J* = *8.4* Hz), 7.19 (t, 1H, oxindole-H6, *J* = 7.6 Hz), 7.82 (d, 1H, –C=CH–, *J* = 7.2 Hz), 7.9–7.97 (m, 3H, saccharine-H5,H6,H7), 8.15 (d, 1H, saccharine-H4, *J* = 7.2 Hz), 8.52 (brs, 1H, oxindole-H4), 11.07 (s, 1H, oxindole–NH_,_ D_2_O exchangeable), 12.2 (s, 1H, –C=CH–NH_,_ D_2_O exchangeable); ^**13**^**C NMR (DMSO-d6)** δ**:** 110.6, 112.8, 121.5, 122.2, 122.6, 123.6, 128.4, 131.1, 133.5, 134.00, 134.4, 140.3, 141.9, 142.7, 161.3, 170.7; **MS**: (Mwt.: 325.34): *m/z* (% rel. Int.), 325.40 (M^+^, 5.95%), 326.22 (M^+^ + 1, 4.93%), 144.23 (100%); **Anal.** Calcd. for C_16_H_11_N_3_O_3_S: C, 59.07; H, 3.41; N, 12.92; S, 9.85; Found: C, 59.24; H, 3.67; N, 13.09; S, 9.97.

#### 4-{[(2-Oxoindolin-3-ylidene)methyl]amino}-N-(pyrimidin-2-yl)benzenesulfonamide (4b)

Yield 83%, m.p 238–240 °C. **IR (KBr, *ύ* cm**^**−1**^**):** 3151–3366 (3NH), 3055 (CH aromatic), 1671 (CO), 1618 (NH bending), 1521–1575 (C=C aromatic), 1177, 1371 (SO_2_)**; **^**1**^**H NMR (DMSO-d6, 400 MHz)** δ ppm: 6.83 (d, 1H, oxindole-H7, *J* = 8 Hz*)*, 6.9 (t, 1H, oxindole-H5, *J* = *8* Hz), 6.98–7.09 (m, 2H, oxindole-H6, pyrimidine-H5), 7.5 (d, 2H, Ar–H, *J* = 8.4 Hz), 7.57 (d, 1H, –C=CH–, *J* = *8.*4 Hz), 7.91 (d, 2H, Ar–H, *J* = 8.8 Hz), 8.45 (dd, 2H, pyrimidine-H2, H4, *J* = 8.8 Hz), 8.56 (d, 1H, oxindole-H4, *J* = 12 Hz), 10.55 (s, 1H, oxindole-NH_,_ D_2_O exchangeable), 10.8 (d, 1H, –C=CH–NH_,_ D_2_O exchangeable, *J* = 12 Hz), 11.5 (sbr, 1H, SO_2_NH_,_ D_2_O exchangeable); ^**13**^**C NMR (DMSO-d6)** δ**:** 102.4, 109.80, 115.7, 118.0, 121.0, 124.1, 125.3, 129.9, 130.0, 130.2, 133.9, 136.9, 144.20, 153.4, 157.5, 158.7, 170.2; **MS**: (Mwt.: 393.42): *m/z* (% rel. Int.), 393.55 (M^+^, 3.48%), 394.75 (M^+^ + 1, 3.44%), 45.26 (100%); **Anal.** Calcd. for C_19_H_15_N_5_O_3_S: C, 58.01; H, 3.84; N, 17.80; S, 8.15; Found: C, 58.21; H, 4.06; N, 17.98; S, 8.27.

#### 4-{[(2-Oxoindolin-3-ylidene)methyl]amino}benzoic acid (4c)

Yield 92%, m.p > 300 °C, IR **(KBr, *ύ* cm**^**−1**^**)**: 2500–3300 broad band (COOH, 2NH), 1569, 1667 (2CO); ^**1**^**H NMR (DMSO-d6, 400 MHz)** δ ppm: 6.86 (d, 1H, oxindole-H7, *J* = 8 Hz*)*, 6.93 (t, 1H, oxindole-H5,* J* = 8 Hz), 7.03 (t, 1H, oxindole-H6, *J* = 8 Hz), 7.46 (d, 2H, Ar–H,* J* = 8 Hz), 7.62 (d, 1H, –C=CH–, *J* = 8 Hz), 7.93 (d, 2H, Ar–H,* J* = 8 Hz), 8.63 (d, 1H, oxindole-H4,* J* = 12 Hz), 10.58 (s, 1H, oxindole-NH_,_ D_2_O exchangeable), 10.85 (d, 1H, –C=CH–NH_,_ D_2_O exchangeable, *J* = 12 Hz), 12.53 (s, 1H, –COOH_,_ D_2_O exchangeable); ^**13**^**C NMR (DMSO-d6)** δ**:** 102.0, 109.8, 115.7, 116.4, 118.0, 121.0, 124.3, 125.2, 131.5, 137.1, 137.9, 144.2, 167.3, 170.3; **MS**: (Mwt.: 280.28): *m/z* (% rel. Int.), 280.34 (M^+^, 20.34%), 381.35 (M^+^ + 1, 3.81%), 144.25 (100%); **Anal.** Calcd. for C_16_H_12_N_2_O_3_: C, 68.56; H, 4.32; N, 9.99; Found: C, 68.34; H, 4.50; N, 10.21.

#### 5-{[(2-Oxoindolin-3-ylidene)methyl]amino}-1,3,4-thiadiazole-2-sulfonamide (4d)

Yield 66%, mp 210–216 °C, **Z/E mixture 3:1**. IR **(KBr, *ύ* cm**^**−1**^**)**: 3144, 3270, 3381 (2NH, NH_2_), 3050 (CH aromatic), 1680 (CO), 1615 (NH bending), 1529 (C=C aromatic), 1170, 1336 (SO_2_); ^**1**^**H NMR (DMSO-d6, 400 MHz) δ ppm**: 6.85–6.89, 7.59–7.61 (m, 1H, oxindole-H7*)*, 6.93–6.98, 7.63–7.65 (m, 1H, oxindole-H5, *J* = 7.2 Hz), 7.1–7.18, 7.71–7.74 (m, 1H, oxindole-H6), 8.06–8.1, 8.23 (m, 1H, –C=CH–), 8.34–8.43 (m, 2H, SO_2_NH_2_, D_2_O exchangeable), 8.66 (brs, 1H, oxindole-H4), 10.44 (s, 1H, NH_,_ D_2_O exchangeable), 10.68 (s, 1H, NH_,_ D_2_O exchangeable); ^**13**^**C NMR (DMSO-d6)** δ**:** 106.8, 108.0, 109.7, 119.1, 121.2, 127.1, 132.9, 139.4, 166.6, 169.2, 172.1; **MS**: (Mwt.: 323.35): *m/z* (% rel. Int.), 323.85 (M^+^, 3.47%), 144.27 (100%); **Anal.** Calcd. for C_11_H_9_N_5_O_3_S_2_: C, 40.86; H, 2.81; N, 21.66; S, 19.83; Found: C, 40.08; H, 2.94; N, 21.88; S, 19.75.

#### 3-(sulfamidmethylene)indoline-2-one (5)

A mixture of **(2)** (4.15 mmol, 0.78 g) and sulfamide (26 mmol, 2.4 g) in 1,4-dioxane was heated to reflux for 4 h. Then, the reaction was cooled and diluted with water, extracted with chloroform, dried over MgSO_4_ and evaporated under vacuo, the residue was recrystallized from n-butanol to give compound **(5).**

Yield 40%, m.p: 158–160 °C, **Z/E mixture 1:1**. IR **(KBr, *ύ* cm**^**−1**^**)**: 3116–3250 broad band (NH, NH_2_), 3051 (CH aromatic), 1684 (CO), 1677 (NH bending), 1514 (C=C aromatic), 1178, 1374 (SO_2_))**; **^**1**^**H NMR (DMSO-d6, 400 MHz)** δ ppm: 6.87–6.97 (m, 2H, oxindole-H7, H5*)*, 7.08–7.16 (m, 1H, oxindole-H6), 7.39–7.46 (m, 2H, –NHSO_2_NH_2_, D_2_O exchangeable), 7.61, 7.69 (2d, 1H, –C=CH–, *J* = *8.4* Hz), 7.77, 8.04 (2*s*, 1H, NH_2_SO_2_NH-_,_ D_2_O exchangeable), 8.19, 8.46 (brs, s, 1H, oxindole-H4), 10.4, 10.56 (2*s*, 1H, oxindole-NH_,_ D_2_O exchangeable); ^**13**^**C NMR (DMSO-d6)** δ**: **112.6, 114.30, 118.6, 129.3, 130.9, 132.5, 143.4, 158.7, 169.4; **MS**: (Mwt.: 239.25): *m/z* (% rel. Int.), 239.58 (M^+^, 8.02%), 144.28 (100%); **Anal.** Calcd. for C_9_H_9_N_3_O_3_S: C, 45.18; H, 3.79; N, 17.56; S, 13.40; Found: C, 45.43; H, 3.88; N, 17.82; S, 13.51.

#### (2-Oxoindolin-3-ylidene)methyl sulfamate (7)

Mixture of (0.1 mol, 16.1 mg) of **(6)** and (0.25 mol, 24 mg) of sulfamide in 1,4-dioxane was heated with stirring in an oil bath at 130 °C for 1 h, then excess of (0.25 mol, 24 mg) sulfamide was added, heating was continued at 130 °C for 0.5 h, and then the temperature was raised slowly to 180 °C. After heating for additional 3 h with stirring, the mixture was allowed to cool and was stirred with a mixture of 150 ml of water and 150 ml of methylene chloride. The organic layer was separated, and the solvent was removed, the residue was recrystallized from methanol giving compound **7**.

Yield 60%, m.p: 165–170 °C, **Z/E mixture 2:1**. IR **(KBr, *ύ* cm**^**−1**^**)**: 3133–3333 broad band (NH, NH_2_), 3063 (CH aromatic), 1669 (CO), 1615 (NH bending), 1517 (C=C aromatic), 1192, 1328 (SO_2_ of sulfamate); ^**1**^**H NMR (DMSO-d6, 400 MHz) δ ppm**: 6.17, 6.40 (2*s*, 1H, –C=CH–), 6.87–6.94 (m, 2H, oxindole-H7,H5), 7.06–7.32 (m, 3H, oxindole-H6, NH2, D2O exchangeable), 8.59, 9.07 (2*s*, 1H, oxindole-H4), 10.15–10.73 (s, 1H, oxindole-NH_,_ D_2_O exchangeable); ^**13**^**C NMR (DMSO-d6)** δ**:** 112.6, 115.7, 118.3, 123.2, 125.1, 138.3, 146.2, 159.8, 170.2; **MS**: (Mwt.: 393.42): *m/z* (% rel. Int.), 240.23 (M^+^, 16.51%), 105.29 (100%); **Anal.** Calcd. for C_9_H_8_N_2_O_4_S: C, 45.00; H, 3.36; N, 11.66; S, 13.35; Found: C, 45.28; H, 3.47; N, 11.92; S, 13.29.

#### General procedure for preparation of target compounds (91a–c, 111a–c, 131a–c and 151a–c)

The appropriate amine **(81a–c, 121a–c, 141a–c** and **161a–c)** (0.39 mmol) was added to a solution of **(2)** (2.65 mmol, 0.5 g) in acetic acid (3 mL) and the reaction mixture was heated under reflux for 4 h., then the acetic acid was evaporated under vacuum, the residue was washed three times 3 × 10 ml with diethyl ether to furnish compounds **(91a–c** and **151a–c)**. compounds (**111a–c**) recrystallized from ethanol/1,4-dioxane. Compounds (**131a–c**) purified by thin layer plate chromatography using (CH_2_Cl_2_: methanol 9:1) as eluent to afford the pure desired compounds.

##### 4-{[(2-Oxoindolin-3-ylidene)methyl]amino}-*N*-(4-sulfamoylphenyl)benzamide (9a)

Yield 89%, mp > 300 °C. IR **(KBr, *ύ* cm**^**−1**^**)**: 3266, 3359 (NH, NH_2_), 3083 (CH aromatic), 1686, (CO), 1591 (NH bending), 1516 (C=C aromatic), 1182, 1396 (SO_2_); ^**1**^**H NMR (DMSO-d6, 400 MHz)** δ ppm: 6.86 (d, 1H, oxindole-H7, *J* = 11.2 Hz*),* 6.95 (t, 1H, oxindole-H5*, J* = 8.4 Hz), 7.04 (t, 1H, oxindole-H6, *J* = 8.4 Hz), 7.28 (s, 2H, SO_2_NH_2,_ D_2_O exchangeable), 7.55 (d, 2H, benzenesulfonamide-H3, H5, *J* = 8.8 Hz), 7.64 (d, 1H, –C=CH–, *J* = 3.6 Hz), 7.8 (d, 2H, benzenesulfonamide-H2, H6, *J* = 8.8 Hz), 7.96 (d, 2H, Ar–H, *J* = 9.6 Hz), 8.03 (d, 2H, Ar–H, *J* = 9.6 Hz), 8.67 (d, 1H, oxindole-H4, *J* = 10.4 Hz), 10.45 (s, 1H, oxindole-NH_,_ D_2_O exchangeable), 10.57 (s, 1H, NH_,_ D_2_O exchangeable), 10.86 (d, 1H, –C=CH–NH_,_ D_2_O exchangeable); ^**13**^**C NMR (DMSO-d6)** δ**:** 101.8, 109.8, 115.6, 120.2, 121.0, 124.3, 125.4, 126.9, 128.5, 130.1, 133.5, 137.3, 137.9, 139.0, 142.7, 143.9, 165.3, 170.3; **Anal.** Calcd. for C_22_H1_8_N_4_O_4_S: C, 60.82; H, 4.18; N, 12.90; S, 7.38; Found: C, 60.71; H, 4.34; N, 13.09; S, 7.45.

##### 4-{[(2-Oxoindolin-3-ylidene)methyl]amino}-N-{4-[N-(pyrimidin-2-yl)sulfamoyl]phenyl}benzamide (9b)

Yield 81%, m.p > 300 °C. **IR (KBr, *ύ* cm**^**−1**^**):** 3263–3382 broad band (4NH), 3081 (CH aromatic), 1704, 1760 (2CO), 1514–1578 (C=C aromatic), 1187, 1379 (SO_2_); ^**1**^**H. NMR (DMSO-d6, 400 MHz)** δ ppm: 6.86 (d, 1H, oxindole-H7, *J* = 8 Hz*),* 6.94 (t, 1H, oxindole-H5*, J* = 8 Hz), 7.04 (m, 2H, oxindole-H6, pyrimidine-H5), 7.54 (d, 2H, benzenesulfonamide-H3,5, *J* = 8 Hz), 7.63 (d, 1H, –C=CH–, *J* = 8 Hz), 7.98–8.01 (m, 4H, Ar–H), 8.02 (d, 2H, benzenesulfonamide-H2,6, *J* = 7.6 Hz), 8.51 (d, 2H, pyrimidine-H4,6, *J* = 4 Hz), 8.64 (d, 1H, oxindole-H4, *J* = 16 Hz), 10.47 (s, 1H, NH_,_ D_2_O exchangeable), 10.57 (s, 1H, oxindole-NH_,_ D_2_O exchangeable), 10.84 (d, 1H, C=CH–NH_,_ D_2_Oexchangeable), 11.8 (s, 1H, SO_2_NH_,_ D_2_O exchangeable); ^**13**^**C NMR (DMSO-d6)** δ**:** 101.9, 109.8, 115.6, 116.2, 118.0, 120.00, 121.0, 124.3, 125.1, 128.4, 129.1, 130.20, 134.7, 137.2, 137.90, 143.6, 157.4, 158.8, 165.6, 170.3, 172.5; **MS**: (Mwt.: 512.54): *m/z* (% rel. Int.), 512.27 (M^+^, 1.85%), 513.39 (M^+^ + 1, 7.12%), 514.30 (M^+^ + 2, 4.93%), 144.30 (100%); **Anal.** Calcd. for C_26_H1_20_N_6_O_4_S: C, 60.93; H, 3.93; N, 16.40; S, 6.26; Found: C, 60.75; H, 4.09; N, 16.67; S, 6.40.

##### 4-{[(2-Oxoindolin-3-ylidene)methyl]amino}-*N*-(thiazol-2-yl)benzamide (9c)

Yield 85%, mp > 300 °C. **IR**: **(KBr, *ύ* cm**^**−1**^**):** 3166–3359 (3NH), 1656 (CO), 1527 (C=C aromatic); ^**1**^**H NMR (DMSO-d6, 400 MHz)** δ ppm: 6.86 (d, 1H, oxindole-H7 of, *J* = 8 Hz), 6.97 (t, 1H, oxindole-H5*, J* = 8 Hz), 7.04 (t, 1H, oxindole-H6, *J* = 8 Hz), 7.26 (d, 1H, thiazole-H5), 7.35 (m, 3H, Ar–H, thiazole-H4), 7.63 (d, 1H, –C=CH–, *J* = 8 Hz), 8.14 (d, 2H, Ar–H, *J* = 8 Hz),), 8.67 (d, 1H, oxindole-H4, *J* = 12 Hz), 10.58 (s, 1H, oxindole-NH_,_ D_2_O exchangeable), 10.84 (d, 1H, –C=CH–NH_,_ D_2_O exchangeable), 12.49 (s, 1H, NH, D_2_O exchangeable); ^**13**^**C NMR (DMSO-d6)** δ**:** 102.1, 107.8, 109.7, 114.1, 115.7, 118.0, 121.0, 124.3, 125.2, 126.2, 130.4, 137.1, 137.9, 144.10, 164.4, 166.4, 170.3; **MS**: (Mwt.: 362.41): *m/z* (% rel. Int.), 362.49 (M^+^, 19.50%), 102.24 (100%); **Anal.** Calcd. for C_19_H1_14_N_4_O_2_S: C, 62.97; H, 3.89; N, 15.46; S, 8.85; Found C, 61.15; H, 4.05; N, 15.70; S, 8.91.

##### 4-{[4-({[2-Oxoindolin-3-ylidene]methyl}amino)benzyl] amino}benzenesulfonamide (11a)

Yield 65%, mp: 195–200 °C. **IR**: **(KBr, *ύ* cm**^**−1**^**):** 3116–3400 broad band (3NH, NH_2_), 3063 (CH aromatic), 1668 (CO), 1593 (NH bending), 1518 (C=C aromatic); ^**1**^**H NMR (DMSO-d6, 400 MHz))** δ ppm: 3.9 (brs, 2H, –CH_2_*)*, 6.86 (d, 1H, oxindole-H7, *J* = 8 Hz*)*, 6.95 (t, 1H oxindole-H5*, J* = 8 Hz), 7.04 (t, 1H, oxindole-H6, *J* = 8 Hz), 7.27–7.35 (m, 6H, Ar–H, SO_2_NH_2,_ D_2_O exchangeable), 7.56 (d, 2H, Ar–H, *J* = 8 Hz), 7.62 (d, 1H, –C=CH–, *J* = 8 Hz), 7.75–7.81 (m, 2H, Ar–H), 8.56 (brs, 1H, oxindole-H4), 10.3 (s, 1H, oxindole-NH_,_ D_2_O exchangeable), 10.51 (s, 1H, NH_,_ D_2_O exchangeable), 10.61 (s, 1H, NH_,_ D_2_O exchangeable)); **MS**: (Mwt.: 420.49): *m/z* (% rel. Int.), 420.14 (M^+^, 16.51%), 261.43 (100%); **Anal.** Calcd. for C_22_H1_20_N_4_O_3_S: C, 62.84; H, 4.79; N, 13.32; S, 7.62; Found C, 63.09; H, 4.86; N, 13.58; S, 7.74.

##### 4-{[4-({[2-Oxoindolin-3-ylidene]methyl}amino)benzyl]amino}-*N*-(pyrimidin-2-yl)benzenesulfonamide (11b)

Yield 60%, mp: 168–171 °C. **IR**: **(KBr, *ύ* cm**^**−1**^**):** 3154–3333 broad band (4NH), 1588 (NH bending), 1667 (CO), 1518 (C=C aromatic), 1186, 1369 (SO_2_); ^**1**^**H NMR (DMSO-d6, 400 MHz))** δ ppm: 3.93 (m, 2H, –CH_2_*)*, 6.86 (d, 1 H, oxindole-H7, *J* = 8 Hz), 6.9–7.03 (m, 3H, oxindole-H5,H6*,* pyrimidine-H5), 7.23–7.4 (m, 4H, Ar–H), 7.53–7.64 (m, 3H, Ar–H, –C=CH–), 7.9 (d, 2H, Ar–H, *J* = 10 Hz), 8.53–8.69 (m, 3H, oxindole-H4, pyrimidine-H3,H5), 10.14 (s, 1H, NH_,_ D_2_O exchangeable), 10.46 (s, 1H, oxindole-NH_,_ D_2_O exchangeable), 10.58 (d, 1H, –C=CH–NH_,_ D_2_O exchangeable). 10.87 (s, 1H, SO_2_NH_,_ D_2_O exchangeable); %); **Anal.** Calcd. for C_26_H1_22_N_6_O_3_S: C, 62.64; H, 4.45; N, 16.86; S, 6.43; Found: C, 62.85; H, 4.61; N, 17.09; S, 6.03.

##### 3-{[(4-((Thiazol-2-ylamino)methyl)phenyl)amino]methylene}indolin-2-one (11c)

Yield 77%, mp: 150–153 °C. **IR**: **(KBr, *ύ* cm**^**−1**^**):** 3119–3232 broad band (3NH), 3082 (CH aromatic), 1671 (CO), 1592 (NH bending), 1521 (C=C aromatic); ^**1**^**H NMR (DMSO-d6, 400 MHz))** δ ppm: 4.1 (s, 2H, –CH_2_), 6.86–7.09 (m, 3H, oxindole-H5, H6, H7*)*, 7.35–7.39 (m, 2H, thiazole-H5, H4), 7.57 (d, 2H, Ar–H, *J* = 8 Hz), 7.63 (d, 1H, –C=CH–, *J* = *6.*8 Hz), 7.89 (d, 2H, Ar–H, *J* = 10.8 Hz), 8.66 (d, 1H, oxindole-H4, *J* = 12 Hz), 10.4 ((s, 1H, oxindole-NH_,_ D_2_O exchangeable), 10.58 (s, 1H, NH_,_ D_2_O exchangeable), 10.9 (d, 1H, –C=CH–NH_,_ D_2_O exchangeable, *J* = 10.8 Hz), **MS**: (Mwt.: 348.42): *m/z* (% rel. Int.), 348.34 (M^+^, 9.91%), 64.37 (100%); **Anal.** Calcd. for C_19_H1_16_N_4_OS: C, 65.50; H, 4.63; N, 16.08; S, 9.20; Found: C, 65.28; H, 4.74; N, 18.31; S, 9.03.

##### 4-{[-3-Oxo-3-(4-{[(-2-oxoindolin-3-ylidene)methyl]amino}phenyl)prop-1-en-1-yl]amino}benzenesulfonamide (13a)

Yield 73%, mp: 275–278 °C, **Z/E mixture 1:1**. **IR (KBr, *ύ* cm**^**−1**^**):** 3216–3363 broad band (3NH, NH_2_), 3083 (CH aromatic), 1643, 1677 (2CO), 1589 (NH bending), 1183, 1370 (SO_2_)**; **^**1**^**H NMR (DMSO-d6, 400 MHz)** δ ppm: 6.27, 6.58 (2d, 1 H, –CH=CH–NH, *J* = 8, 12 Hz), 6.87 (d, 1H, oxindole-H7, *J* = 8 Hz*),* 6.95 (t, 1H, oxindole-H5*, J* = 8 Hz), 7.05 (t, 1H, oxindole-H6_,_ *J* = 8 Hz), 7.27–7.33 (m, 3H, Ar–H, SO_2_NH_2,_ D_2_O exchangeable), 7.51 (d, 2H, Ar–H, *J* = 8 Hz), 7.64 (d, 1H, –C=CH–, *J* = 8 Hz), 7.76–7.78, 8.16 (m, 3 H, Ar–H, –CH=CH–NH), 7.92–7.99 (m, 2H, Ar–H), 8.03 (d, 1H, Ar–H, *J* = 8 Hz), 8.69 (brs, 1H, oxindole-H4), 10.36, 12.07 (2d, 1 H, –CO–CH=CH–NH, D_2_O exchangeable, J = 12 Hz), 10.61 (s, 1H, NH, D_2_O exchangeable), 10.89 (s, 1H, –C=CH–NH_,_ D_2_O exchangeable); ^**13**^**C NMR (DMSO-d6)** δ**:** 95.2, 99.8 (–CO–CH=CH), 102.0, 109.8, 115.3, 116.2, 118.0, 121.0, 124.3, 127.1, 127.9, 129.8, 137.1, 137.8, 138.4, 143.20, 143.4, 143.7, 144.4, 170.3 (CO of oxindole), 186.5, 189.0 (–CO–CH=CH); **MS**: (Mwt.: 460.51): *m/z* (% rel. Int.), 460.86 (M^+^, 10.18%), 235.27 (100%); **Anal.** Calcd. for C_24_H1_20_N_4_O_4_S: C, 62.60; H, 4.38; N, 12.17; S, 6.96; Found: C, 62.47; H, 4.54; N, 12.49; S, 6.85.

##### 4-{[3-Oxo-3-(4-{[(2-oxoindolin-3-ylidene)methyl]amino}phenyl)prop-1-en-1-yl]amino}-N-(pyrimidin-2-yl)benzenesulfonamide (13b)

Yield 70%, mp: 290–294 °C, **Z/E mixture 1:1**. **IR (KBr, *ύ* cm**^**−1**^**):** 3000–3375 broad band (4NH), 2313 (N–CH), 1600, 1668 (2CO), 1573 (NH bending), 1157, 1340 (SO_2_)**; **^**1**^**H NMR (DMSO-d6, 400 MHz)** δ ppm: 6.27, 6.58 (2d, 1 H, –CH=CH–NH, *J* = 8, 12 Hz), 6.87 (d, 1H, oxindole-H7, *J* = 8 Hz*),* 6.95 (t, 1H, oxindole-H5*, J* = 8 Hz), 7.04 (t, 2H, oxindole-H6_,_ pyrimidin-H5), 7.29 (d, 1H, Ar–H, *J* = 8 Hz), 7.51 (d, 2H, Ar–H, *J* = 8 Hz), 7.64 (d, 1H, –C=CH–, *J* = 8 Hz), 7.78–7.95, 8.12 (m, t, 5 H, Ar–H, –CH=CH–NH, *J* = 12 Hz), 8.03 (d, 1H, Ar–H, *J* = 8 Hz), 8.52 (d, 2H, pyrimidine-H2,4, *J* = 8 Hz), 8.68 (d, 1H, oxindole-H4, *J* = 12 Hz), 10.39, 12.05 (2d, 1 H, –CO–CH=CH–NH, D_2_O exchangeable,* J* = 12 Hz), 10.61 (s, 1H, oxindole-NH_,_ D_2_O exchangeable), 10.87–10.91 (m, 1H, –C=CH–NH_,_ D_2_O exchangeable), 11.75 (s, 1H, SO_2_NH_,_ D_2_O exchangeable; ^**13**^**C NMR (DMSO-d6)** δ**:** 95.65, 100.42 (–CO–CH=CH, cis and trans conformers), 101.9, 109.80, 115.1, 115.8, 118.0, 121.0, 124.3, 129.8, 130.0, 130.2, 133.7, 137.2, 137.9, 142.8, 143.80, 144.3, 145.5, 148.4, 157.4, 158.8, 170.3, (CO of oxindole), 186.5, 189.2 (–CO–CH=CH, cis and trans conformers); **MS**: (Mwt.: 538.58): *m/z* (% rel. Int.), 538.53 (M^+^, 6.47%), 185.30 (100%); **Anal.** Calcd. for C_28_H1_22_N_6_O_4_S: C, 62.44; H, 4.12; N, 15.60; S, 5.95; Found: C, 62.31; H, 4.29; N, 15.87; S, 6.03.

##### 3-{[(4-{3-[Thiazol-2-ylamino]acryloyl}phenyl)amino]methylene}indolin-2-one (13c)

Yield 73%, mp: 260–262 °C, **Z/E mixture 1:1** ^**1**^**H NMR (DMSO-d6, 400 MHz)** δ ppm: 5.68, 6.3, 6.55, 6.69 (4d, 1 H, –CO–CH=CH–NH, *J* = 4 Hz), 6.88 (d, 1H, oxindole-H7, *J* = 8 Hz*),* 6.94–7.01 (m, 1H, oxindole-H5), 7.03–7.07 (m, 1H, oxindole-H6), 7.4 (brs, 1H, thiazole-H5), 7.51 (d, 1H, thiazole-H4, *J* = 8 Hz), 7.61–7.66 (m, 2H, Ar–H), 7.69 (d, 1H, –C=CH–, *J* = 8 Hz), 7.79–7.88, 8.38 (m, brs, 2H, –CO–CH=CH–NH, Ar–H), 7.97 (d, 1H, Ar–H, *J* = 8 Hz), 8.64 (d, 1H, oxindole-H4, *J* = 12 Hz), 10.55, 10.69 (2*s*, 1H, oxindole-NH_,_ D_2_O exchangeable), 10.63, 11.00 (s, d, 1H, –CO–CH=CH–NH_,_ D_2_O exchangeable, *J* = 12 Hz), 10.81–10.9 (m, 1H, –C=CH–NH_,_ D_2_O exchangeable); **MS**: (Mwt.: 388.45): *m/z* (% rel. Int.), 388.31 (M^+^, 12.46%), 317.94 (100%); **Anal.** Calcd. for C_21_H_16_N_4_O_2_S: C, 64.93; H, 4.15; N, 14.42; S, 8.25; Found: C, 65.19; H, 4.28; N, 14.7; S, 8.33.

##### 4-{3-[4-({[2-Oxoindolin-3-ylidene]methyl}amino)phenyl]ureido}benzenesulfonamide (15a)

Yield 73%, m.p: 270–275 °C, **IR (KBr, *ύ* cm**^**−1**^**):** 1162, 1369 (SO_2_), 1604, 1653 (2CO), 3249–3373 broad band (5NH)**; **^**1**^**H NMR (DMSO-d6, 400 MHz)** δ ppm: 6.84 (d, 1H, oxindole-H7*)*, 6.9–7.0 (m, 2H, oxindole-H5, H6), 7.21 (s, 2H, SO_2_NH_2,_ D_2_O exchangeable), 7.34 (d, 2H, Ar–H), 7.48 (d, 2H, Ar–H), 7.57 (d, 1H, –C=CH–), 7.61 (d, 2H, Ar–H), 7.72 (d, 2H, Ar–H,), 8.52 (m, 1H, oxindole-H4), 8.78 (s, 1H, NHCONH, D_2_O exchangeable), 9.06 (s, 1H, NHCONH_,_ D_2_O exchangeable), 10.46 (s, 1H, oxindole-NH_,_ D_2_O exchangeable), 10.71 (d, 1H, –C=CH–NH_,_ D_2_O exchangeable, *J* = 11.2 Hz); ^**13**^**C NMR (DMSO-d6)** δ**:** 99.38, 109.57, 116.98, 117.27, 117.92, 120.34, 120.76, 124.09, 124.80, 124.84, 127.30, 135.40, 137.22, 137.47, 138.69, 143.37, 152.77, 170.34; **MS**: (Mwt.: 527.56): *m/z* (% rel. Int.), 527.40 (M^+^, 25.17%), 144.23 (100%); **Anal.** Calcd. for C_22_H1_19_N_5_O_4_S: C, 58.79; H, 4.26; N, 15.58; S, 7.13; Found: C, 59.06; H, 4.38; N, 15.84; S, 7.21.

##### 4-{3-[4-({[2-Oxoindolin-3-ylidene]methyl}amino)phenyl]ureido}-N-(pyrimidin-2-yl)benzenesulfonamide (15b)

Yield 68%, mp > 300 °C; **IR (KBr, *ύ* cm**^**−1**^**):** 1182, 1331 (SO_2_), 1671, 1708 (2CO), 3074 (CH aromatic), 3192, 3251, 3294, 3378, 3404 (5NH)**; **^**1**^**H NMR (DMSO-d6, 400 MHz)** δ ppm: 6.84 (d, 1H, oxindole-H7, *J* = 8 Hz), 6.90 (t, 1H, oxindole H-5), 6.98 (t, 1H, oxindole-H6), 7.03 (t, 1H, pyrimidin-H5), 7.34 (d, 2H, Ar–H), 7.45 (d, 2H, Ar–H), 7.55 (d, 1H, –C=CH–), 7.62 (d, 2H, Ar–H), 7.9 (d, 2H, Ar–H), 8.5–8.55 (m, 3H, oxindole-H4, pyrimidin-H2,H4), 8.8 (s, 1H, NHCONH,_,_ D_2_O exchangeable), 9.12 (s, 1H, NHCONH_,_ D_2_O exchangeable), 10.46 (s, 1H, oxindole-NH_,_ D_2_O exchangeable), 10.71 (d, 1H, –C=CH–NH_,_ D_2_O exchangeable, *J* = *9.6* Hz), 11.68 (s, 1H, SO_2_NH_,_ D_2_O exchangeable); ^**13**^**C NMR (DMSO-d6)** δ**:** 99.3, 109.5, 116.2, 116.9, 117.2, 117.6, 120.3, 120.7, 124.2, 124.8, 129.4, 132.8, 135.3, 137.2, 138.8, 144.4, 152.6, 157.50, 158.8, 170.3, 172.5; **MS**: (Mwt.: 527.40): *m/z* (% rel. Int.), 527.43 (M^+^, 25.17%), 108.34 (100%); **Anal.** Calcd. for C_26_H_21_N_7_O_4_S; **Anal.** Calcd. for C_26_H1_21_N_7_O_4_S: C, 59.19; H, 4.01; N, 18.59; S, 6.08; Found: C, 59.45; H, 4.18; N, 18.73; S, 5.97.

##### 1-{4-[((2-Oxoindolin-3-ylidene)methyl)amino]phenyl}-3-(thiazol-2-yl)urea (15c)

Yield 84%, m.p > 300. **IR**: **(KBr, *ύ* cm**^**−1**^**):** 1604, 1667 (2CO), 3166–3375 (4NH), 3083 (CH aromatic); ^**1**^**H NMR (DMSO-d6, 400 MHz)** δ ppm: 6.87–6.94 (m, 2H, oxindole-H7, H5), 6.98–7.02 (m, 1H, oxindole-H6), 7.31 (d, 1H, thiazole-H5), 7.44 (d, 1H, thiazole-H4), 7.53 (d, 1H, –C=CH–, *J* = 8 Hz), 7.58 (d, 2H, Ar–H, *J* = 12 Hz), 7.72 (d, 2H, Ar–H, *J* = 12 Hz),), 8.48 (d, 1H, oxindole-H4), 8.95 (s, 1H, NHCONH,_,_ D_2_O exchangeable), 9.33 (s, 1H, NHCONH_,_ D_2_O exchangeable), 10.47 (s, 1H, oxindole-NH_,_ D_2_O exchangeable), 10.75 (brs, 1H, –C=CH–NH_,_ D_2_O exchangeable); ^**13**^**C NMR (DMSO-d6)** δ**:** 99.80, 109.5, 116.90, 117.6, 120.4, 120.7, 121.5, 124.27, 124.8, 132.2, 136.2, 137.3, 138.4, 138.6, 163.8, 164.9, 170.3; **MS**: (Mwt.: 377.42): *m/z* (% rel. Int.), 377.74 (M^+^, 26.37%), 372.47 (100%); **Anal.** Calcd. for C_19_H1_15_N_5_O_2_S: C, 60.47; H, 4.01; N, 18.56; S, 8.49; Found C, 60.59; H, 4.20; N, 18.82; S, 8.70.

### In vitro biological evaluation

#### In-vitro anti-proliferative activity assay against NCI 60-cell line panel

From the newly synthesized compounds, eleven compounds were selected by the National Cancer Institute (NCI) Developmental Therapeutics Program (DTP), Bethesda, Maryland, USA for the in vitro anti-proliferative activity evaluation (For further details see supplementary materials).

#### In vitro five-dose assay on selected cell lines (MCT-7, HCT-116, and DU 145)

Five dose assay was performed using cancer cell cultures obtained from Nawah Scientific Inc., (Mokatam, Cairo, Egypt), Cell viability was assessed by SRB assay. The output from the dose–response is reported as a mean graph (Additional file 1; S3: In vitro biological activity).

#### Carbonic anhydrase inhibition

An Applied Photophysics stopped-flow instrument has been used for assaying the CA catalyzed CO_2_ hydration activity [82]. The inhibition constants were obtained by non-linear least-squares methods using PRISM 3 and the Cheng-Prusoff equation [83] to represent the mean from at least three different determinations. The four tested CA isoforms were recombinant ones obtained in-house as reported earlier [84].

#### Enzyme inhibitory assay

The FGFR, VEGFR-2 and RET tyrosine kinase assays were carried out at Thermo Fischer Scientific, USA (www.thermofischer.com/selectscreen). (For further details see Additional file 1; S3: In vitro biological activity).

### Molecular docking

Molecular docking was carried out using Molecular Operating Environment software (MOE, 2020.0901). The detailed molecular docking setup used as well as its validation are provided in the Additional file 1; S2: Molecular docking study.

## Supplementary Information

**Additional file 1.** S1. Spectral data. S2. Molecular docking study. S3. In Vitro biological activity.

## Data Availability

Spectral data, molecular modeling and biological evaluation data generated or analysed during this study are included in this published article and its Additional file 1.
