# Supplementary material for: Discovery of a new potent oxindole multi-kinase inhibitor among a series of designed 3-alkenyl-oxindoles with ancillary carbonic anhydrase inhibitory activity as antiproliferative agents
Source: BMC Chem. 2023 Jul 18;17(1):81. doi: 10.1186/s13065-023-00994-3 (PMC10353187; doi:10.1186/s13065-023-00994-3)
Supplement: Supplementary file 1 — Additional file 1. S1. Spectral data. S2. Molecular docking study. S3. In Vitro biological activity. [file 13065_2023_994_MOESM1_ESM.docx]

**Discovery of a New Potent Oxindole Multi-kinase Inhibitor Among a Series of Designed 3-Alkenyl-oxindoles with ancillary Carbonic Anhydrase Inhibitory activity as antiproliferative agents**

Rania S. M. Ismail^1,^*, Ahmed M. El Kerdawy^2,3,^, Dalia H. Soliman^1,4,^, Hanan H. Georgey^2,5^, Nagwa M. Abdel Gawad^2,^*,Andrea Angeli^6^, Claudiu T. Supuran^6^

*^1^ Department of Pharmaceutical Chemistry, Faculty of Pharmacy, Egyptian Russian University, Badr City, Cairo, P.O. Box 11829, Egypt.*

*^2^ Department of Pharmaceutical Chemistry, Faculty of Pharmacy, Cairo University, Kasr El-Aini Street, Cairo, P.O. Box 11562, Egypt.*

*****^3^ Department of Pharmaceutical Chemistry, School of Pharmacy, Newgiza University (NGU), Newgiza, km 22 Cairo–Alexandria Desert Road, Cairo, Egypt.*

*^4^ Department of Pharmaceutical Chemistry, Faculty of Pharmacy, Al-Azhar University, Cairo, P.O. Box 11471, Egypt*

*^5^ Department of pharmaceutical chemistry, Faculty of Pharmacy and Drug Technology, Egyptian Chinese University, 11786, Cairo, Egypt*

*^6^Department of NEUROFARBA, Section of Pharmaceutical and Nutraceutical Sciences, University of Florence, Florence, Italy,*

**Corresponding author: Rania S. M. Ismail, e. mail: rania-saied@eru.edu.eg*

*Nagwa M. Abdel Gawad, e. mail:* [*nagwa.abdelgawad@pharma*](mailto:nagwa.abdelgawad@pharma)*.cu.edu.eg*

*Claudiu T. Supuran, e. mail:* [*claudiu.supuran@unifi.it*](mailto:claudiu.supuran@unifi.it)

**Table of Contents**

[**S1. Spectral data** 2](#_Toc128048588)

[**S2. Molecular docking study** 22](#_Toc128048589)

[**S3. In Vitro biological activity** 31](#_Toc128048590)

**Supporting Materials**

**General remarks**

Melting points were carried out by the open capillary tube method using a Stuart (Stone Staffordshire ST/50SA UK) apparatus and they were uncorrected. Infrared Spectra were performed on Schimadzu FT-IR 8400 S spectrometer Affinity A1 using potassium bromide discs, and expressed in wave number (cm^− 1^). NMR spectra were recorded on a Bruker Ascend 400/R (^1^H: 400 MHz and ^13^C: 100 MHz) spectrophotometer. Chemical shift values (δ) were given in parts per million (ppm) downfield from tetramethylsilane (TMS) as an internal reference. Elemental analyses were carried out using FLASH 2000 CHNS/O analyzer, Thermo Scientific at the Regional Centre for Mycology and Biotechnology (RCMB), Al-Azhar University, Nasr City, Cairo. Mass spectra were carried out on Direct Inlet part to mass analyzer in Thermo Scientific GCMS model ISQ. All the reactions were monitored by thin layer chromatography silica gel F 254, Aluminum sheets 20 × 20 cm (Sigma-Aldrich) were used. Dichloromethane: methanol (1: 0.1) was the adopted elution system.

# **S1. Spectral data**

**
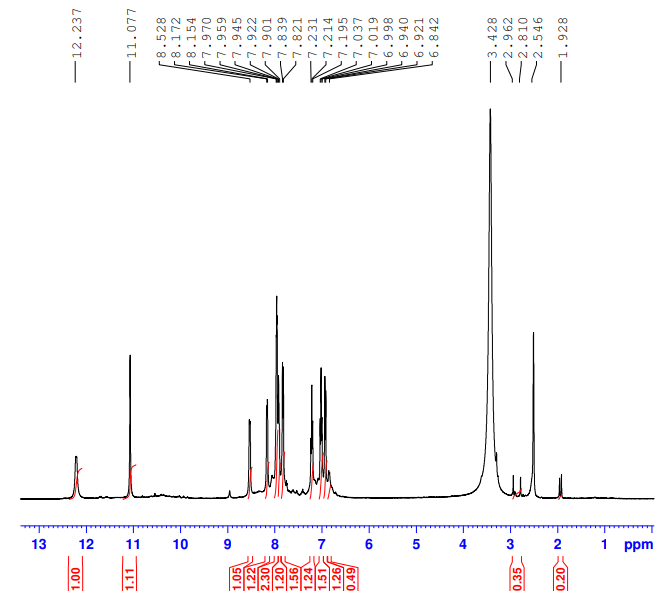
**

**
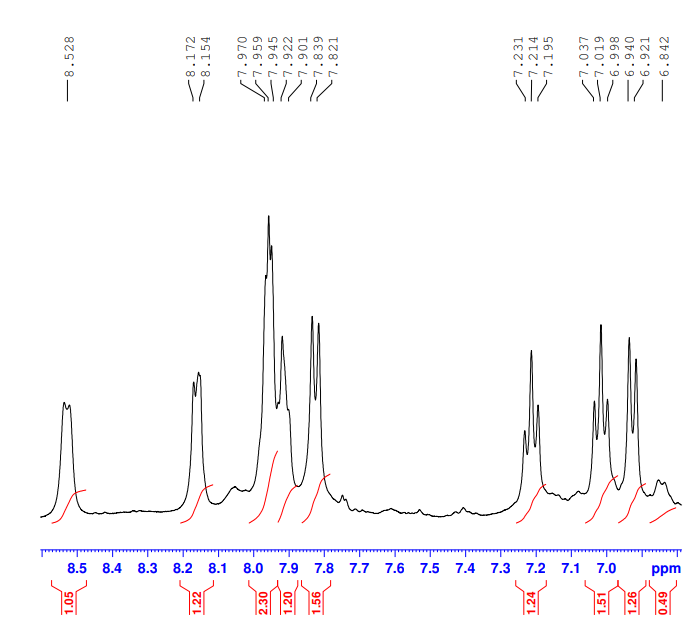
**

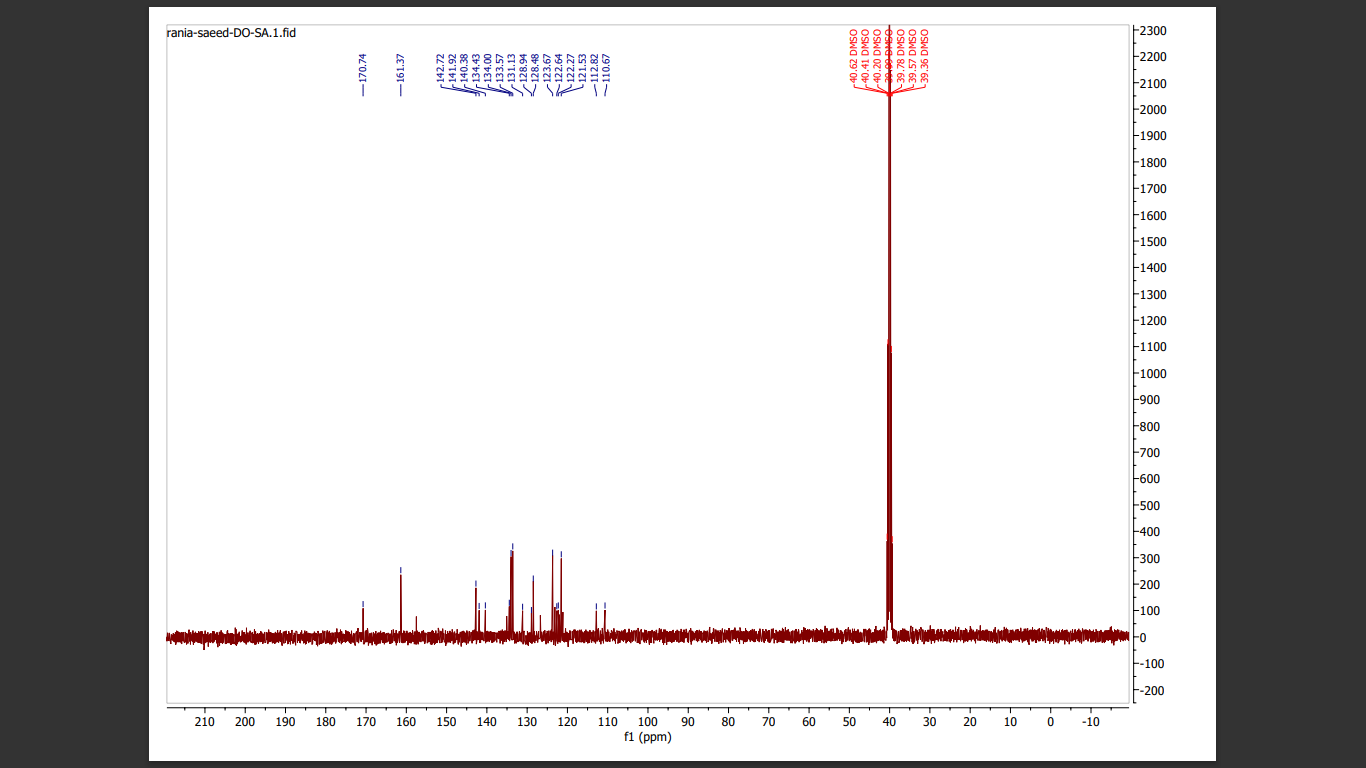

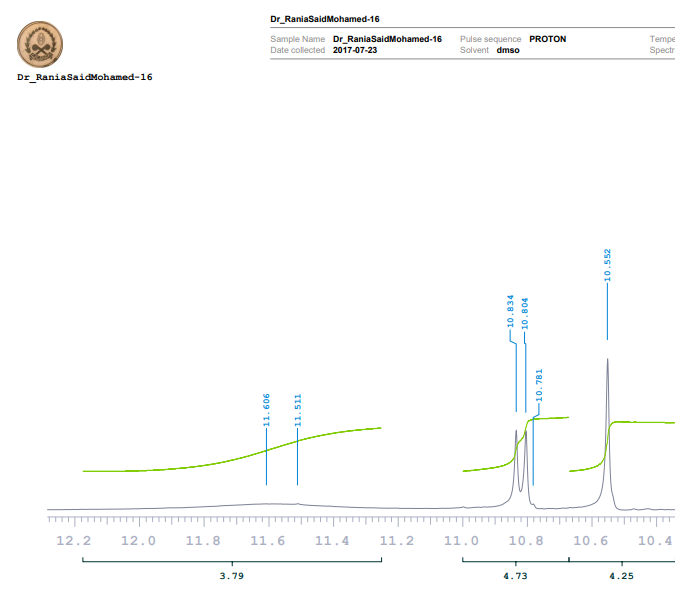


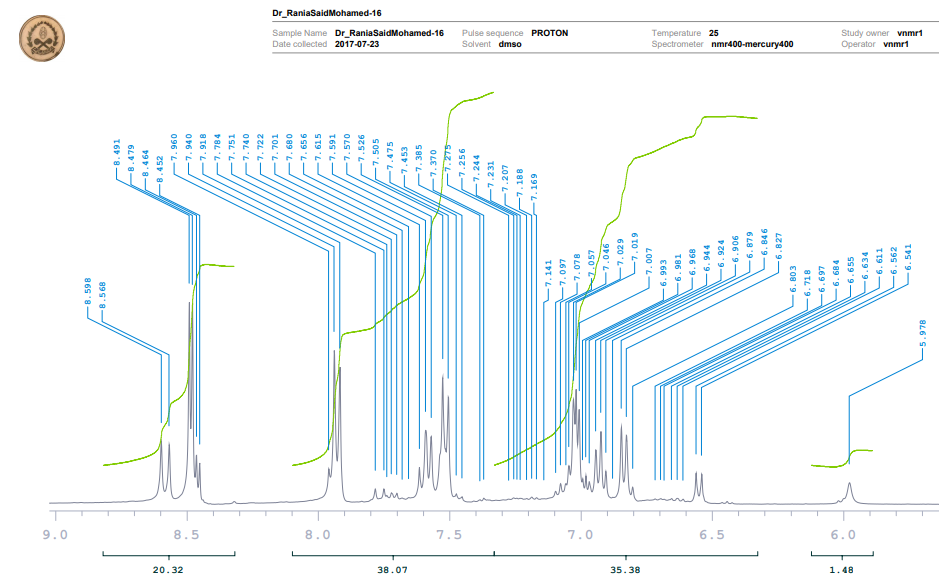

**^
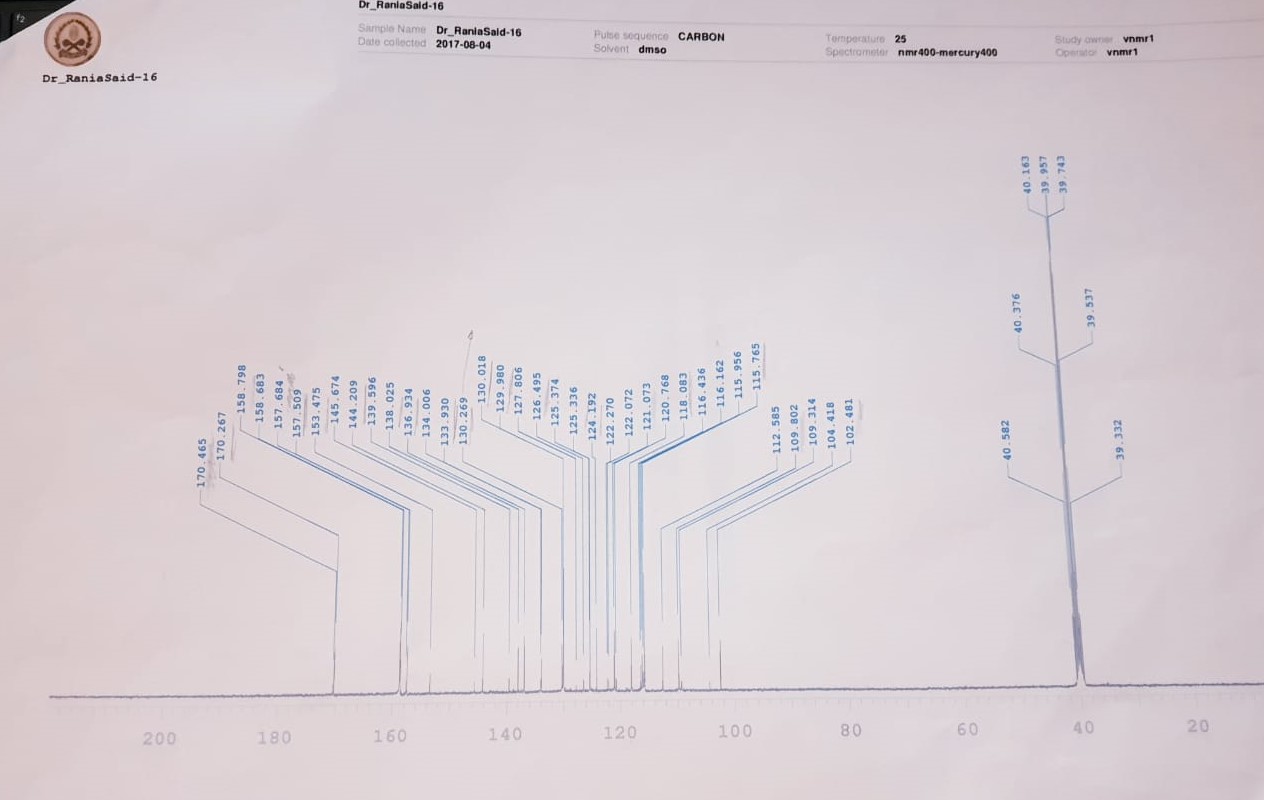
^**


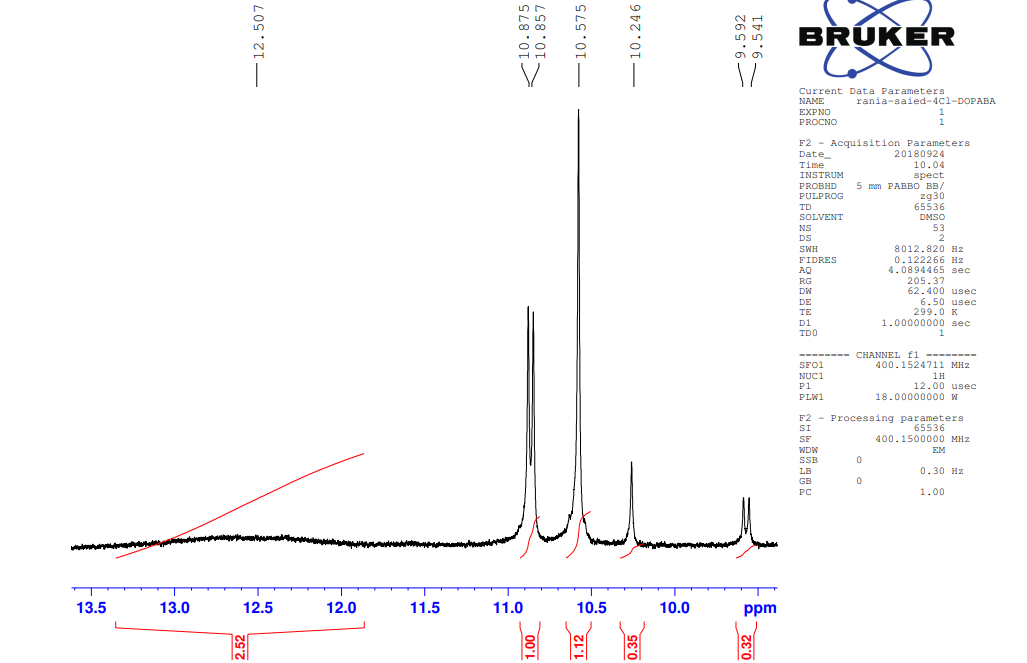

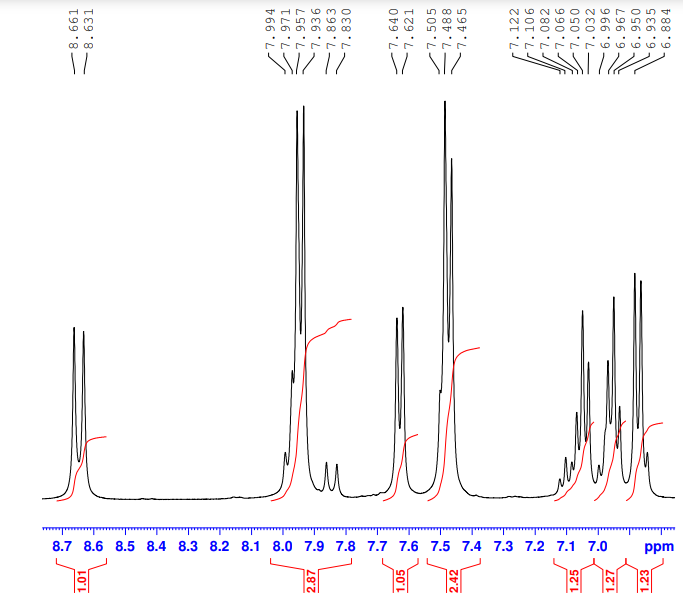


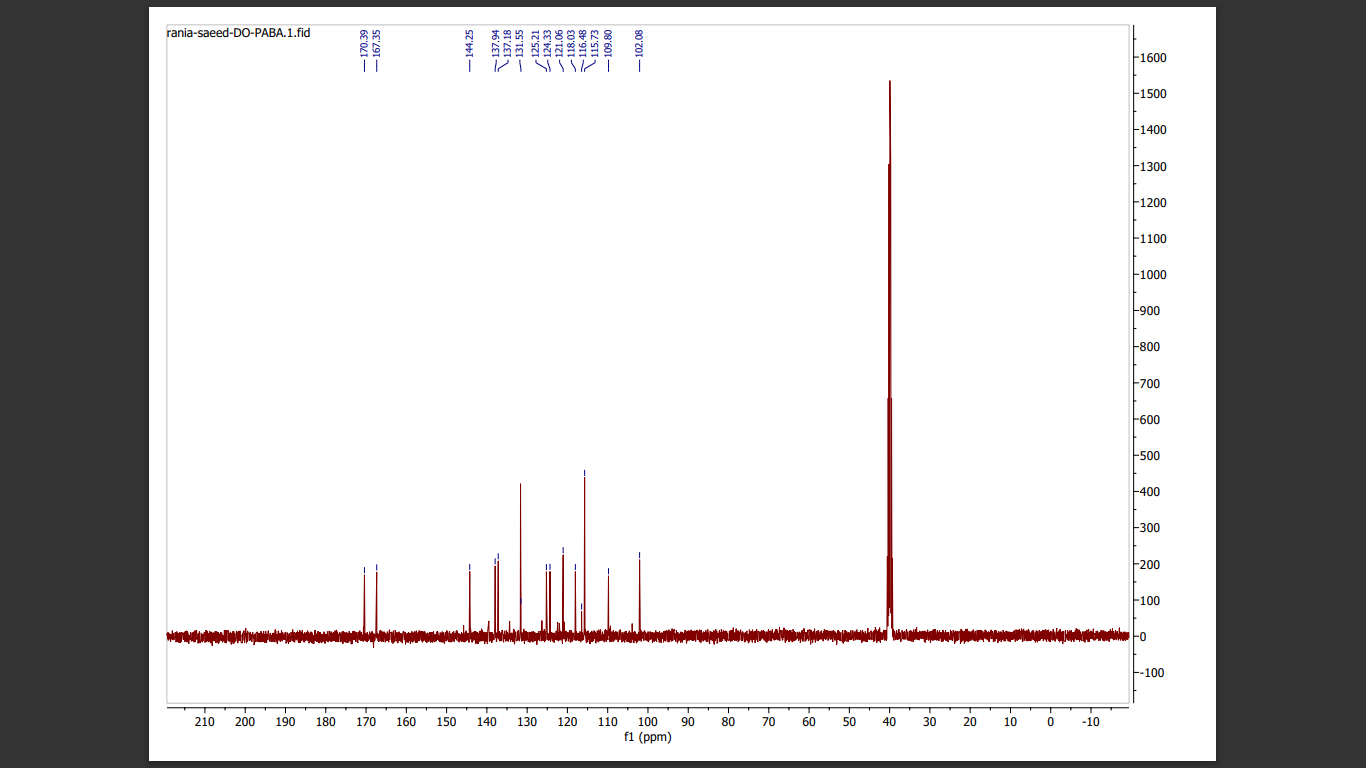

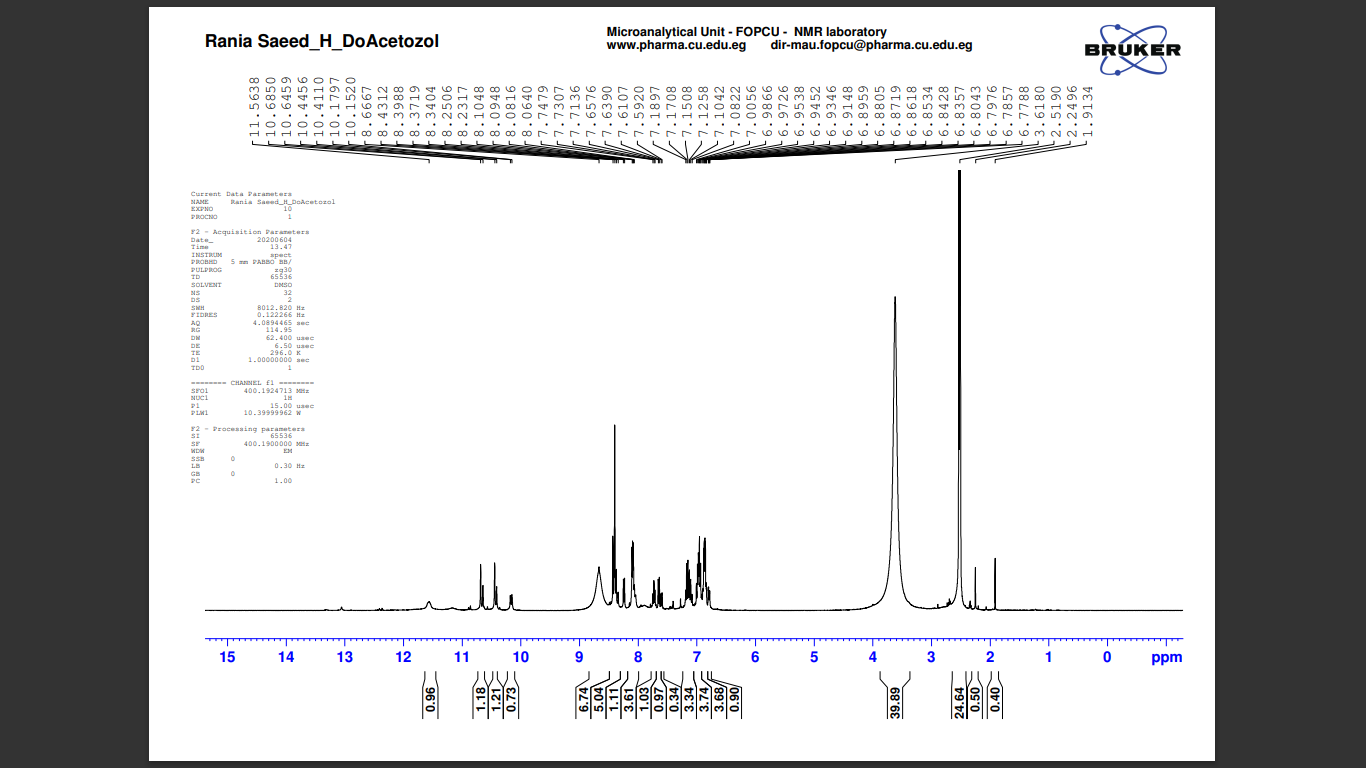

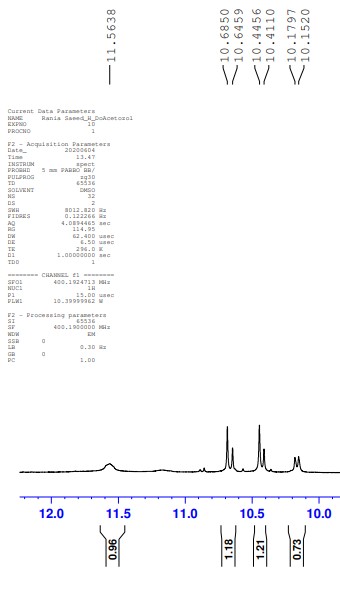

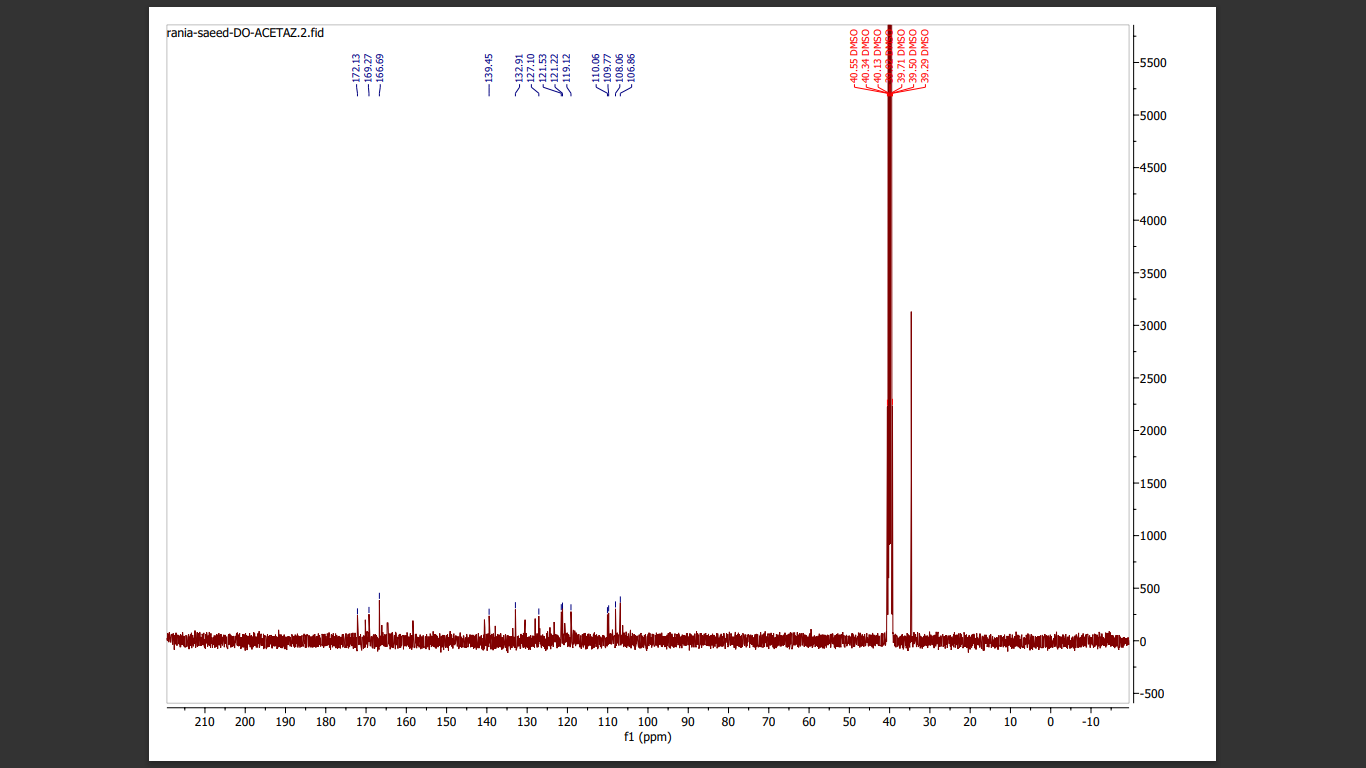


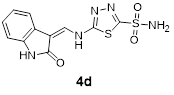


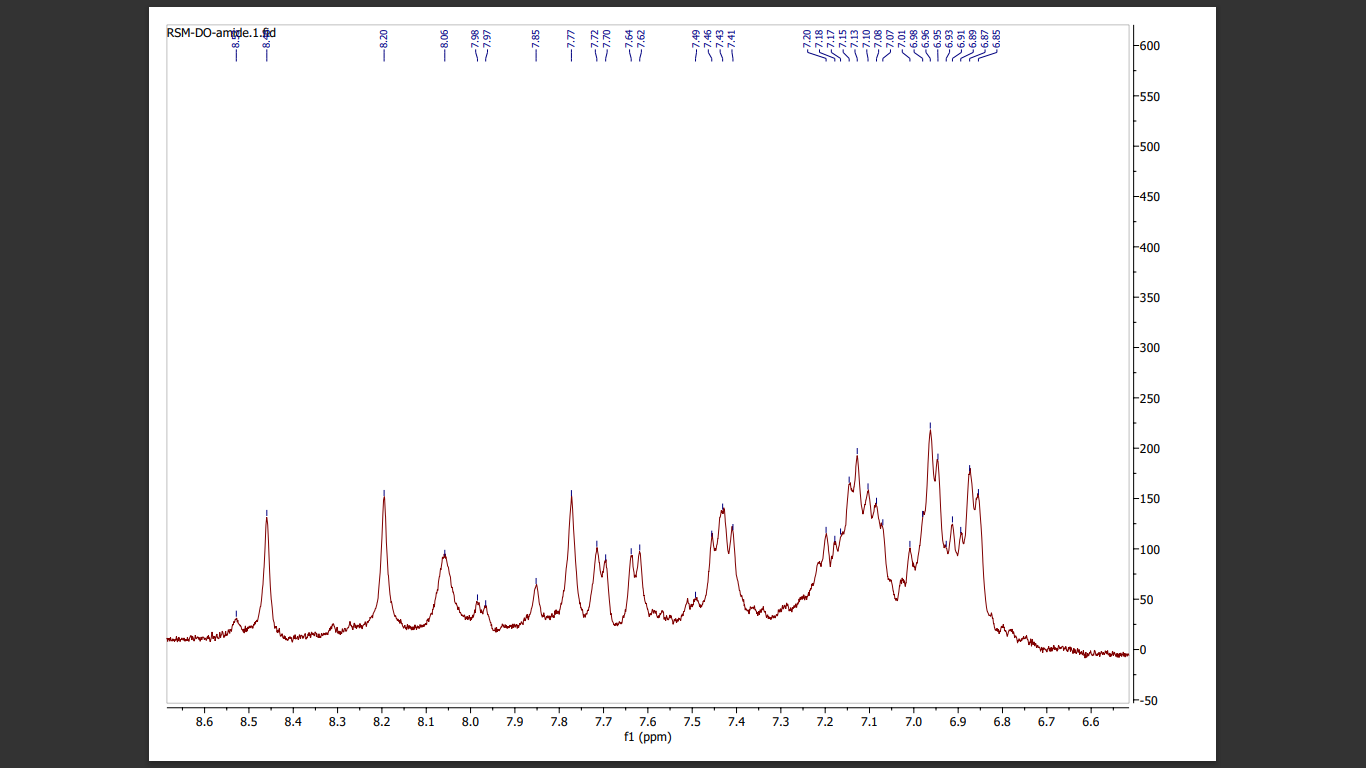

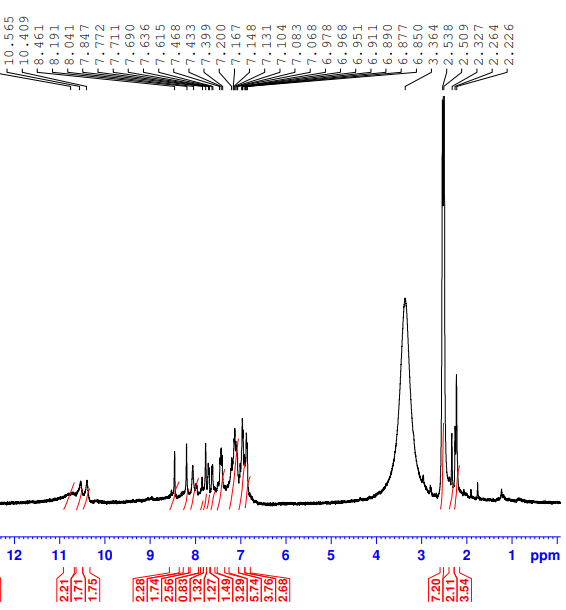


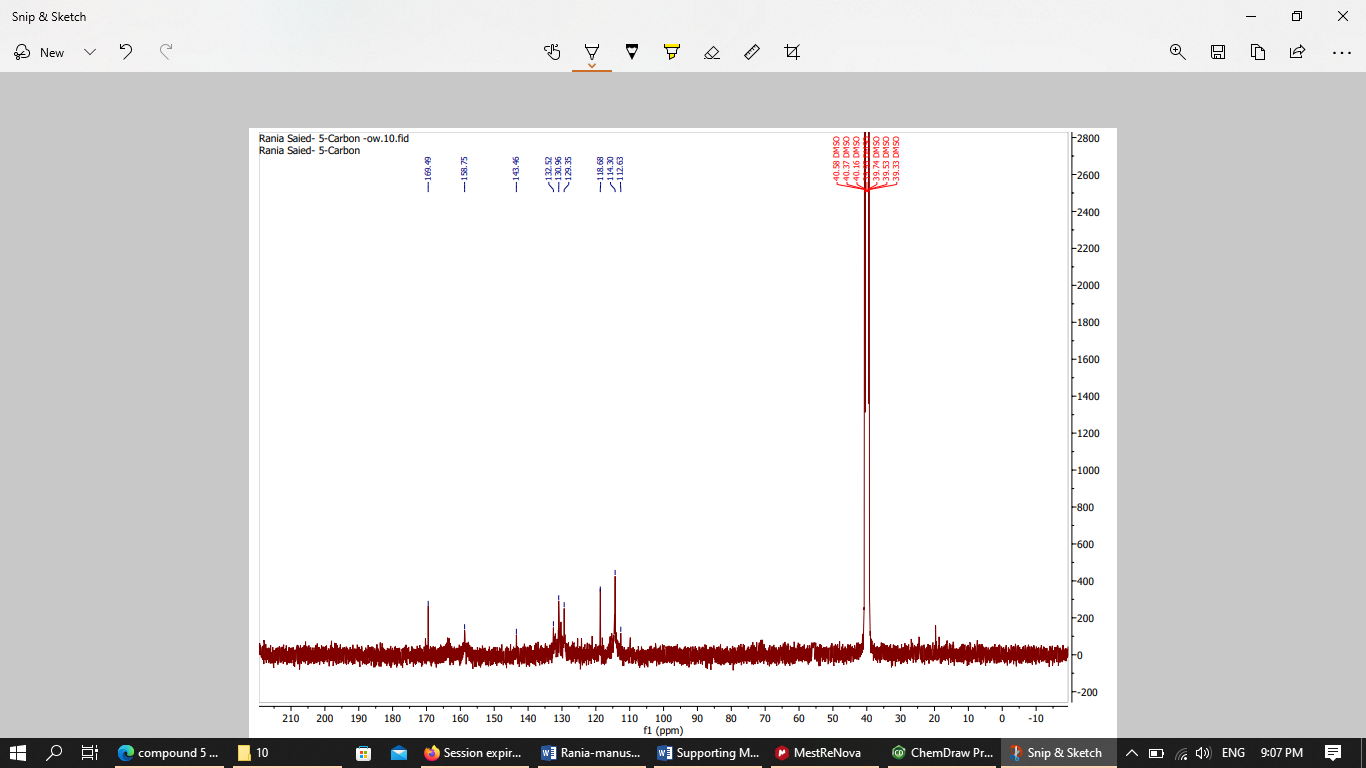

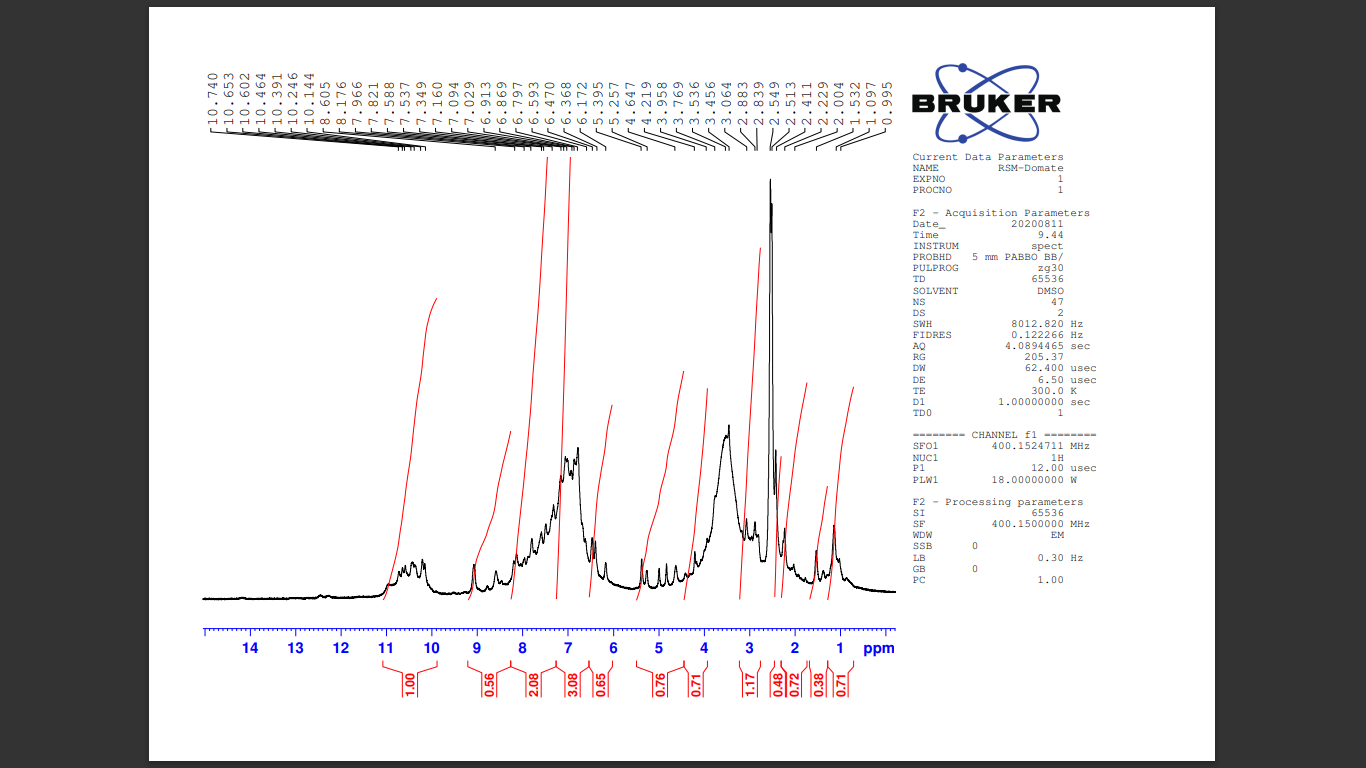

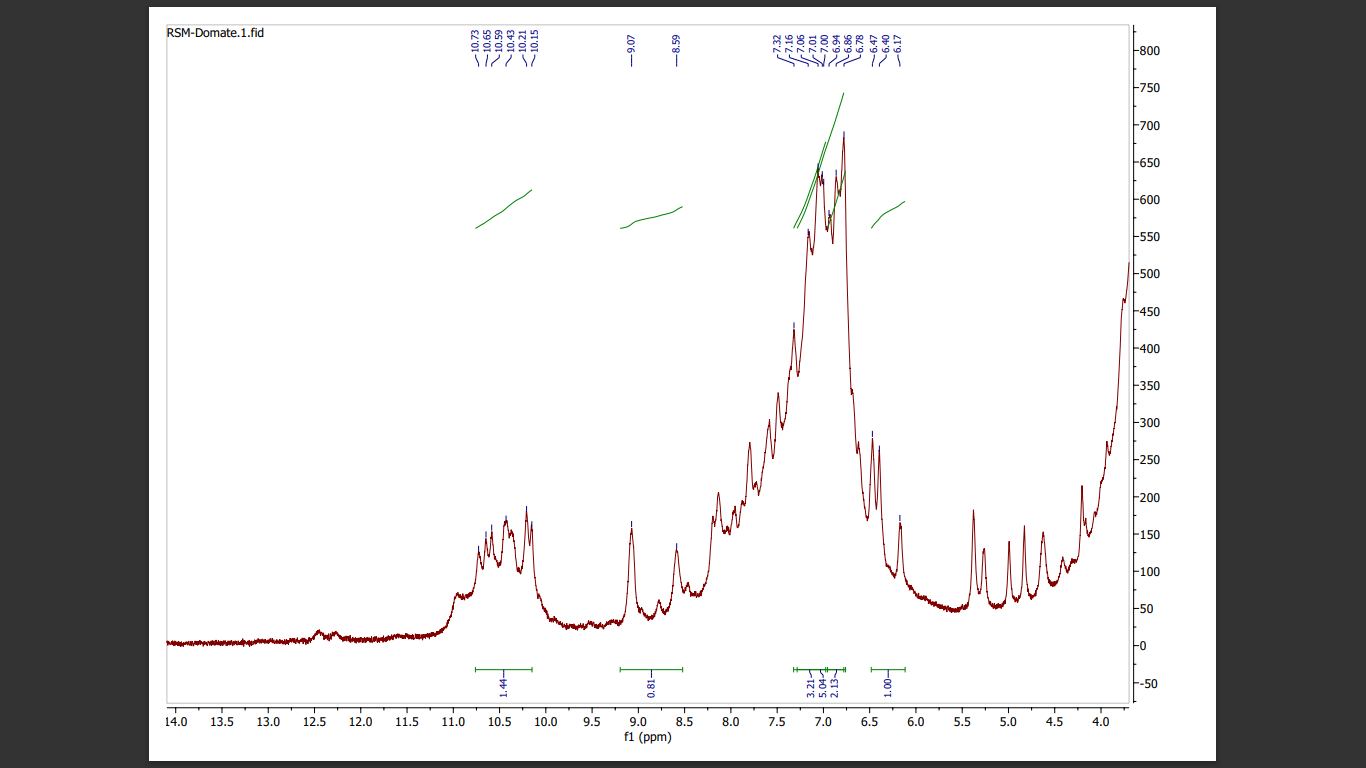

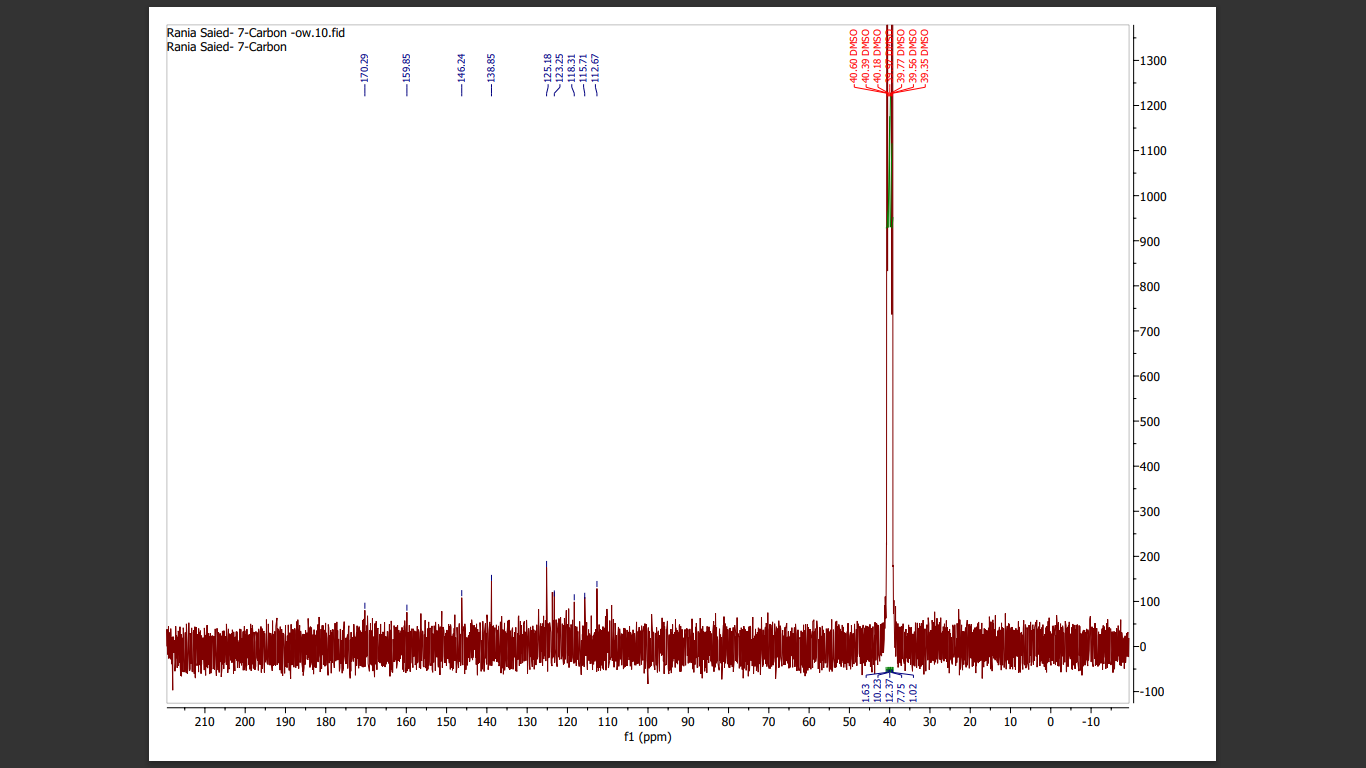

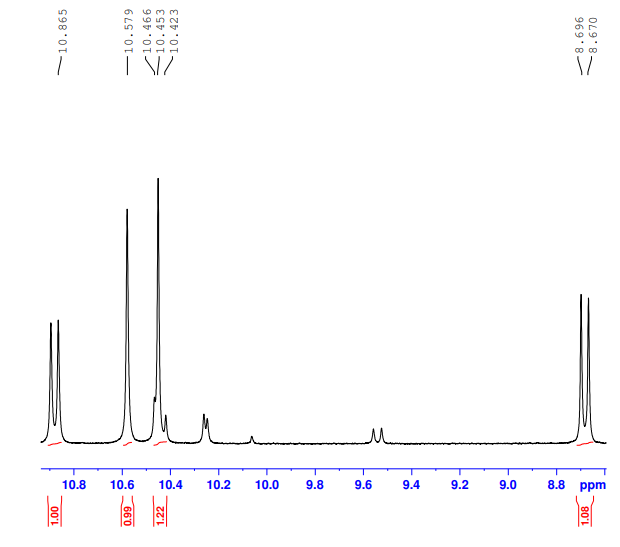

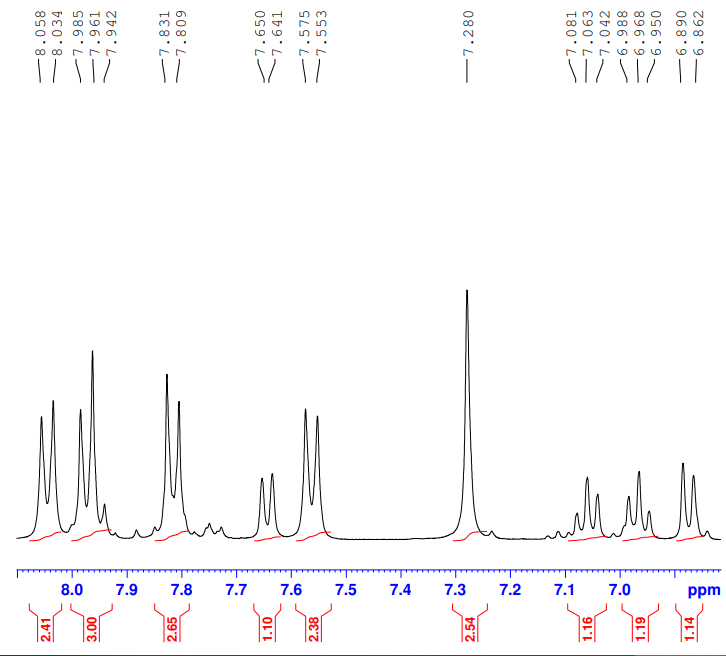

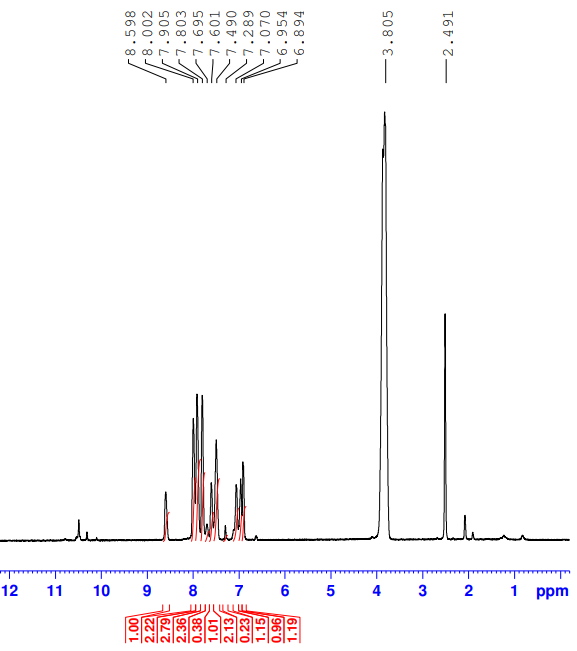


9a-d_2_O

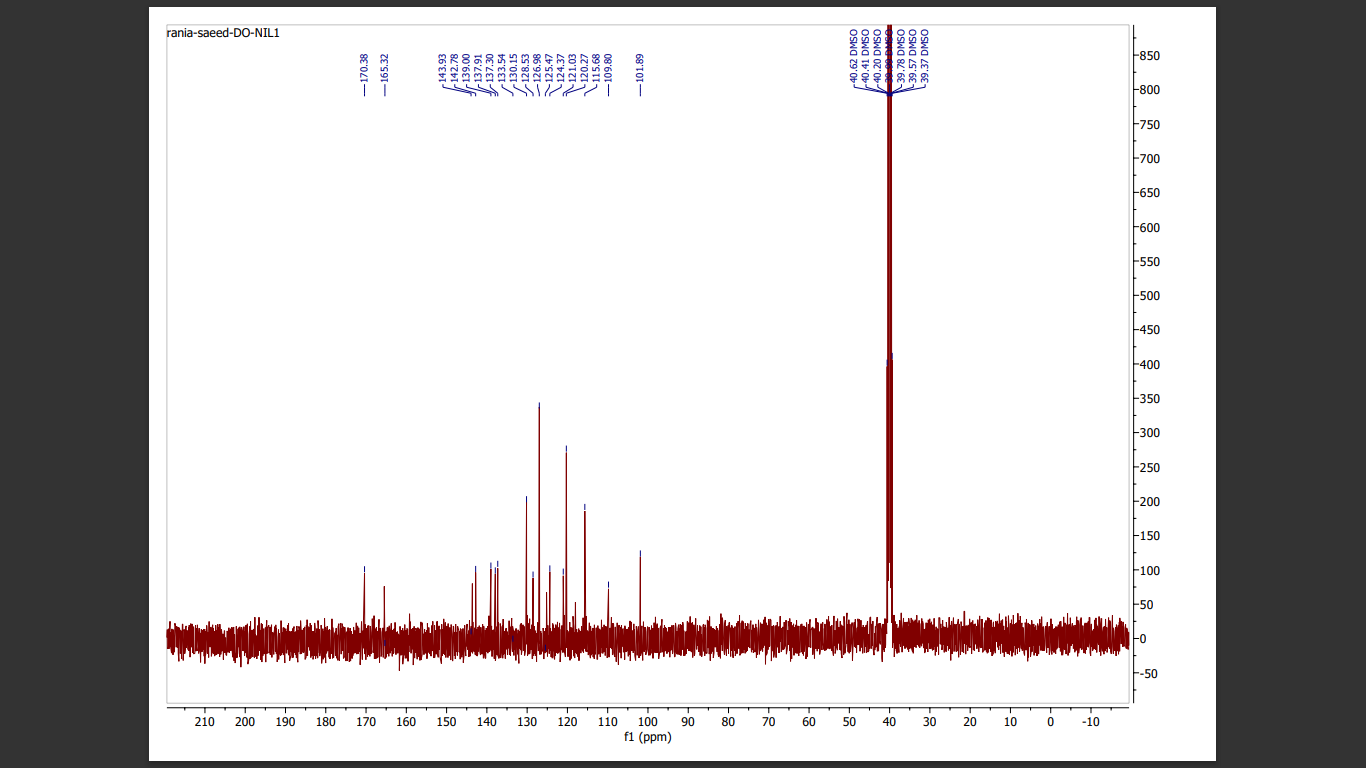

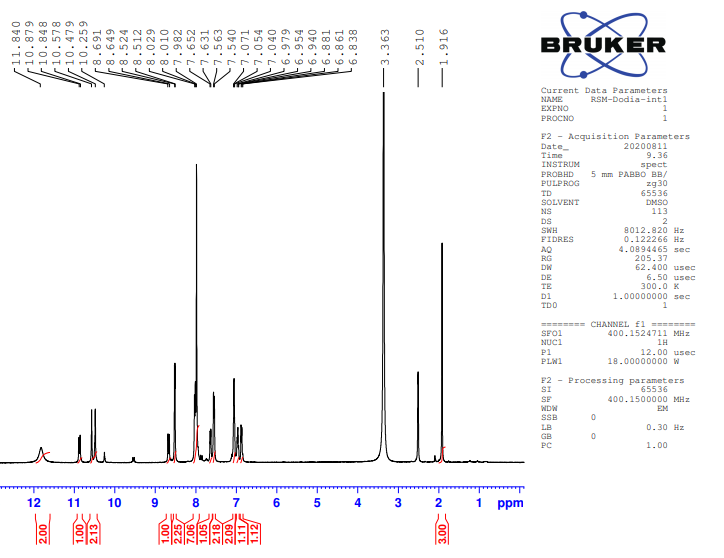

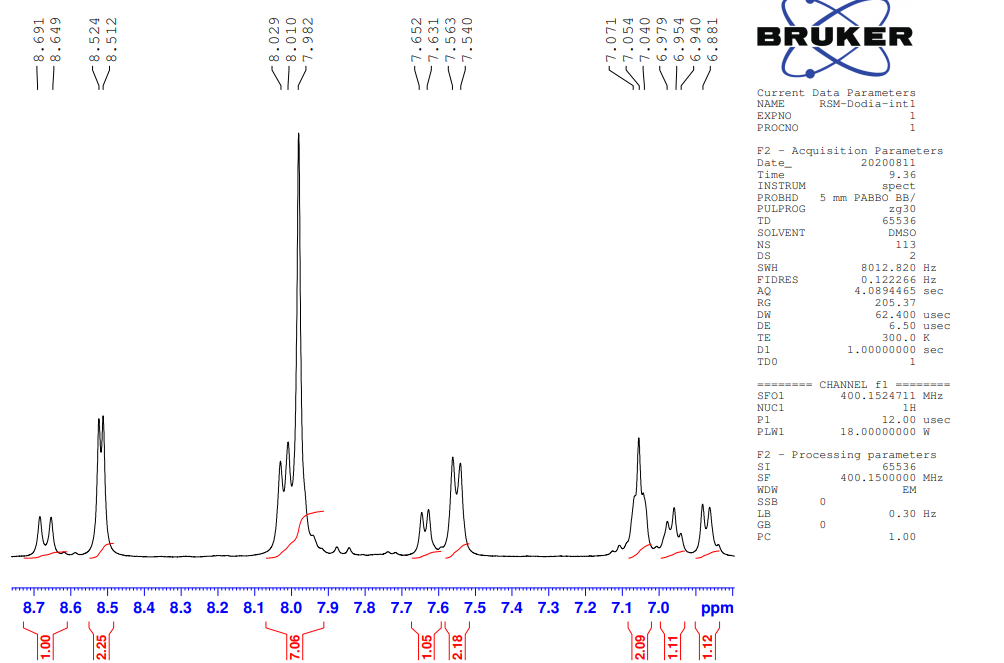

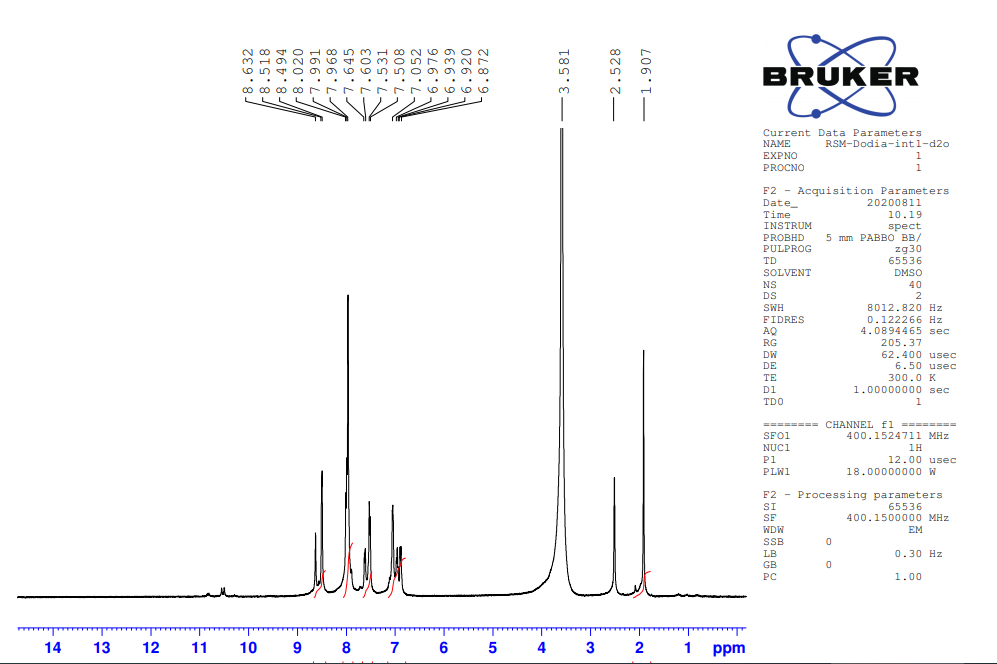


**9b**-d_2_O

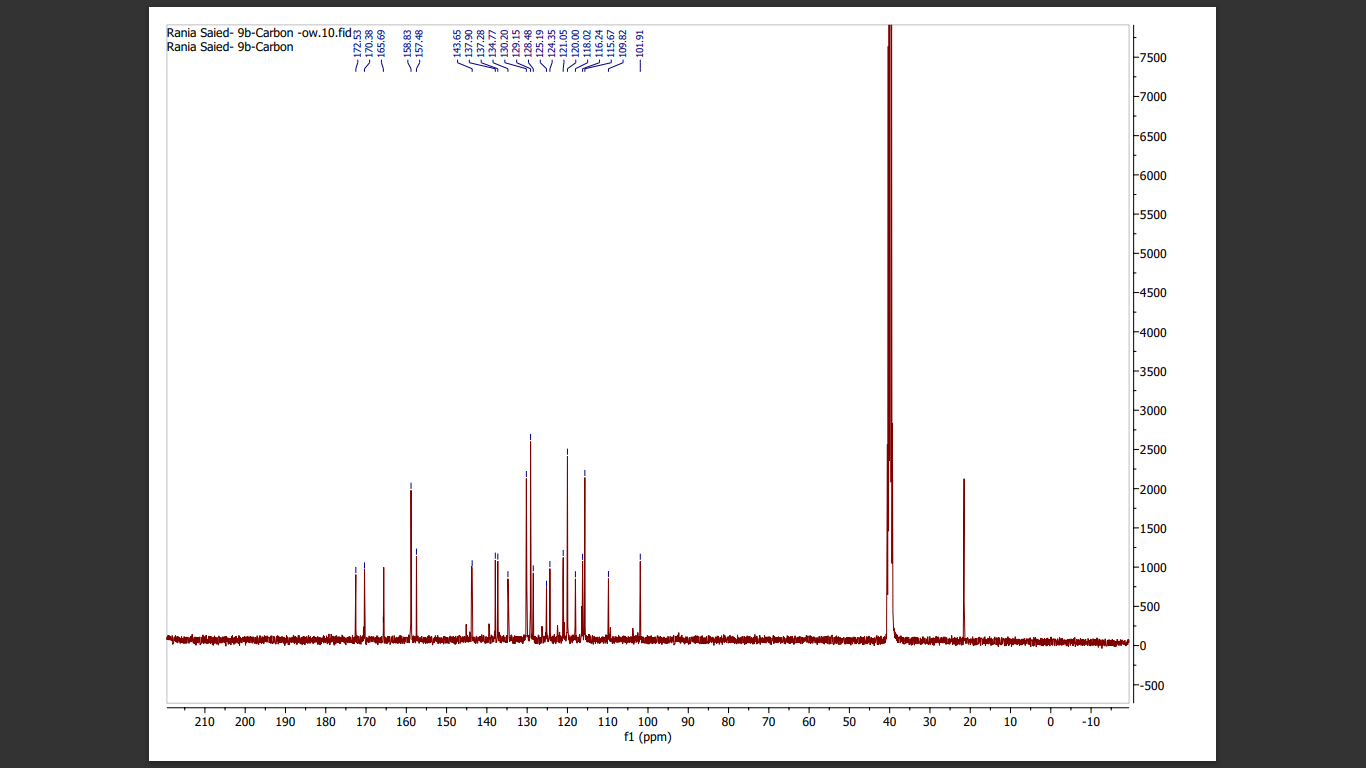


**
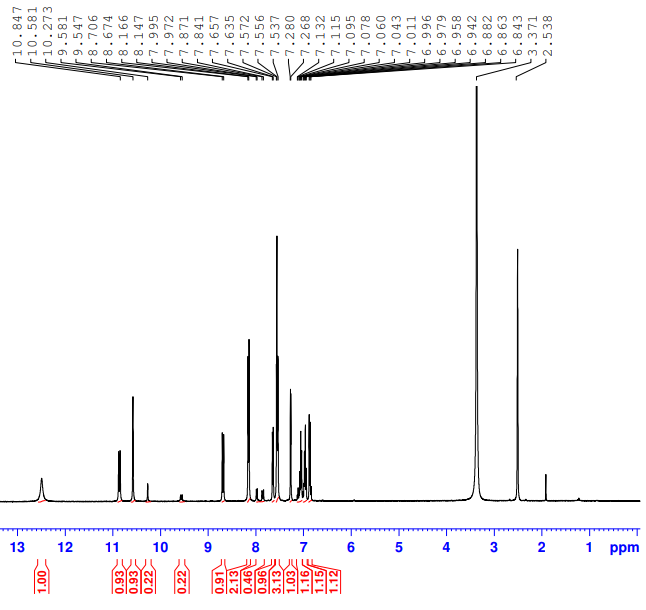

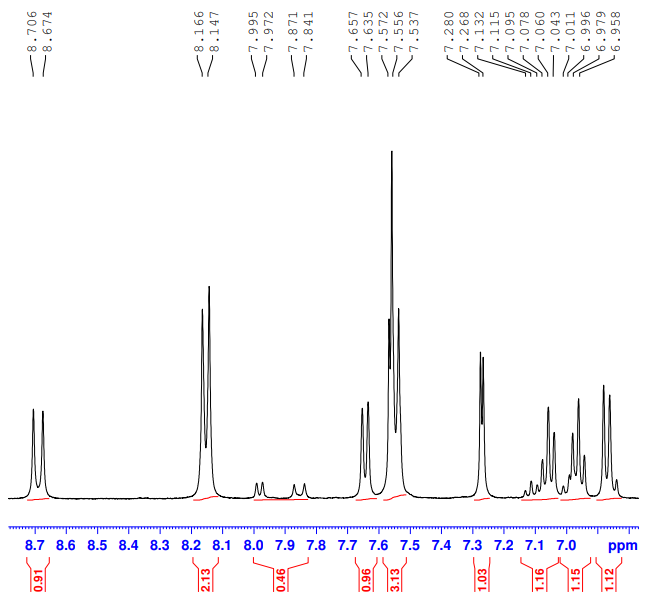
**


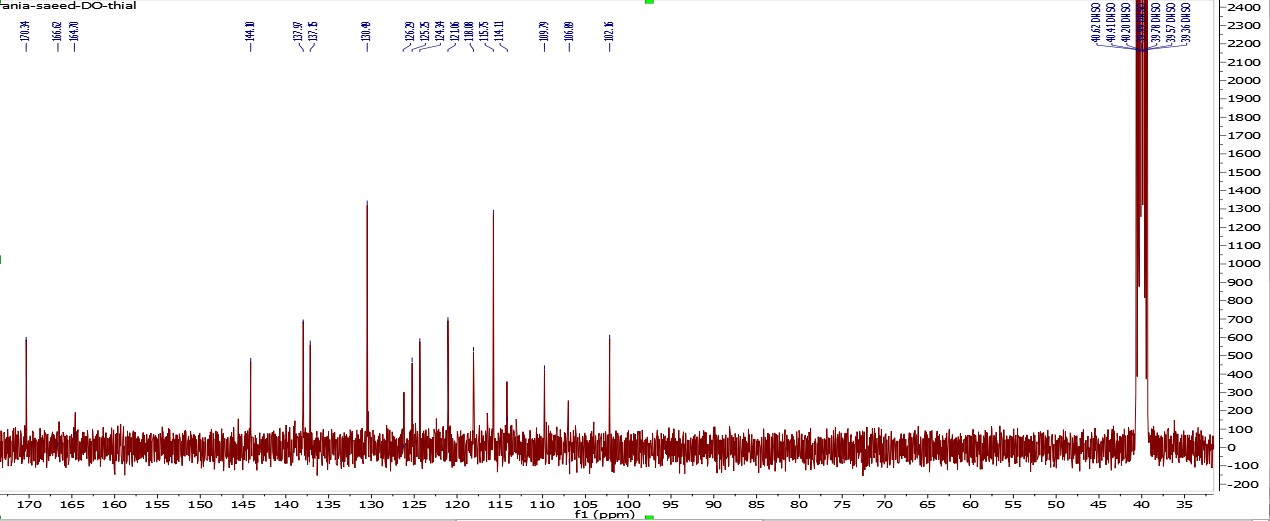

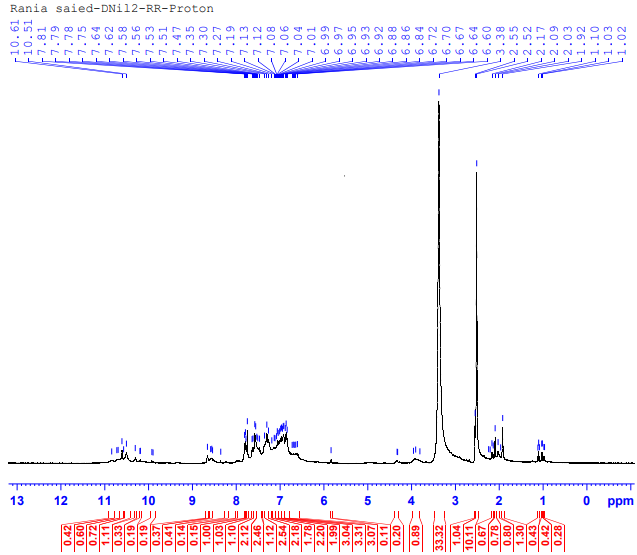

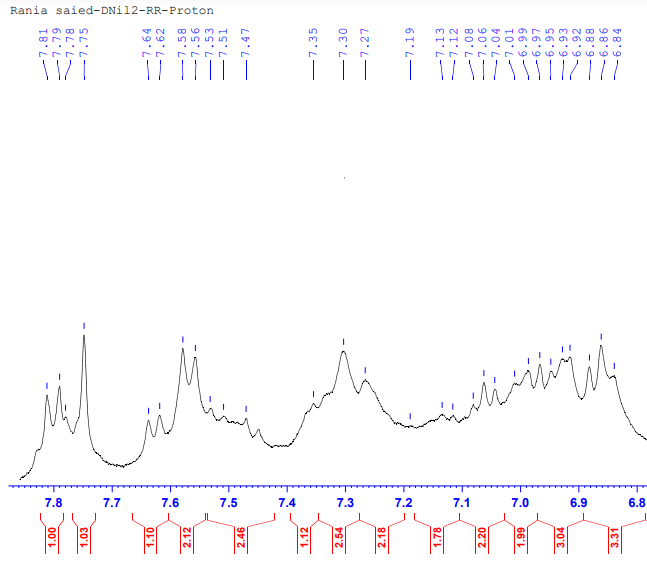


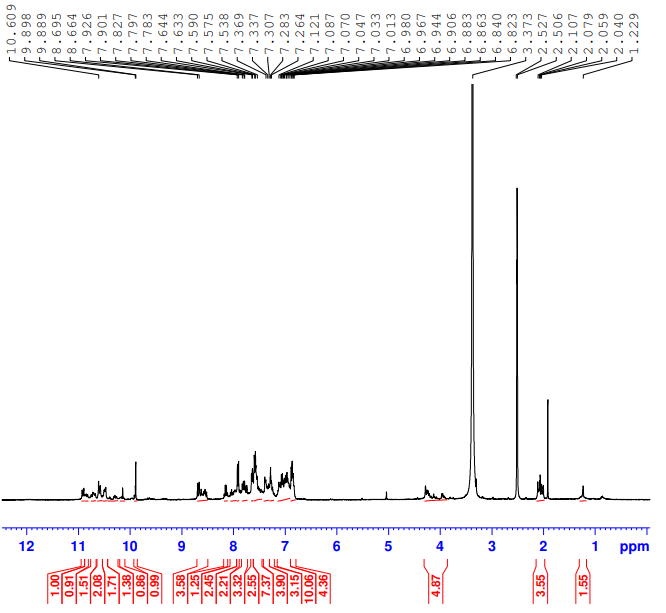


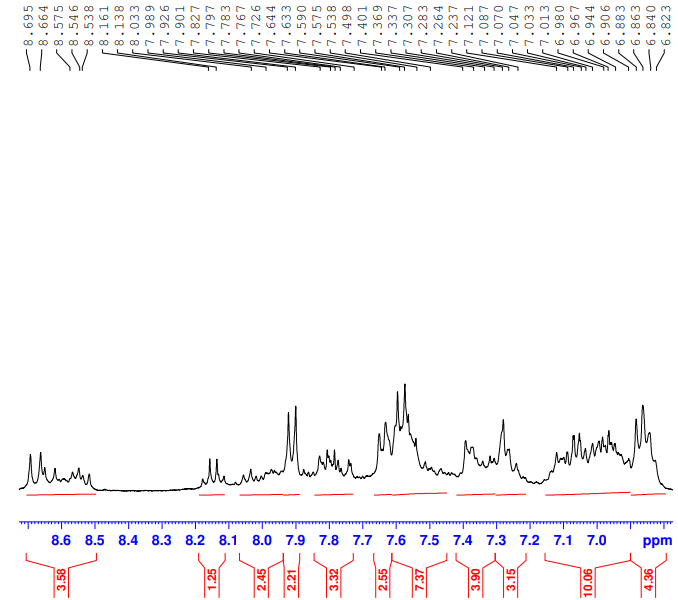

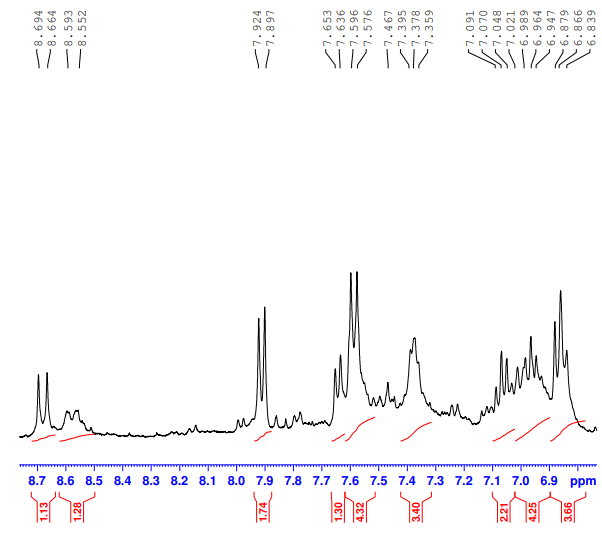

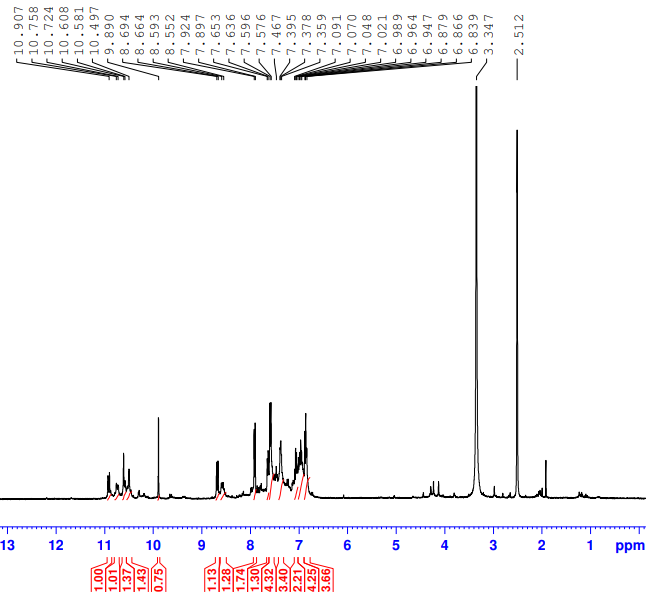

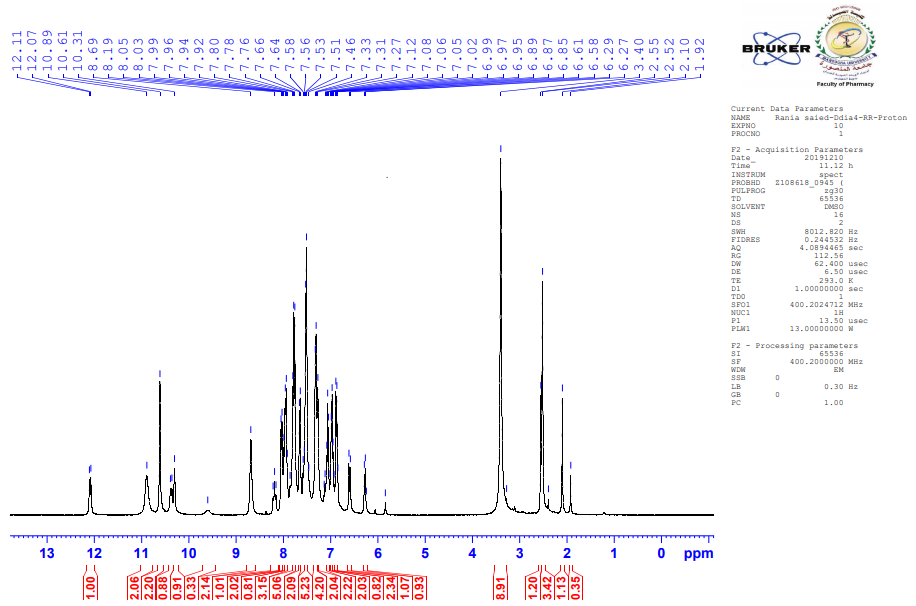

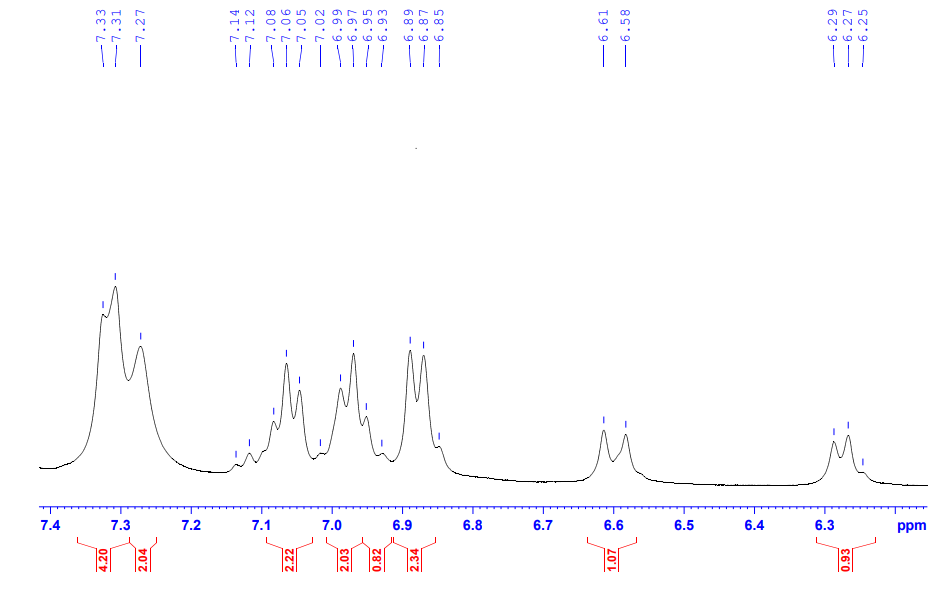

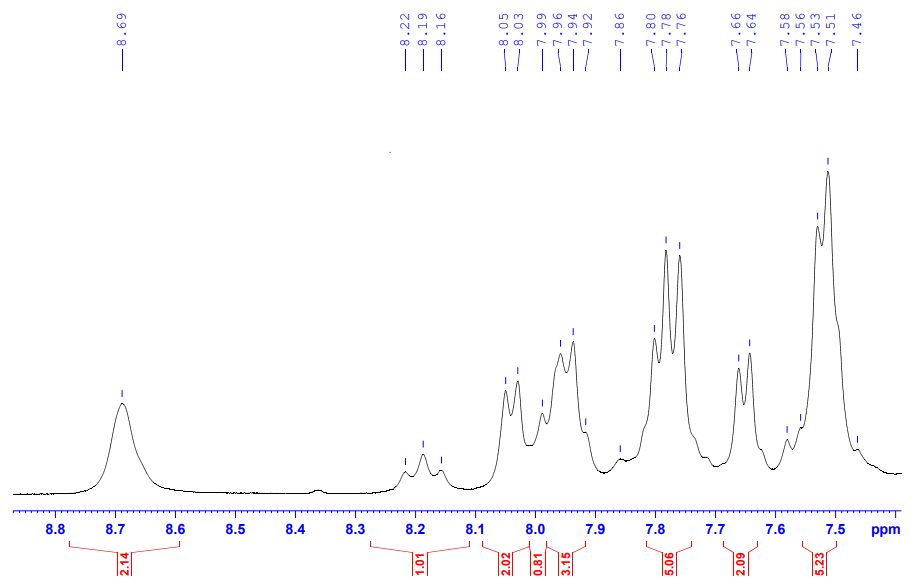


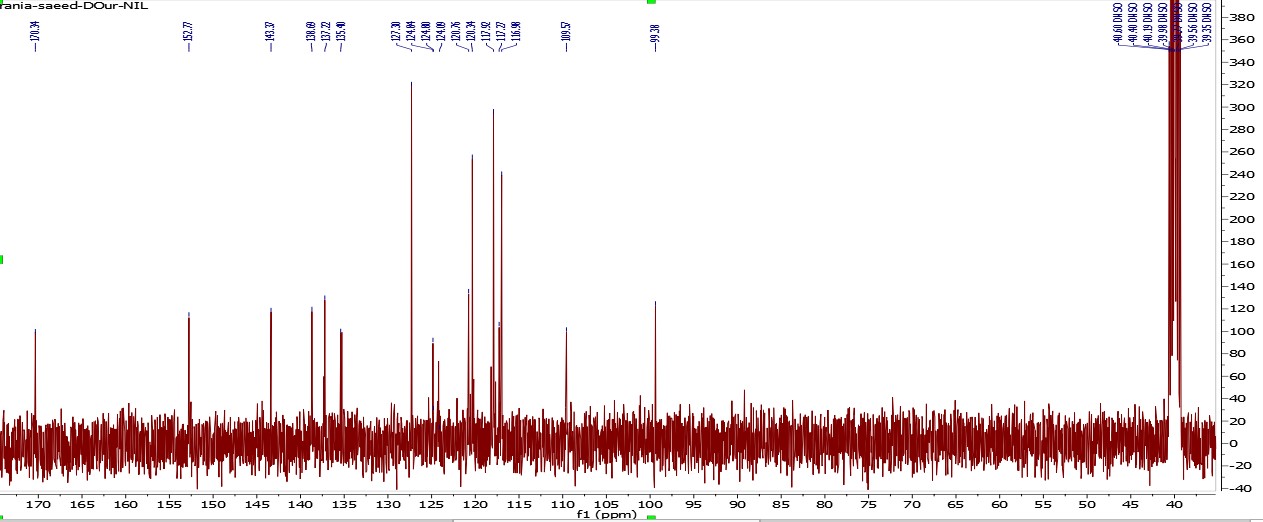

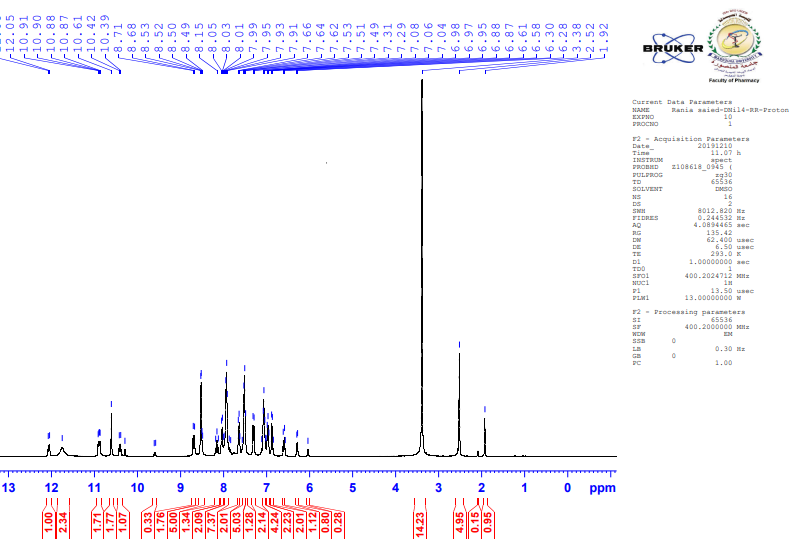

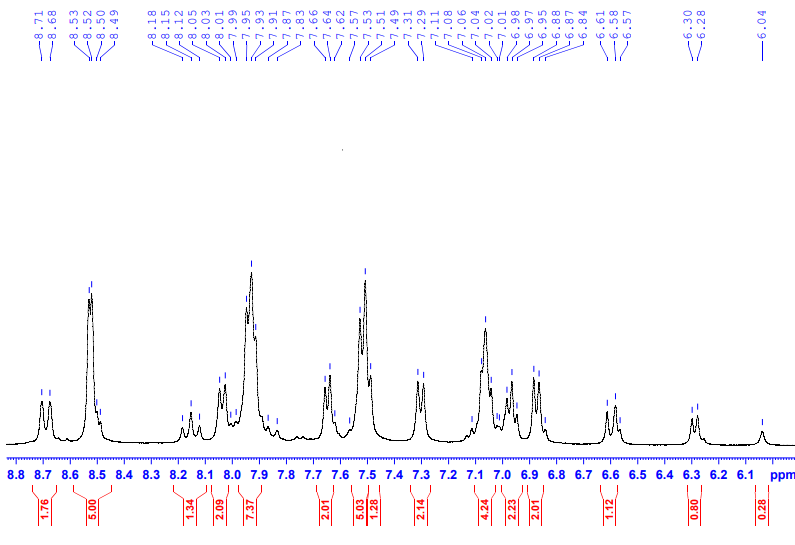

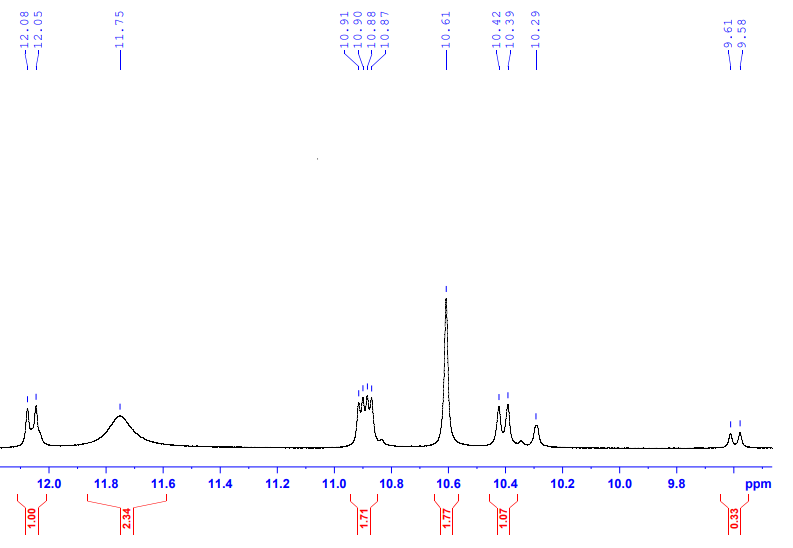

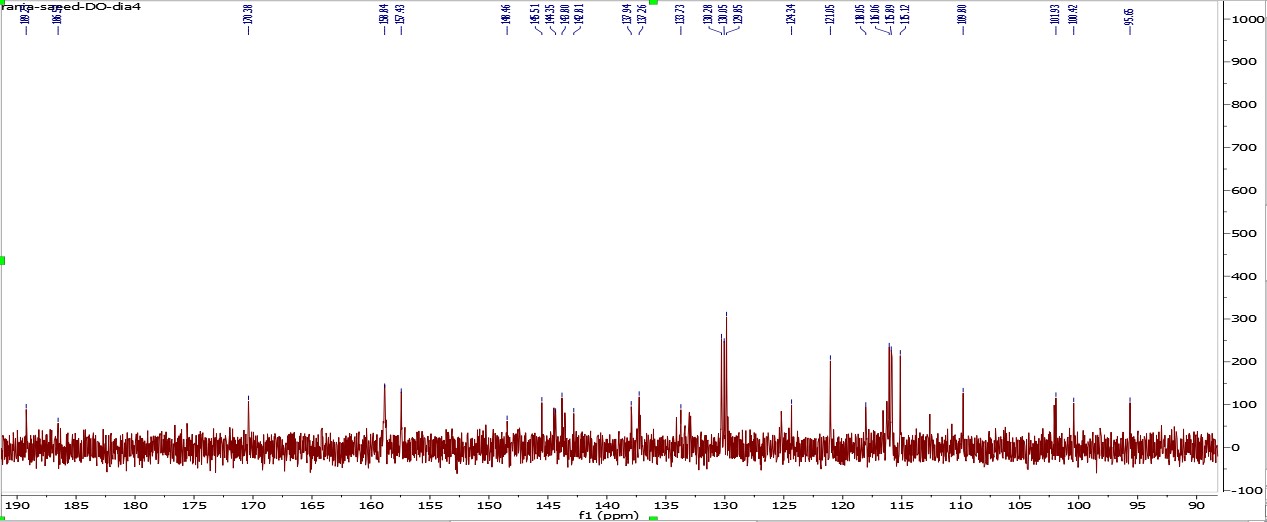

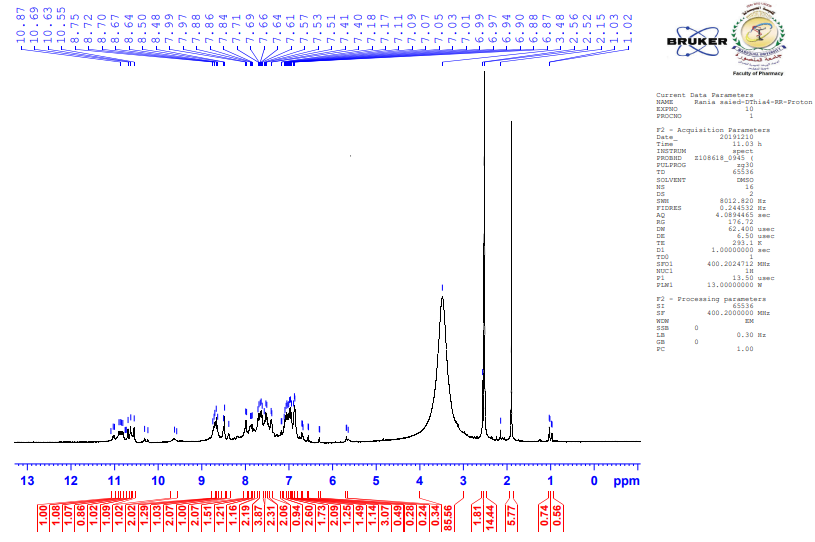


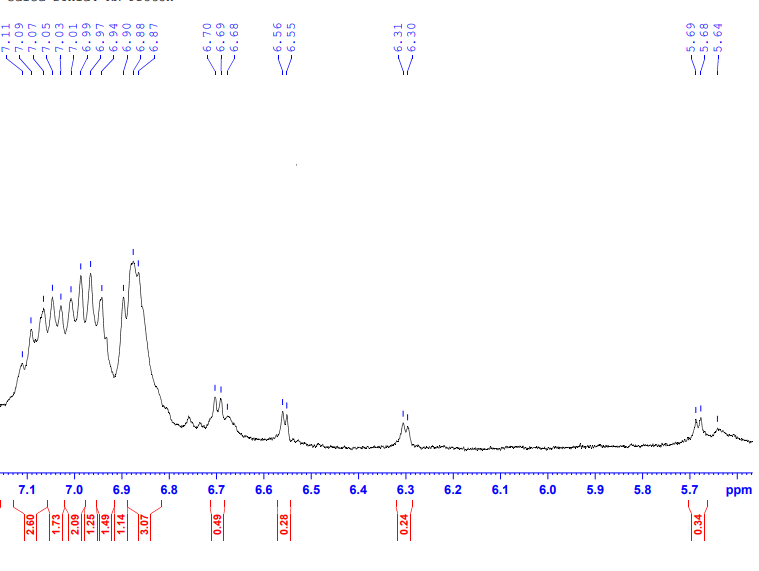

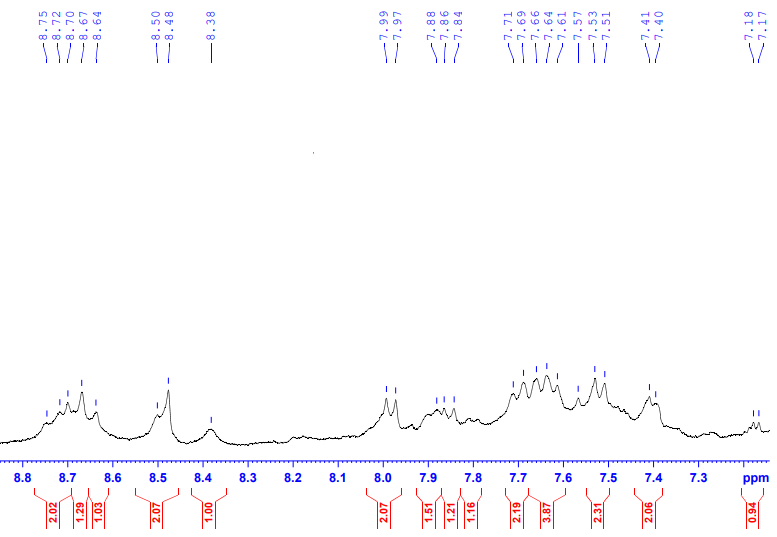

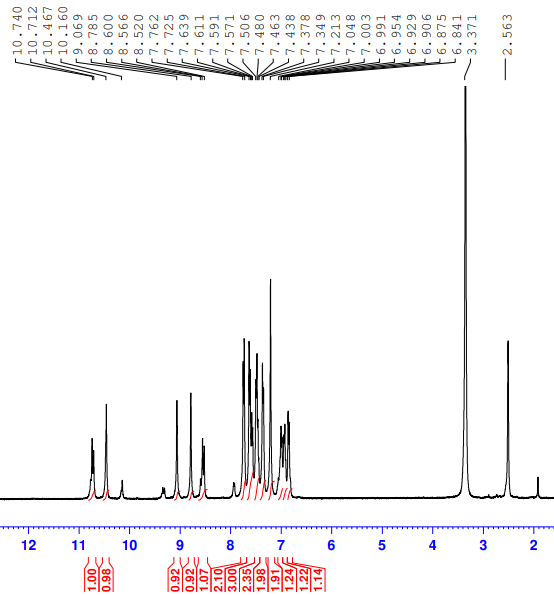


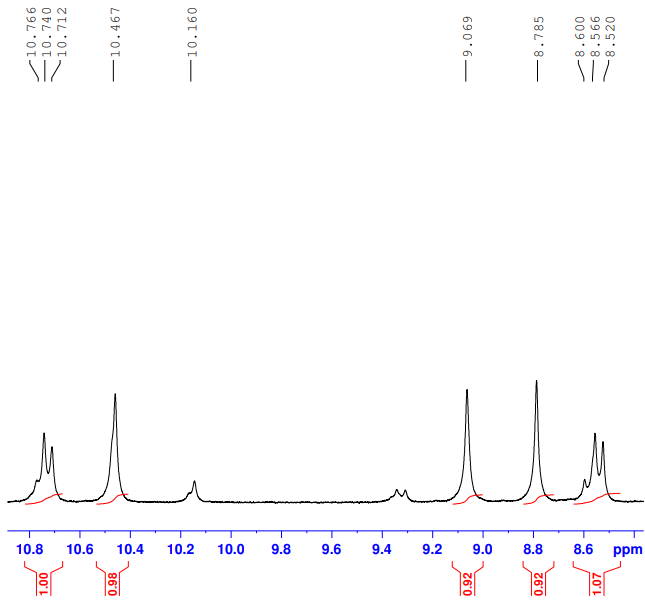


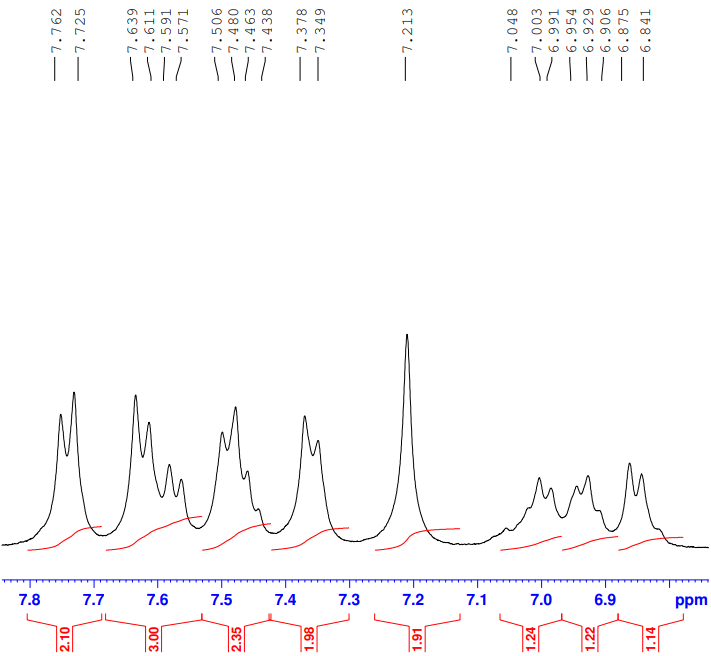


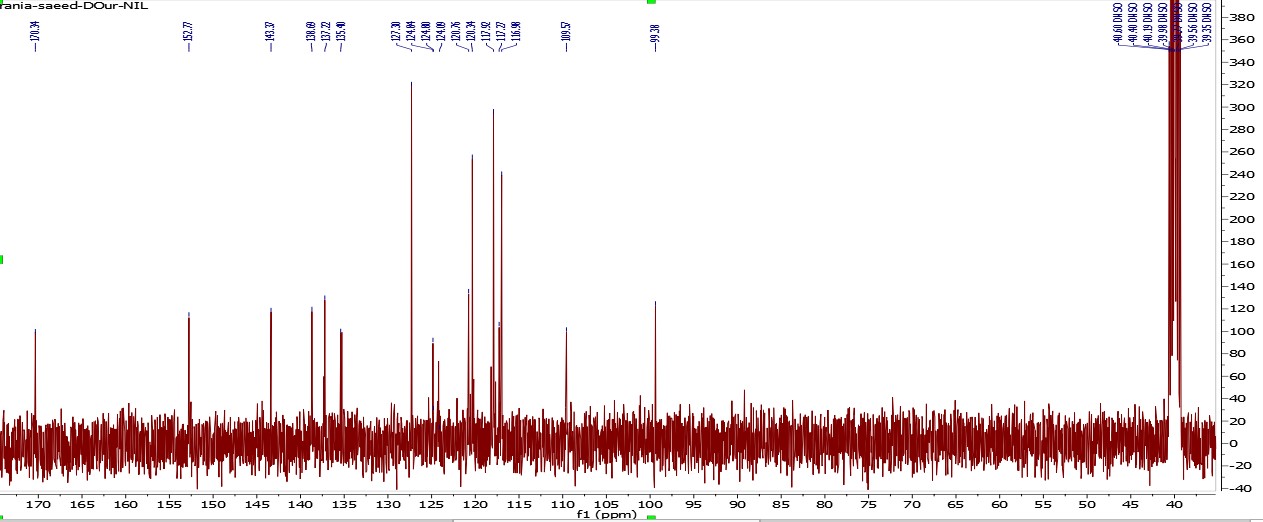

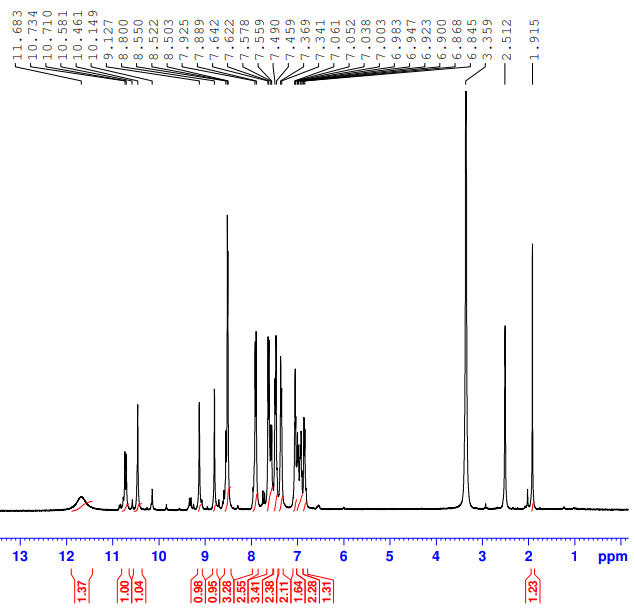

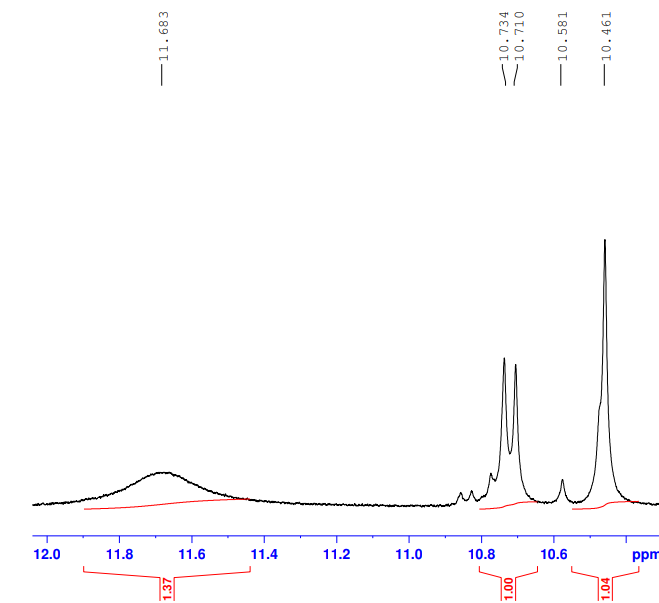

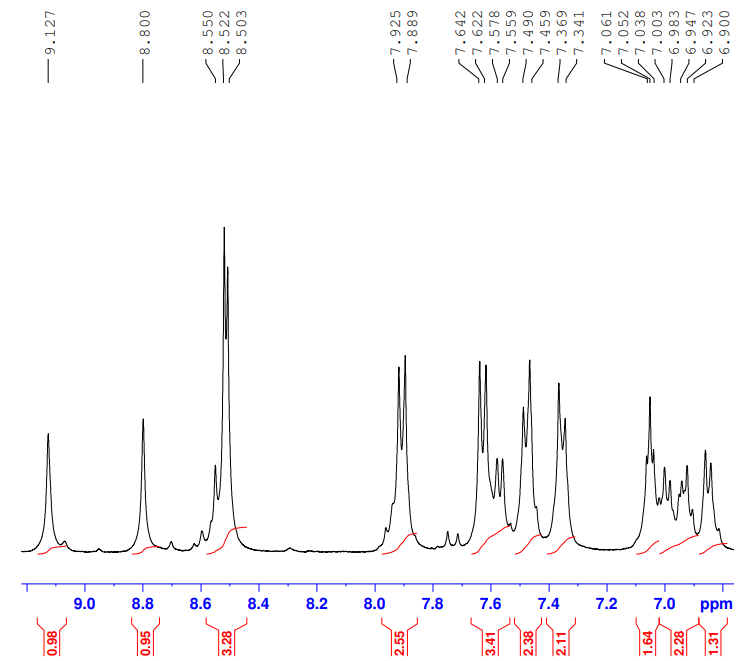

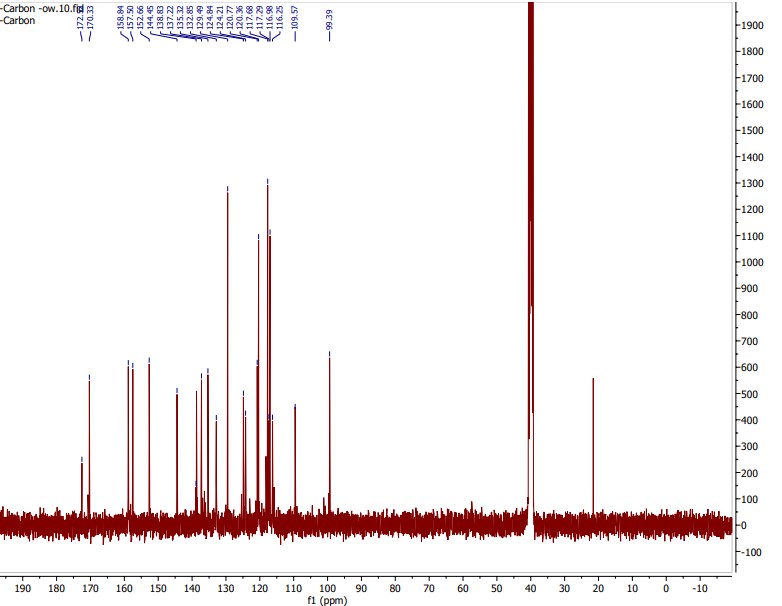

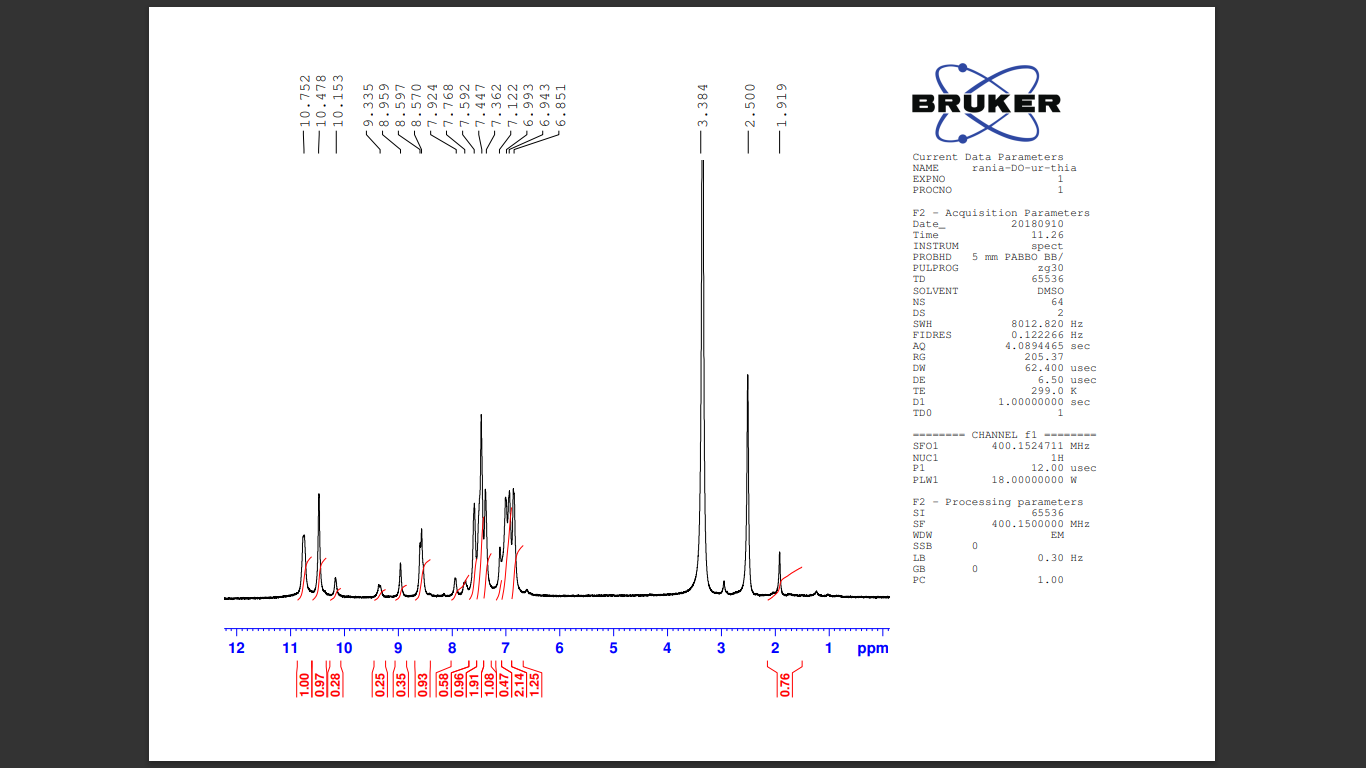

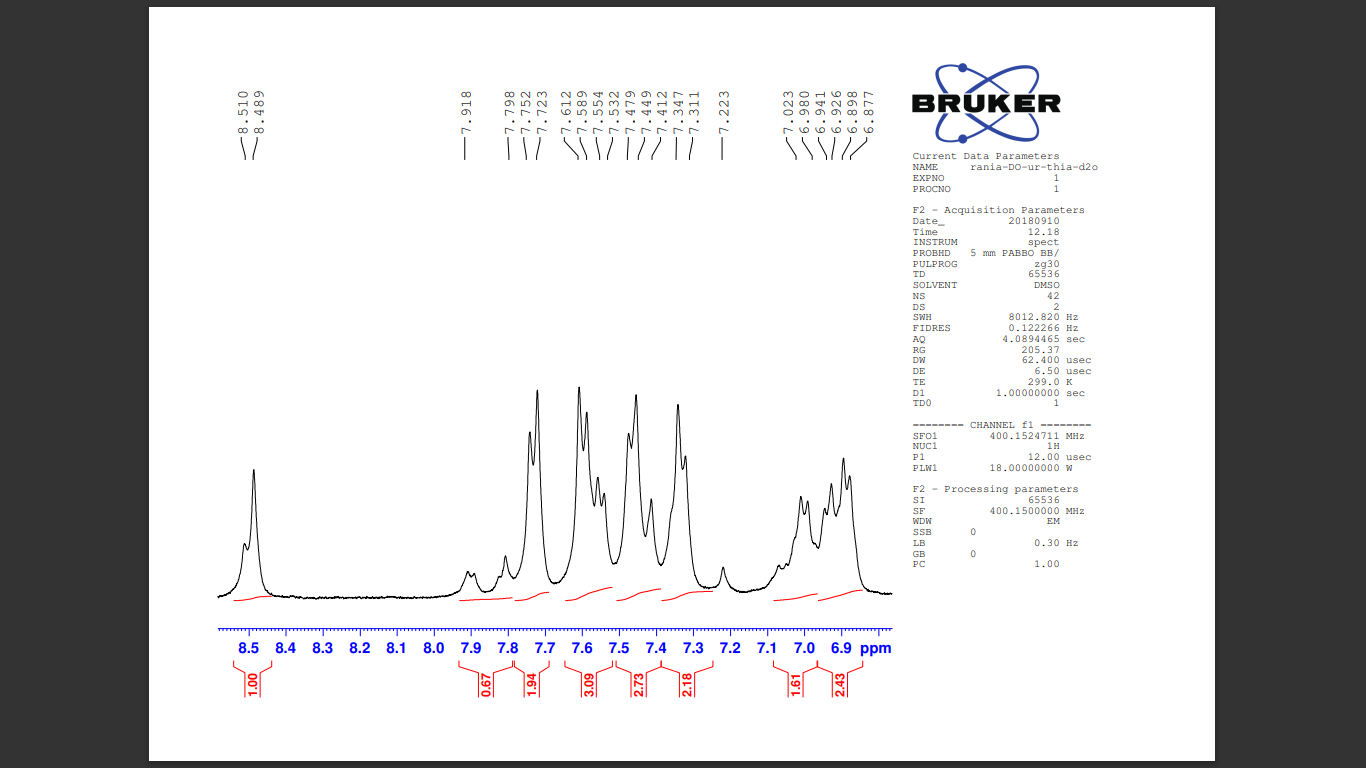

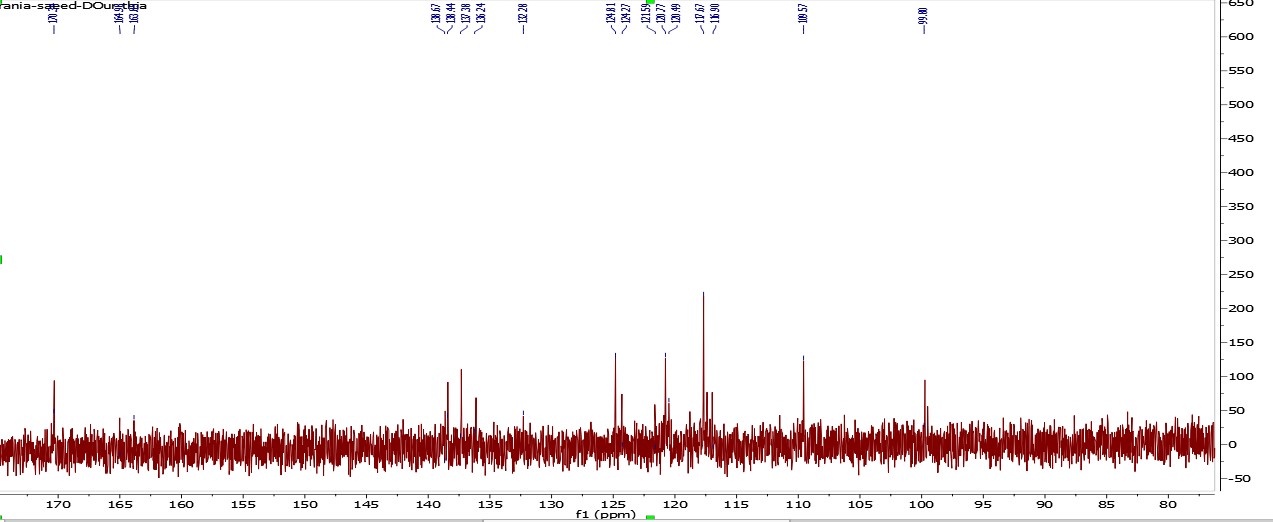

# **S2. Molecular docking study**

All the molecular modeling simulations were carried out using Molecular Operating Environment (MOE, 2020.0901) software. All minimizations were performed with MOE until an RMSD gradient of 0.05 kcal∙mol^−1^Å^−2^ with MMFF94x force field and the partial charges were automatically calculated.

Molecular docking was carried out using X-ray crystallographic structure of CA II, CA IX and CA XII isozymes (PDB ID: 3HS4) ^1^, (PDB ID: 5FL4) ^2^ and (PDB ID: 1JD0) ^3^, respectively. In addition to the X-ray crystallographic structure of VEGFR-2, FGFR1 and RET kinases (PDB ID: 4ASD) ^4^, (PDB ID: 4V01) ^5^, and (PDB ID: 6NEC) ^6^, respectively.

**S2.1. Molecular docking in carbonic anhydrase isozymes (CA II, CA IX and CA XII)**

The X-ray crystallographic structure of CA II, CA IX and CA XII isozymes (PDB ID: 3HS4), (PDB ID: 5FL4), and (PDB ID: 1JD0), respectively, were downloaded from the protein data bank. First, chain A was only kept in case of each protein and water molecules and ligands not involved in binding were removed. Then, the protein structures were prepared for docking study using *Protonate 3D* protocol in MOE with default options. The co-crystalized ligand in each isozyme was used to define the binding site for docking. Triangle Matcher placement method and London dG scoring function were used for docking. Docking setup was first validated by self-docking of the co-crystallized ligand in each protein in the vicinity of the active site of the enzyme.

The self-docking validation step showed the suitability of the used docking protocol for the intended docking study by the small RMSD values (0.867Å, 2.498Å, and 1.756Å in CA II, CA IX and CA XII, respectively) and by the ability of the docking poses of the co-crystalized ligands to reproduce all the key interactions accomplished by the co-crystallized ligand with the hot spots in CA II, CA IX and CA XII active sites (Zn^2+^, Thr199 and/or Thr200) (figures S1-S3). The validated setup was then used to study the binding mode of compounds **4d**, **5**, and **7** in the different carbonic anhydrase isoforms CA II, CA IX and CA XII and to predict their potential affinity to these isoforms. **AAZ** was also docked in the three CA isoforms as a reference standard.


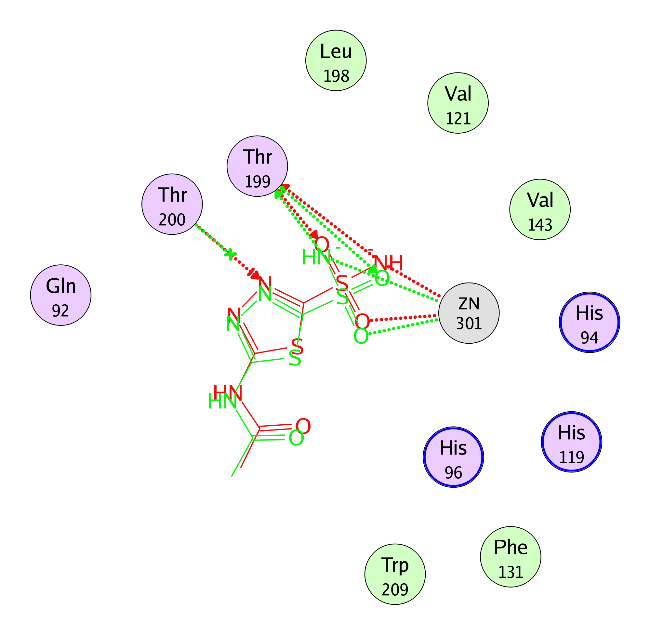


**Figure S1.** Superimposition of the co-crystallized (red) and the docking pose (green) of **AAZ** in CA II active site with RMSD of 0.867 Å.


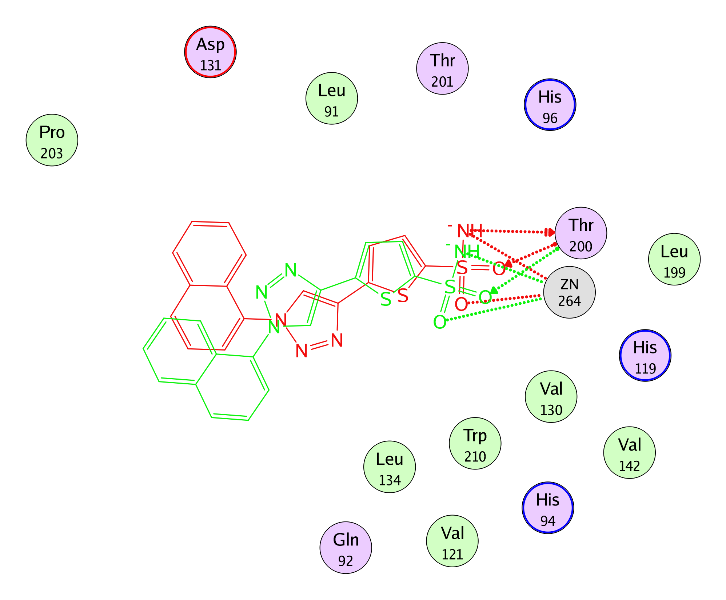


**Figure S2.** Superimposition of the co-crystallized (red) and the docking pose (green) of **9FK** in CA IX active site with RMSD of 2.498 Å.


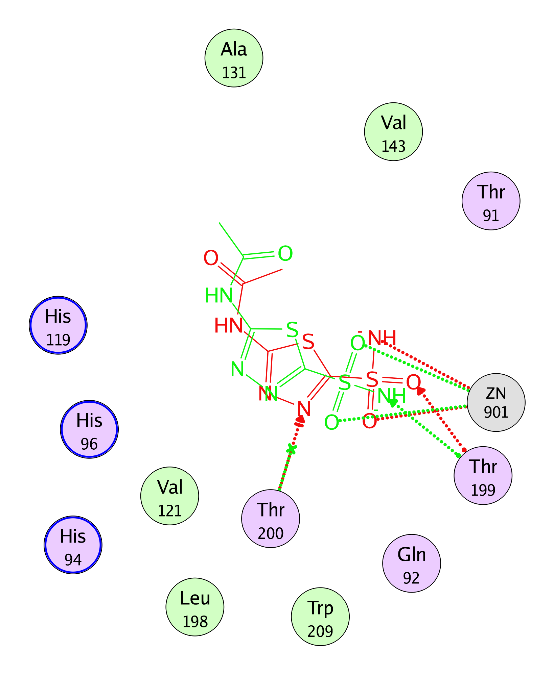


**Figure S3.** Superimposition of the co-crystallized (red) and the docking pose (green) of **AAZ** in CA XII active site with RMSD of 1.756 Å.

**S2.2. Molecular docking in the protein kinases (VEGFR-2, FGFR1 and RET)**

**S2.2.1. VEGFR-2**

The X-ray crystallographic structure of VEGFR-2 co-crystallized with sorafenib (IC_50_ = 90 nM) as inhibitor (PDB ID: 4ASD) was downloaded from the protein data bank. Water molecules were first removed, then the protein was prepared for the docking study using *Protonate 3D* protocol in MOE with default options. The co-crystalized ligand (sorafenib) was used to define the active site for docking. Triangle Matcher placement method and London dG scoring function were used for docking. Docking protocol was first validated by self-docking of the co-crystallized ligand (sorafenib) in the vicinity of the active site of the receptor giving a docking pose with an energy score (S) = −15.19 kcal/mol and an RMSD of 0.470Å from the co-crystalized ligand pose. (Figure S4)


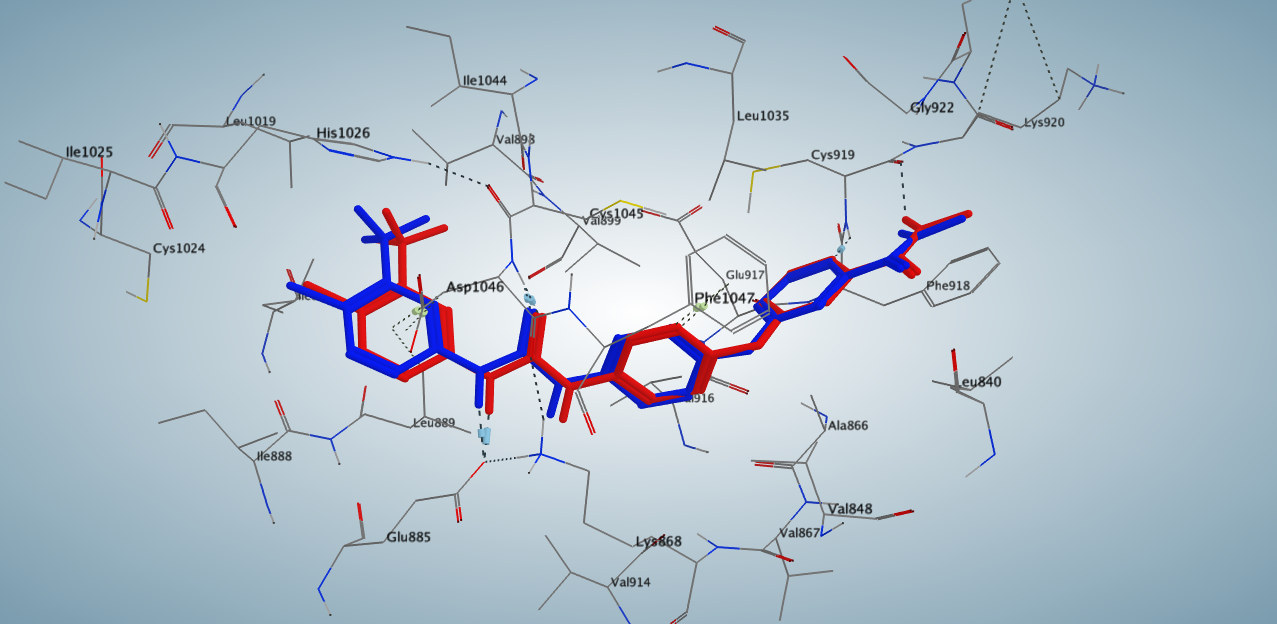


(A)


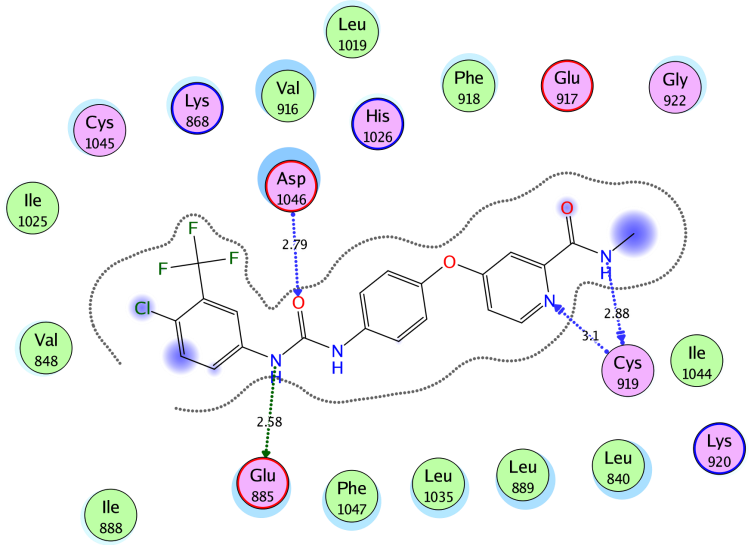


(B)

**Figure S4.** (A) Superimposition of the docking pose (blue) and the co-crystallized (red) of sorafenib in the VEGFR-2 active site with RMSD of 0.470Å. (B) 2D interaction diagram showing sorafenib docking pose interactions with the key amino acids (hot spots) in the VEGFR-2 active site. (Distances in Å)

**S2.2.2. FGFR1**

The X-ray crystallographic structure of FGFR-1 co-crystallized with ponatinib (IC_50_ = 0.7 nM) as inhibitor (PDB ID: 4V01) was downloaded from the protein data bank. Chain A, water molecules and ligands which are not involved in binding were first removed, then the protein was prepared for the docking study using *Protonate 3D* protocol in MOE with default options. The co-crystalized ligand (ponatinib) was used to define the active site for docking. Triangle Matcher placement method and London dG scoring function were used for docking. Docking protocol was first validated by self-docking of the co-crystallized ligand (ponatinib) in the vicinity of the active site of the receptor giving a docking pose with an energy score (S) = −17.00 kcal/mol and an RMSD of 0.398Å from the co-crystalized ligand pose. (Figure S5)


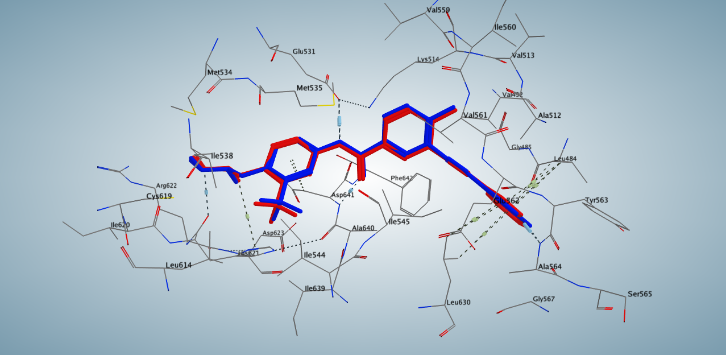


(A)


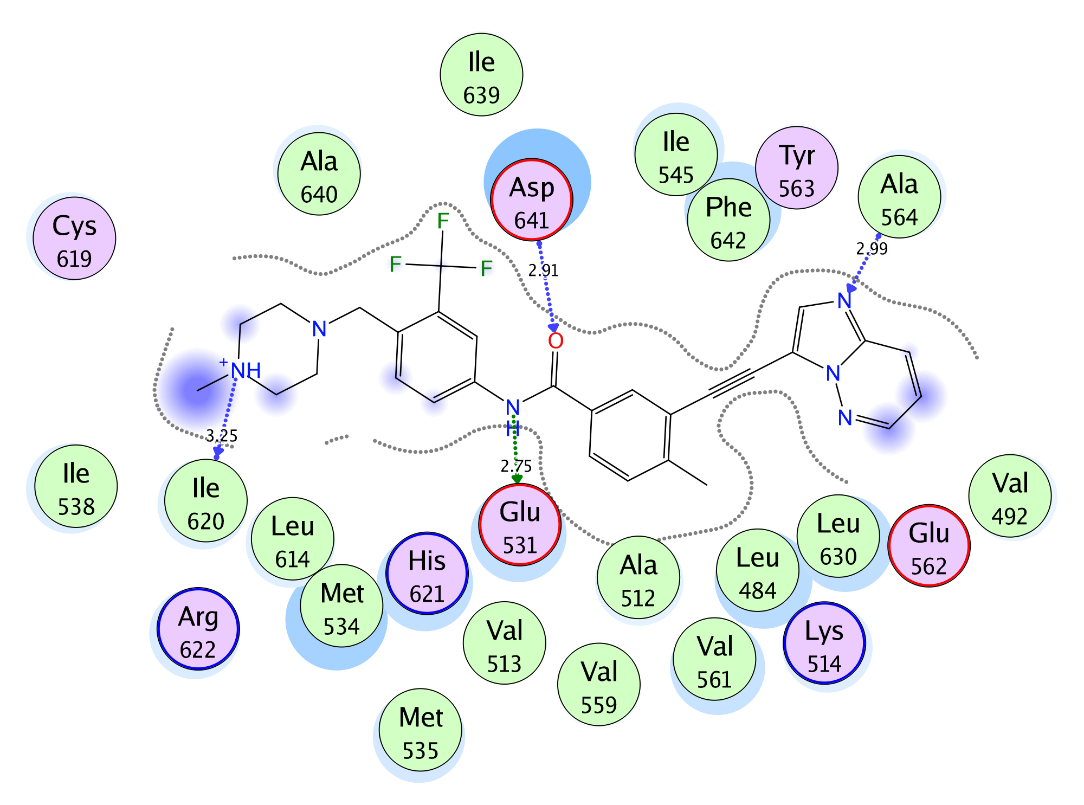


(B)

**Figure S5.** (A) Superimposition of the docking pose (blue) and the co-crystallized (red) of ponatinib in the FGFR-1 active site with RMSD of 0.398Å. (B) 2D interaction diagram showing ponatinib docking pose interactions with the key amino acids (hot spots) in the FGFR-1 active site. (Distances in Å)

**S2.2.3. RET**

Protein data bank (PDB) search showed that there is not any RET kinase protein structure co-crystalized with a type II kinase inhibitor. The available are either apoprotein structures for RET kinase or co-crystalized complexes with type I kinase inhibitors such as Vandetanib. On the other hand, several crystal structures are obtainable from the PDB for other kinases such as VEGFR-2 in the DFG-out inactive conformation that are co-crystalized with type II inhibitors. The overall homology between RET and VEGFR-2 is 42%, suggesting that both proteins are folded in a similar fashion. To define RET kinase type II inhibitors’ binding site and to induce its DFG-out inactive conformation shape and topology, the RET protein structure co-crystalized with nintadinib (PDB ID: 6NEC) was aligned to the VEGFR-2 protein structure co-crystalized with sorafenib as inhibitor (PDB ID: 4ASD). VEGFR-2 protein and the RET inhibitor (nintadinib) were then deleted leaving behind sorafenib in the RET binding site followed by energy minimization of the RET protein structure. The final resulted structure contains RET protein kinase in a DFG-out conformation bound to a type II kinase inhibitor (sorafenib) interacting with the key amino acids Ala807, Glu775, and Asp892 in RET binding site (figure S6).


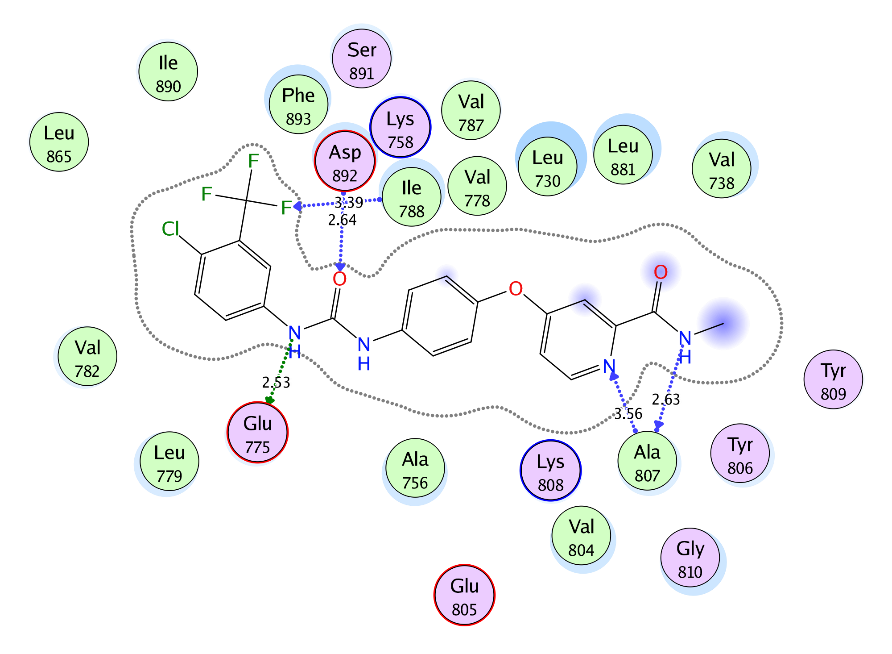


**Figure S6.** 2D diagram of **sorafenib** showing its interaction with RET kinase active site

Chain B, water molecules and ligands which are not involved in binding were first removed, then the protein was prepared for the docking study using *Protonate 3D* protocol in MOE with default options. The added ligand (sorafenib) was used to define the active site for docking. Triangle Matcher placement method and London dG scoring function were used for docking. Docking protocol was first validated by self-docking of the added ligand (sorafenib) in the vicinity of the active site of the receptor giving a docking pose with an energy score (S) = −14.23 kcal/mol and an RMSD of 0.331Å from the added ligand pose. (Figure S7)


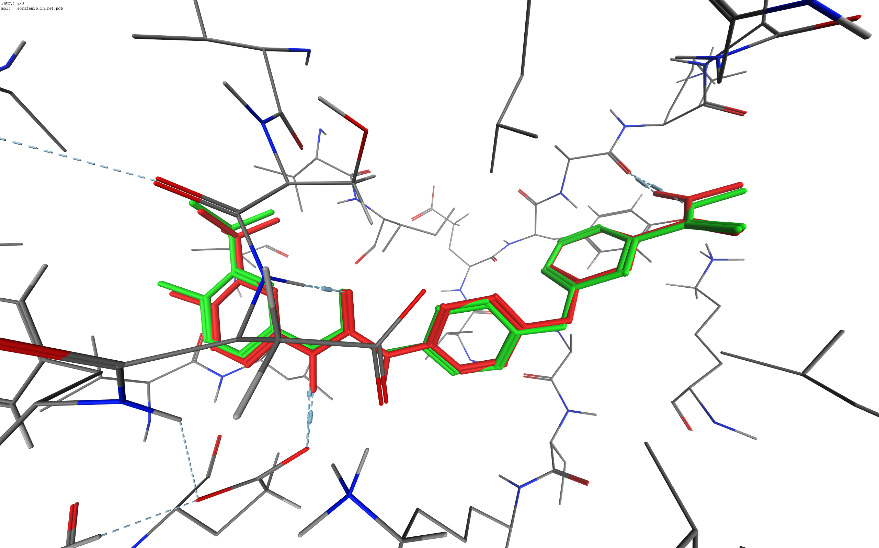


(A)


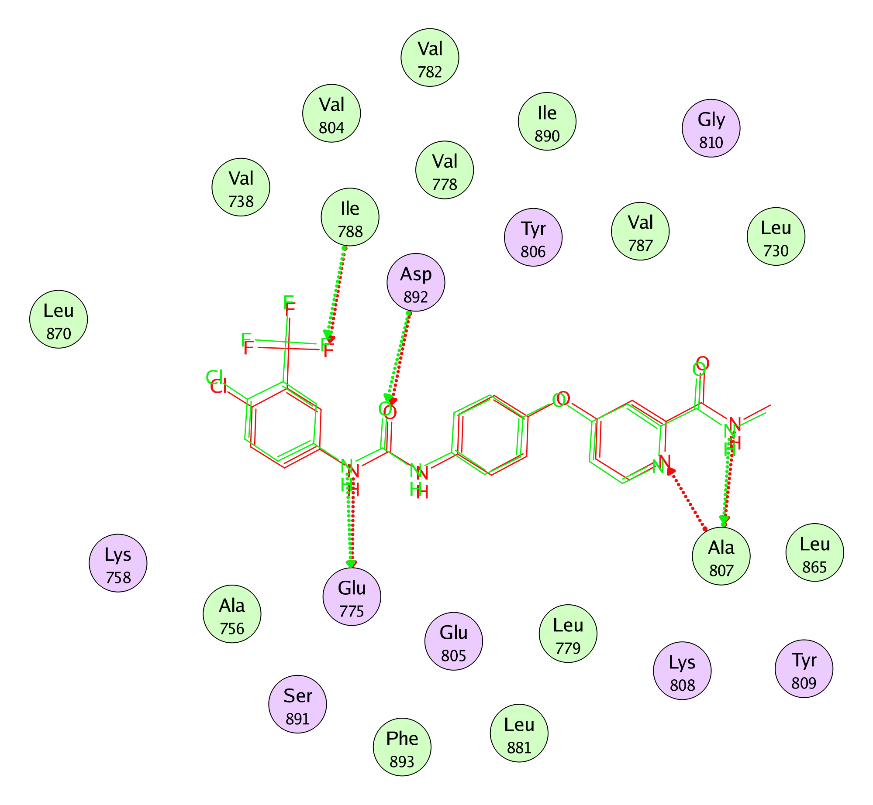


(B)

**Figure S7.** 3D representation (A) and 2D diagram (B) of the superimposition of the docking pose (green) and the co-crystallized (red) of sorafenib in the RET active site with RMSD of 0.331Å.

The validated docking protocols were then used to study the ligand-receptor interactions in the active site of the target kinases for compound **15c** to predict its binding mode and to rationalize its binding affinity.

**S2.3. Docking poses of compounds 5 and 7 in the active site of CA II, CA IX, and CA XII**

**
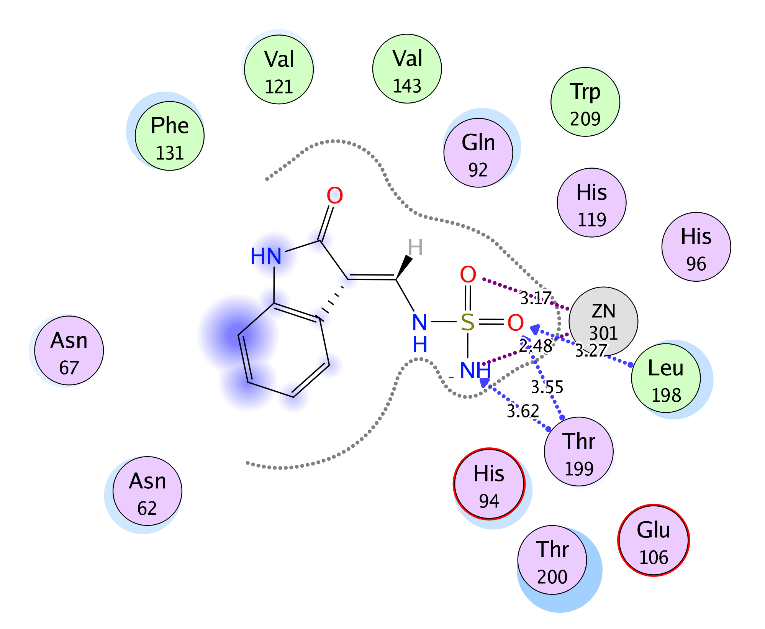
**

**Figure S8.** 2D diagram of compound **5** showing its interaction with CA II active site.

**
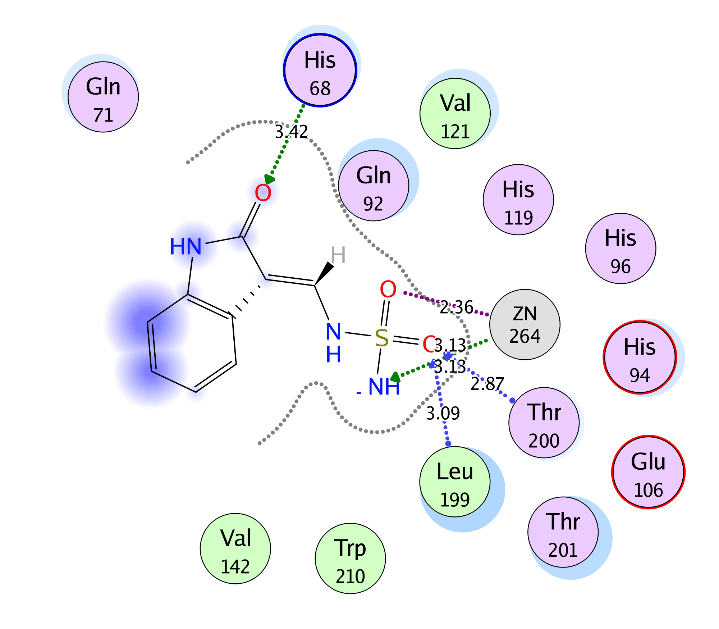
**

**Figure S9.** 2D diagram of compound **5** showing its interaction with CA IX active site.

**
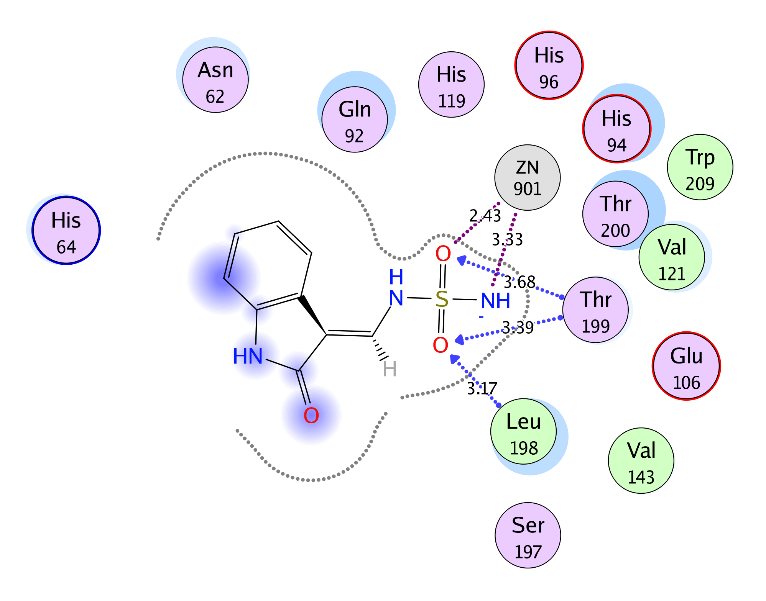
**

**Figure S10.** 2D diagram of compound **5** showing its interaction with CA XII active site.

**
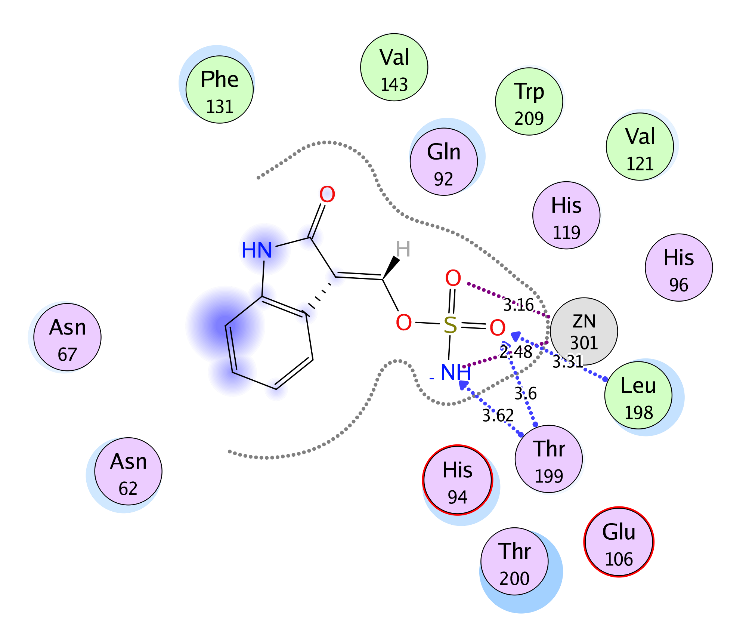
**

**Figure S11.** 2D diagram of compound **7** showing its interaction with CA II active site.

**
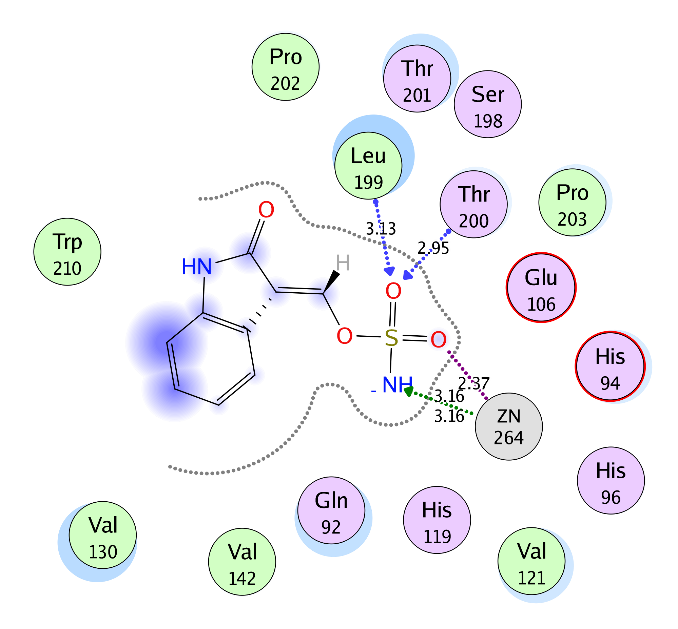
**

**Figure S12.** 2D diagram of compound **7** showing its interaction with CA IX active site.

**
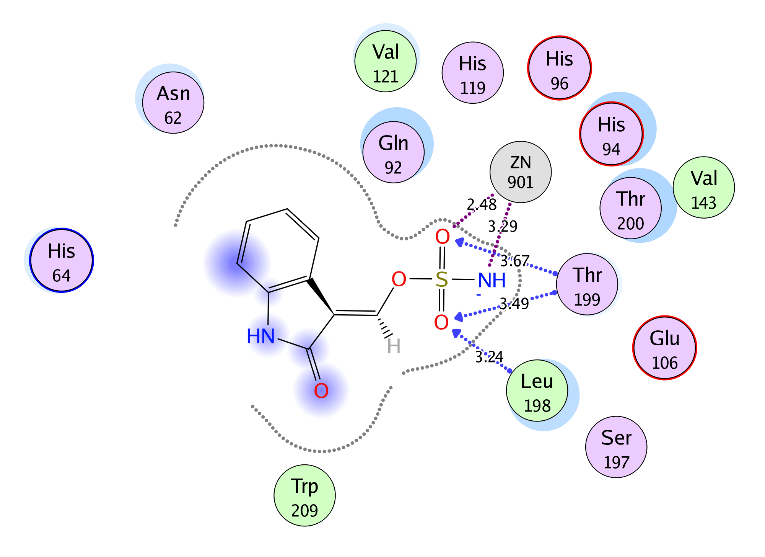
**

**Figure S13.** 2D diagram of compound **7** showing its interaction with CA XII active site.

# **S3. In Vitro biological activity**

**S3.1. In Vitro Antitumor Activity towards 60 cancer cell lines (NCI-USA Anticancer Assay)**

NCI In-vitro Cancer Screening is a two-phase procedure that starts with the assessment of all compounds against the sixty NCI cell line panel depicting leukemia, NSCLC, CNS cancer, melanoma, prostate cancer, breast cancer, renal cancer, colon cancer and ovarian cancer cell lines at a single point of 10μM. The output from the single-dose screen is reportable as a mean graph.

The human tumor cell lines of the cancer screening panel were grown in RPMI 1640 medium containing 5% fetal bovine serum and 2 mM L-glutamine. For a typical screening experiment, cells were inoculated into 96 well microtiter plates in 100 µ at plating densities ranging from 5000 to 40,000 cells/well depending on the doubling time of individual cell lines. After cell inoculation, the microtiter plates were incubated at 37 ^o^C, 5% CO_2_, 95% air and 100% relative humidity for 24 h prior to addition of experimental drugs. After 24 h, two plates of each cell line were fixed in situ with trichloroacetic acid (TCA), to represent a measurement of the cell population for each cell line at the time of drug addition (Tz). Experimental drugs were solubilized in dimethyl sulfoxide at 400-fold the desired final maximum test concentration and stored frozen prior to use. At the time of drug addition, an aliquot of frozen concentrate was thawed and diluted to twice the desired final maximum test concentration with complete medium containing 50 µg/ml gentamicin. Aliquot of 100 µl of the drug dilution was added to the appropriate microtiter wells already containing 100 µl of medium, resulting in the required final drug concentration (10 µM). Triplicate wells were prepared for each individual dose. Following drug addition, the plates were incubated for an additional 48 h at 37 ^o^C, 5% CO_2_, 95% air, and 100% relative humidity. For adherent cells, the assay was terminated by the addition of cold TCA. Cells were fixed in situ by the gentle addition of 50 µl of cold 50% (w/v) TCA (final concentration, 10% TCA) and incubated for 60 min at 4 oC. The supernatant was discarded, and the plates were washed five times with tap water and air dried. Sulforhodamine B (SRB) solution (100 µl) at 0.4% (w/v) in 1% acetic acid was added to each well, and plates were incubated for 10 min at room temperature. After staining, unbound dye was removed by washing five times with 1% acetic acid and the plates were air dried. Bound stain was subsequently solubilized with 10 mM trizma base, and the absorbance was read on an automated plate reader at a wavelength of 515 nm. For suspension cells, the methodology was the same except that the assay was terminated by fixing settled cells at the bottom of the wells by gently adding 50 µl of 80% TCA (final concentration, 16% TCA). Using the seven absorbance measurements [time zero (Tz), control growth (C), and test growth in the presence of drug (Ti)], the percentage growth 5 was calculated at the drug concentration level. Percentage growth inhibition was calculated as:

[(Ti – Tz) / (C – Tz)] x 100 for concentrations for which Ti ≥ Tz

[(Ti – Tz) / Tz] x 100 for concentrations for which Ti < Tz

**Figure S14.** Mean growth inhibition percent (Mean GI%) of the tested compounds over NCI-60 cell line panel.

**S3.2. In vitro five-dose assay on selected cell lines (MCT-7, HCT-116, and DU 145)**

- **Cell culture:**

MCF-7: Breast Adenocarcinoma, DU-145: Prostate cancer, and HCT-116: Colorectal Cancer were obtained from Nawah Scientific Inc., (Mokatam, Cairo, Egypt). Cells were maintained in DMEM media supplemented with 100 mg/mL of streptomycin, 100 units/mL of penicillin and 10% of heat-inactivated fetal bovine serum in humidified, 5% (v/v) CO_2_ atmosphere at 37°C

- **Cytotoxicity assay**

Cell viability was assessed by SRB assay. Aliquots of 100 μL cell suspension (5x10^3 cells) were in 96-well plates and incubated in complete media for 24 h. Cells were treated with another aliquot of 100 μL media containing drugs at various concentrations. After 72 h of drug exposure, cells were fixed by replacing media with 150 μL of 10% TCA and incubated at 4 °C for 1 h. The TCA solution was removed, and the cells were washed 5 times with distilled water. Aliquots of 70 μL SRB solution (0.4% w/v) were added and incubated in a dark place at room temperature for 10 min. Plates were washed 3 times with 1% acetic acid and allowed to air-dry overnight. Then, 150 μL of TRIS (10 mM) was added to dissolve protein-bound SRB stain; the absorbance was measured at 540 nm using a BMG LABTECH®- FLUOstar Omega microplate reader (Ortenberg, Germany)^7,8^.

**
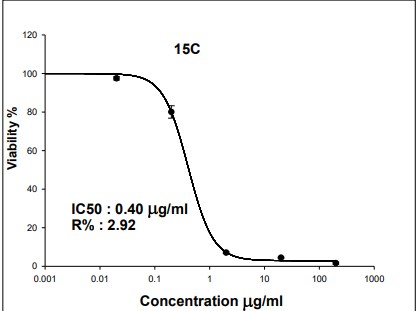

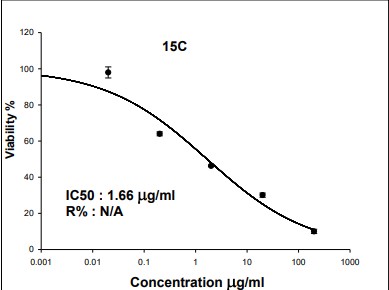
 MCF-7 DU 145**

**
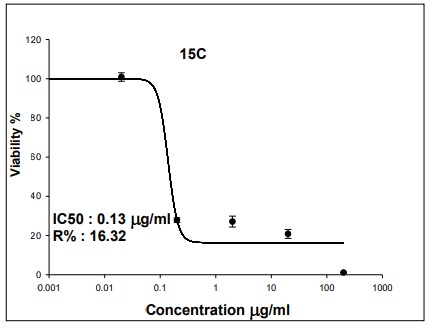
 HCT-116**

**Figure S15.** Dose response curve (IC_50_) of the targeted compound **15c** on MCF-7, DU 145, and HCT-116 cell lines.

**S3.3. Carbonic anhydrase inhibition assay**

The carbonic anhydrase catalyzed CO_2_ hydration actions for all coumarin-based derivatives reported in this study have assayed utilizing an instrument of Applied Photophysics stoppedflow. The enzymes are recombinant proteins prepared in our lab. Phenol red (at a concentration of 0.2 mM) has been used as indicator, working at the absorbance maximum of 557 nm, with 20 mM Hepes (pH 7.5) as buffer, and 20 mM Na_2_SO_4_ (for maintaining constant the ionic strength), following the initial rates of the CA-catalyzed CO_2_ hydration reaction for a period of 10-100 s. The CO2 concentrations ranged from 1.7 to 17 mM for the determination of the kinetic parameters and inhibition constants. For each inhibitor at least six traces of the initial 5-10% of the reaction have been used for determining the initial velocity. The uncatalyzed rates were determined in the same manner and subtracted from the total observed rates. Stock solutions of inhibitor (0.1 mM) were prepared in distilled-deionized water and dilutions up to 0.01 nM were done thereafter with the assay buffer. Inhibitor and enzyme solutions were preincubated together for 6 hours at room temperature prior to assay, in order to allow for the formation of the E-I complex. The inhibition constants were obtained by non-linear least-squares methods using PRISM 3 and the Cheng-Prusoff equation, and represent the mean from at least three different determinations.

**S3.4. kinase inhibition assay**

The FGFR, VEGFR-2 and RET tyrosine kinase assays were performed at Thermo Fischer Scientific, USA (www.thermofischer.com/selectscreen). The assay was performed to evaluate the inhibitory activity of the designed compound applying the Z'-LYTE biochemical assay employing a fluorescence-based, coupled-enzyme format based on the differential sensitivity of phosphorylate and non-phosphorylate peptides to the proteolytic cleavage. The peptide substrate is characterized with two fluorophores, one at each end, that make up a FRET pair ^9^.

The Z´-LYTE biochemical assay employs a fluorescence-based, coupled-enzyme format and is based on the differential sensitivity of phosphorylated and non-phosphorylated peptides to proteolytic cleavage. The peptide substrate is labeled with two fluorophores—one at each end—that make up a FRET pair. In the primary reaction, the kinase transfers the gamma-phosphate of ATP to a single tyrosine, serine or threonine residue in a synthetic FRET-peptide. In the secondary reaction, a site-specific protease recognizes and cleaves non-phosphorylated FRET-peptides. Phosphorylation of FRET-peptides suppresses cleavage by the Development Reagent. Cleavage disrupts FRET between the donor (i.e., coumarin) and acceptor (i.e., fluorescein) fluorophores on the FRET-peptide, whereas uncleaved, phosphorylated FRET-peptides maintain FRET. A ratiometric method, which calculates the ratio (the Emission Ratio) of donor emission to acceptor emission after excitation of the donor fluorophore at 400 nm, is used to quantitate reaction progress, as shown in the equation below. A significant benefit of this ratiometric method for quantitating reaction progress is the elimination of wellto-well variations in FRET-peptide concentration and signal intensities. As a result, the assay yields very high Z´-factor values (>0.7) at a low percent phosphorylation. Both cleaved and uncleaved FRET-peptides contribute to the fluorescence signals and therefore to the Emission Ratio. The extent of phosphorylation of the FRET-peptide can be calculated from the Emission Ratio. The Emission Ratio will remain low if the FRET-peptide is phosphorylated (i.e., no kinase inhibition) and will be high if the FRET-peptide is non-phosphorylated (i.e., kinase inhibition).

Test Compounds the Test Compounds are screened in 1% DMSO (final) in the well. For 10 point titrations, 3-fold serial dilutions are conducted from the starting concentration of the customer’s choosing. Peptide/Kinase Mixtures All Peptide/Kinase Mixtures are diluted to a 2X working concentration in the appropriate Kinase Buffer (see section Kinase Specific Assay Conditions for a complete description). ATP Solution All ATP Solutions are diluted to a 4X working concentration in Kinase Buffer (50 mM HEPES pH 7.5, 0.01% BRIJ-35, 10 mM MgCl_2_, 1 mM EGTA). ATP Km apparent is previously determined using a Z´-LYTE assay.

**Table S1**. Kinase ATP Km Bins and Inhibitor Validation

The table below provides specifications and data around each kinase. The representative IC50 value with a known inhibitor (Staurosporine) for each kinase was determined at the ATP bin nearest to the ATP Km app, unless indicated with an asterisk (*) in which case the IC_50_ value was determined at 100 µM ATP.

| **Assay** | **Z'-LYTE Substrate** | **ATP Km app (µM)** | **ATP Bin (µM)** | **IC_50_ (nM)** |
| --- | --- | --- | --- | --- |
| BRAF | Ser/Thr 03 | cascade | 100 | 17.3 * |
| FLT1 (VEGFR1) | Tyr 04 | 158 | 150 | 24.4 |
| KDR (VEGFR2) | Tyr 01 | 78 | 75 | 5.79 |
| KIT | Tyr 06 | 284 | 300 | 492 |
| MET (cMet) | Tyr 06 | 64 | 50 | 99.1 |
| PDGFRA (PDGFR alpha) | Tyr 04 | 9 | 10 | 5.19 |
| RET | Tyr 02 | 11 | 10 | 2.89 |


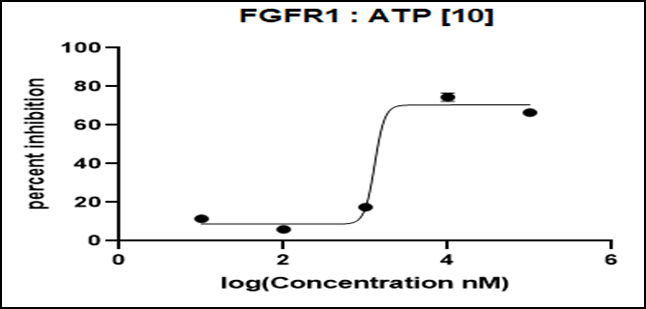

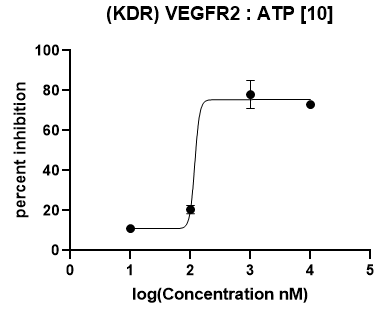


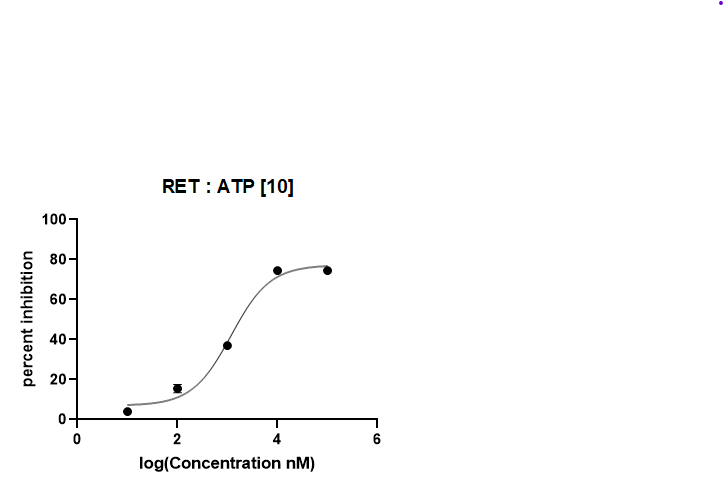


**Figure S16.** Inhibitory concentration effect (IC_50_) of the targeted compound **15c** on the FGFR1, VEGFR-2 and RET kinases activity.

**References**

(1) Sippel, K. H.; Robbins, A. H.; Domsic, J.; Genis, C.; Agbandje-McKenna, M.; McKenna, R. High-resolution structure of human carbonic anhydrase II complexed with acetazolamide reveals insights into inhibitor drug design. *Acta Crystallographica Section F: Structural Biology and Crystallization Communications* **2009**, *65*, 992-995.

(2) Leitans, J.; Kazaks, A.; Balode, A.; Ivanova, J.; Zalubovskis, R.; Supuran, C. T.; Tars, K. Efficient expression and crystallization system of cancer-associated carbonic anhydrase isoform IX. *Journal of Medicinal Chemistry* **2015**, *58*, 9004-9009.

(3) Whittington, D. A.; Waheed, A.; Ulmasov, B.; Shah, G. N.; Grubb, J. H.; Sly, W. S.; Christianson, D. W. Crystal structure of the dimeric extracellular domain of human carbonic anhydrase XII, a bitopic membrane protein overexpressed in certain cancer tumor cells. *Proceedings of the National Academy of Sciences* **2001**, *98*, 9545-9550.

(4) McTigue, M.; Murray, B. W.; Chen, J. H.; Deng, Y.-L.; Solowiej, J.; Kania, R. S. Molecular conformations, interactions, and properties associated with drug efficiency and clinical performance among VEGFR TK inhibitors. *Proceedings of the National Academy of Sciences* **2012**, *109*, 18281-18289.

(5) Tucker, J. A.; Klein, T.; Breed, J.; Breeze, A. L.; Overman, R.; Phillips, C.; Norman, R. A. Structural insights into FGFR kinase isoform selectivity: diverse binding modes of AZD4547 and ponatinib in complex with FGFR1 and FGFR4. *Structure* **2014**, *22*, 1764-1774.

(6) Terzyan, S. S.; Shen, T.; Liu, X.; Huang, Q.; Teng, P.; Zhou, M.; Hilberg, F.; Cai, J.; Mooers, B. H.; Wu, J. Structural basis of resistance of mutant RET protein-tyrosine kinase to its inhibitors nintedanib and vandetanib. *Journal of Biological Chemistry* **2019**, *294*, 10428-10437.

(7) Skehan, P.; Storeng, R.; Scudiero, D.; Monks, A.; McMahon, J.; Vistica, D.; Warren, J. T.; Bokesch, H.; Kenney, S.; Boyd, M. R. New colorimetric cytotoxicity assay for anticancer-drug screening. *JNCI: Journal of the National Cancer Institute* **1990**, *82*, 1107-1112.

(8) Allam, R. M.; Al-Abd, A. M.; Khedr, A.; Sharaf, O. A.; Nofal, S. M.; Khalifa, A. E.; Mosli, H. A.; Abdel-Naim, A. B. Fingolimod interrupts the cross talk between estrogen metabolism and sphingolipid metabolism within prostate cancer cells. *Toxicology Letters* **2018**, *291*, 77-85.

(9)[https://www.thermofisher.com/eg/en/home/products-and-services/services/custom-services/screening-and-profiling- services/selectscreen-profiling-service/selectscreen-kinase-profiling-service.html](https://www.thermofisher.com/eg/en/home/products-and-services/services/custom-services/screening-and-profiling-%20services/selectscreen-profiling-service/selectscreen-kinase-profiling-service.html).
